# Synthesis, Characterization and Catalytic Activity of Iron, Cobalt and Nickel Complexes Bearing an N‑Heterocyclic Carbene-Based PCP Pincer Ligand

**DOI:** 10.1021/acs.organomet.5c00172

**Published:** 2025-05-28

**Authors:** Tiago F. C. Cruz, Daniel P. Zobernig, Berthold Stöger, Ernst Pittenauer, Karl Kirchner

**Affiliations:** † Institute of Applied Synthetic Chemistry, TU Wien Getreidemarkt 9/163-AC, A-1060 Wien, Austria; ‡ Centro de Química Estrutural, Institute of Molecular Sciences, Departamento de Engenharia Química, Instituto Superior Técnico, Universidade de Lisboa, Av. Rovisco Pais, 1049 001 Lisboa, Portugal; § X-Ray Center, TU Wien Getreidemarkt 9/163, A-1060 Wien, Austria; ∥ Institute of Chemical Technologies and Analytics, TU Wien Getreidemarkt 9, A-1060 Vienna, Austria

## Abstract

Reactions of the bis­((R_2_phosphanyl)­methyl)-1*H*-benzo­[*d*]­imidazole-3-ium hexafluorophosphate precursors [(PCP-R)­H]­PF_6_ (R = *i*Pr, Ph) with zerovalent precursors [Fe_3_(CO)_12_], [Co_2_(CO)_8_], [Ni­(COD)_2_] and [Ni­(PPh_3_)_4_], respectively, gave rise to the cationic iron, cobalt and nickel complexes [Fe­(PCP-*i*Pr)­(CO)_2_H]­PF_6_ (**1**), [Co­(PCP-*i*Pr)­(CO)_2_]­PF_6_ (**2**), [Co­(PCP-Ph)­(CO)_2_]­PF_6_ (**3**), [Ni­(PCP-*i*Pr)­(cyclooct-4-en-1-yl)]­PF_6_ (**4**) and [Ni­(PCP-*i*Pr)­H]­PF_6_ (**5**), by oxidative addition of the benzimidazolium CH bond in [(PCP-R)­H]­PF_6_. The complexes bearing the bidentate ligand 3-((diisopropylphosphanyl)­methyl)-1-methyl-1H-benzo­[*d*]­imidazolidene PC-*i*Pr [Fe­(PC-*i*Pr)­(CO)_3_] (**6**) and [Fe­(PC-*i*Pr)­(CO)_3_H]­BF_4_ (**7**) were also synthesized. All complexes were characterized by NMR and FTIR spectroscopies, high resolution mass spectrometry and selected cases by single-crystal X-ray diffraction. Cobalt complexes **2** and **3** were catalytically active in the hydroboration of styrene with pinacolborane (HBPin) using 1 mol % of precatalyst and 2 mol % of KO*t*Bu in THF at 70 °C for 18 h with yields of 87–93%. In addition, complex **2** also catalyzed the hydroboration of terminal alkenes in good yields (68–88%). Reaction of complex **2** with 5 equivs of HBPin and 2 equivs of KO*t*Bu in THF gave rise to the cobalt­(I) hydride complex [Co­(κ^2^-(P,C)-PCP-*i*Pr)­H­(CO)_2_] (**8**), indicating that the mechanism of the catalytic process follows a cobalt­(I) hydride pathway.

## Graphical abstract

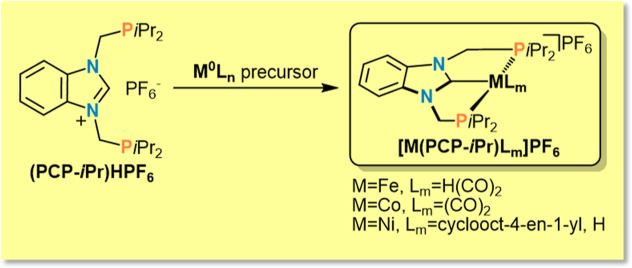

## Introduction

Complexes featuring pincer ligands have been widely reported in the literature.^1^ The high tunability of pincer ligands allow for the isolation of wide varieties of transition metal complexes and thus probe their stoichiometric and catalytic reactivity.^2^ Complexes of PCP pincer ligands, in which the ligand coordinates to the metal center through one carbon and two phosphines, consisting of a benzene backbone with two phosphine donors with O, NR or CR_2_ linkers are a very interesting class of pincer systems. Of those, those using CR_2_ linkers are among the most common (Chart 1A).^3^

**Chart 1:**
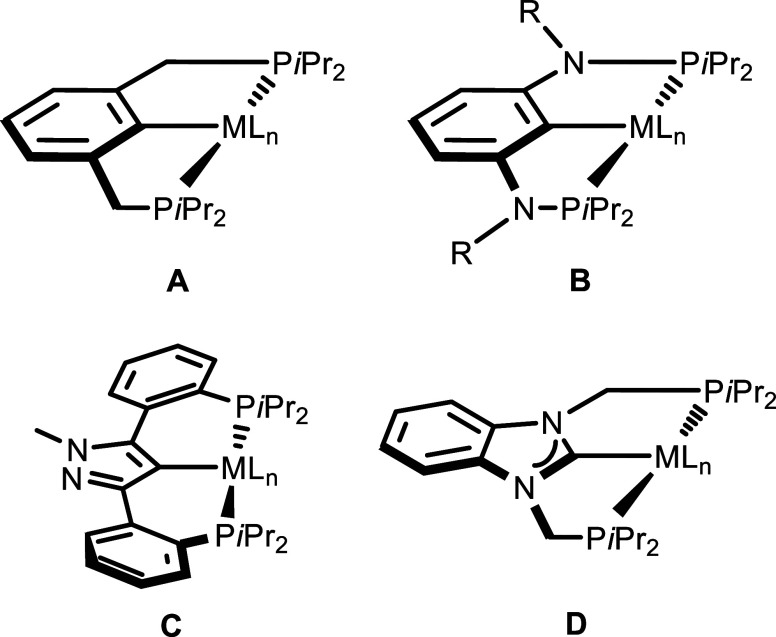
Examples of PCP pincer complexes containing anionic (A–C) and neutral (D) donors.

Our research group has developed extensive chemistry on PCP ligands over the years using a benzene backbone with NR linkers, from which a wide variety of iron,^4^ cobalt,^5,6^ and nickel^6^ complexes have been prepared and characterized (Chart 1B). Such PCP complexes have found numerous applications in diverse catalytic reactions, such as hydrogenation, hydroboration and hydrosilylation of various classes of organic substrates. In addition, our group also found out that the chelating ipso carbon of the PCP chelate is susceptible to protonation, giving rise to a less conventional agostic interaction involving the hydrogen atom of the CH bond therein formed. The first example of this bonding using PCP complexes was observed in a cobalt complex.^7^ Lately, a PCP ligand employing a pyrazole backbone and two phosphine functions with phenylene linkers (Chart 1C)^8^ also allowed the synthesis and characterization of several first-row transition metal complexes, of which the iron ones served as precatalysts for the transfer semihydrogenation^9^ or hydrosilylation^10^ of alkynes.

The particularity of such PCP pincer systems is that, in their most conventional form, they operate as anionic ligands, their chemistry with monovalent metal complexes becoming more limited. Considering this, our group envisioned that by using a N-heterocyclic carbene in the ligand backbone, the stabilization of monovalent metal centers would now be possible with a neutral ligand, while retaining the advantageous structural features demonstrated by the anionic pincer PCP ligands. Fortunately, Nishibayashi and co-workers had already prepared a benzo­[*d*]­imidazolidene with two CH_2_P*t*Bu_2_ side arms^11^ and had explored its Ru,^12^ Ir,^13^ and Mo^14^ coordination chemistries, the latter with applications in the reduction of dinitrogen to ammonia. Variants of this ligand, namely using a saturated imidazolidene diphosphine with nickel^15^ or an unsaturated imidazolidene diphosphine with manganese^16^ had already been reported in the literature. In addition, the dicarbonyl cobalt­(I) complexes containing an unsaturated imidazolidene diphosphine with ethylene linkers between the N-heterocyclic carbene and phosphine moieties were also previously reported.^17^ Also, de Ruiter and co-workers developed a new rigid PCP ligand based on a dipyridoimidazolinylidene diphosphine framework and used it to prepare iron,^18^ manganese,^19^ and cobalt^20^ complexes, with diverse catalytic applications.

Picking up on the synthetic efforts of Nishibayashi, we recently synthesized a benzo­[*d*]­imidazolidene ligand with two CH_2_P*i*Pr_2_ side arms and prepared the corresponding PCP manganese alkyl and hydride complexes (Chart 1D). These complexes were active precatalysts for the hydrogenation of alkenes^21,22^ and the hydroboration of terminal alkynes.^23^ The starting point of this work consisted in extending the coordination chemistry of this N-heterocyclic carbene-based PCP ligand to other late first-row transition metals. In this work, we explore the possibility of oxidative addition of the CH bond in 1,3-bis­((R_2_phosphanyl)­methyl)-1H-benzo­[*d*]­imidazole-3-ium hexafluorophosphate (PCP-R)­HPF_6_ (R = *i*Pr, Ph) precursors onto different zerovalent iron, cobalt and nickel precursors. The complexes herein reported have been structurally characterized and their catalytic activity in alkene hydroboration is evaluated.

## Results and Discussion

The reactions of the benzimidazolium diphosphine [(PCP-R)­H]­PF_6_ (R = *i*Pr or Ph) ligand precursors with different zerovalent iron, cobalt and nickel precursors afforded the isolation of the PCP complexes **1**–**5** (Scheme 1).

**Scheme 1:**
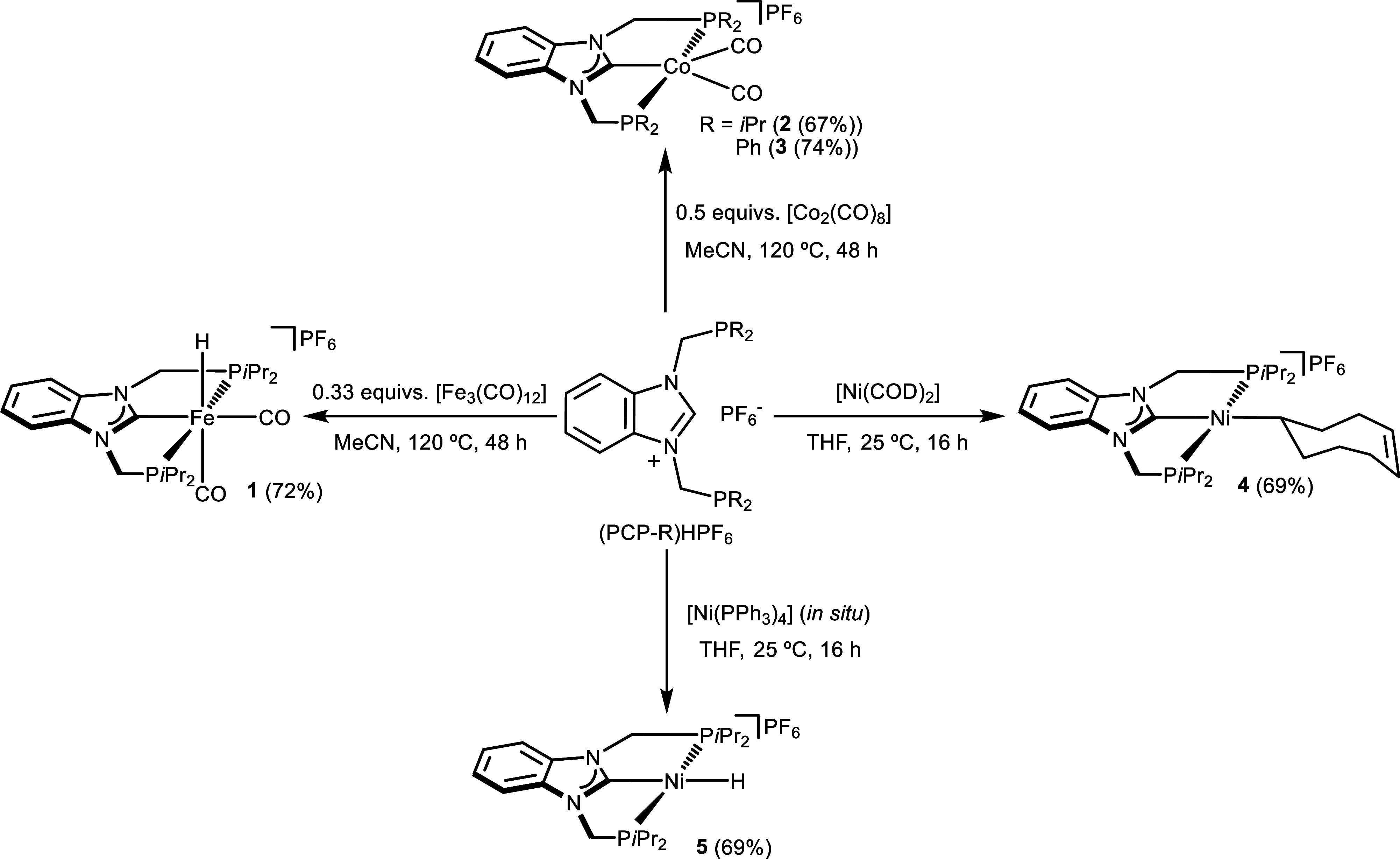
Synthesis of PCP Iron, Cobalt and Nickel Complexes **1**–**5**

With iron, the reaction of the [(PCP-*i*Pr)­H]­PF_6_ precursor with 0.33 equivs of [Fe_3_(CO)_12_] in acetonitrile at 120 °C for 48 h, gave rise to the cationic PCP dicarbonyl hydride iron complex [Fe­(PCP-*i*Pr)­(CO)_2_H]­PF_6_ (**1**). This hexacoordinated 18-electron complex was formed by the oxidative addition of the CH bond in the benzimidazole moiety of [(PCP-*i*Pr)­H]­PF_6_ onto the zerovalent iron centers of [Fe_3_(CO)_12_]. The reaction of [(PCP-Ph)­H]­PF_6_ precursor with 0.33 equivs of [Fe_3_(CO)_12_] led to intractable mixtures.

The solvothermal reaction of [(PCP-R)­H]­PF_6_ with 0.5 equivs of [Co_2_(CO)_8_] (under the same conditions used in the preparation of complex **1**) cleanly afforded the cationic Co­(I) complexes [Co­(PCP-*i*Pr)­(CO)_2_]­PF_6_ (**2**) and [Co­(PCP-Ph)­(CO)_2_]­PF_6_ (**3**). Similarly to complex **1**, the formation of complexes **2** and **3** likely involved the oxidative addition of the C–H bond in the benzimidazole moiety of the [(PCP-R)­H]^+^ precursor onto the zerovalent cobalt centers of [Co_2_(CO)_8_], giving rise to the thermally and kinetically unstable [Co­(PCP-R)­(CO)_2_H]­PF_6_ Co­(II) hydride complexes, which, upon liberation of molecular hydrogen, resulted in the formation of the isolated complexes.

When reacting the [(PCP-*i*Pr)­H]­PF_6_ precursor with one equivalent of [Ni­(COD)_2_] in THF at 25 °C for 16 h, the cationic PCP nickel cyclooct-4-en-1-yl complex [Ni­(PCP-*i*Pr)­(cyclooct-4-en-1-yl)]­PF_6_ (**4**) was formed. This complex again likely involved the oxidative addition of the CH bond in the benzimidazole moiety of the [(PCP-*i*Pr)­H]­PF_6_ precursor onto the zerovalent center of [Ni­(COD)_2_], giving rise to a putative cationic Ni­(II) hydride complex, which readily underwent a 1,2-insertion reaction of the 1,5-cyclooctadiene formed during the complexation reaction. The cationic Ni­(II) hydride complex [Ni­(PCP-*i*Pr)­H]­PF_6_ (5) was successfully synthesized by reaction of the [(PCP-*i*Pr)­H]­PF_6_ precursor with in situ prepared [Ni­(PPh_3_)_4_] (by reacting [Ni­(COD)_2_] with 4 equivs of triphenylphosphine), in THF at 25 °C for 16 h. Similarly to the iron case, the reaction of [(PCP-Ph)­H]­PF_6_ precursor the with zerovalent nickel precursor also led to intractable mixtures.

By contrast, when reacting the bidentating benzimidazolium phosphine PC ligand precursor 3-((diisopropylphosphanyl)­methyl)-1-methyl-1H-benzo­[*d*]­imidazolium iodide (PC-*i*Pr)­HI in the presence of KBF_4_ with zerovalent precursors, the formation of complexes analogous to complexes **1**–**5** was not observed, only intractable mixtures being instead obtained. This meant that the direct CH oxidative addition reaction is not the preferred pathway for the bidentate [(PC-*i*Pr)­H]­I precursor. This fact is explained by the lower chelation effect and worse σ-donation ability of a bidentate vs a tridentate benzimidazolium moiety. Nevertheless, it was still possible to prepare bidentate PC iron complexes **6** and **7** using the [(PC-*i*Pr)­H]I precursor (Scheme 2).

**Scheme 2:**
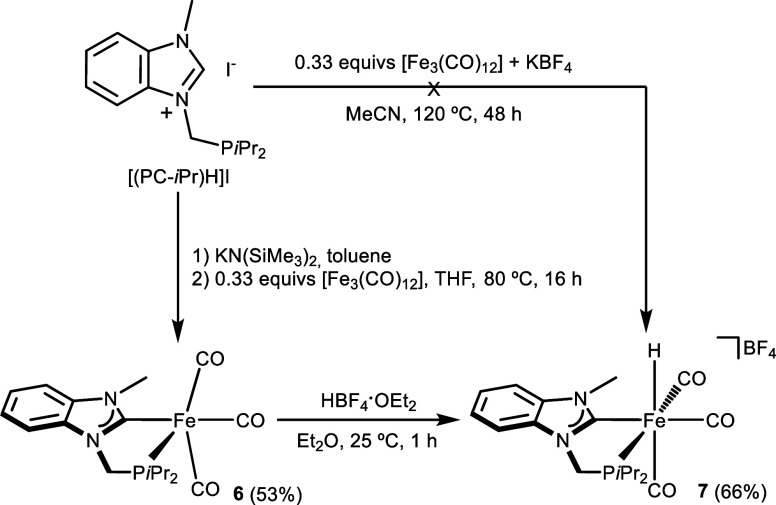
Synthesis of PC Iron Complexes **6** and **7**

The in situ deprotonation of [(PC-*i*Pr)­H]I with KN­(SiMe_3_)_2_ followed by reaction with 0.33 equivs of [Fe_3_(CO)_12_], in THF at 80 °C for 16 h, led to the isolation of the Fe(0) complex [Fe­(PC-*i*Pr)­(CO)_3_] (**6**). Subsequently, reaction of complex **6** with HBF_4_·OEt_2_, in diethyl ether at 25 °C for 1 h, led to the formation of the Fe­(II) cationic tricarbonyl hydride complex [Fe­(PC-*i*Pr)­H­(CO)_3_]­BF_4_ (**7**), via an oxidative protonation reaction.

All complexes were characterized by NMR and FTIR spectroscopies and high-resolution mass spectrometry, and selected cases by single-crystal X-ray diffraction. The NMR spectra of all complexes are presented in Figures S1–S28 of the Supporting Information.

The ^1^H and ^13^C NMR spectra of complexes **1**–**5** present the spectroscopic features of symmetric PCP-R (R = *i*Pr, Ph) moieties, in which one set of resonances are observed for the aromatic, CH_2_ and R groups. The ^31^P NMR spectra of the complexes present, aside from the PF_6_^–^ heptet centered at −144.6 ppm, single resonances in the range of 72.44–116.5 ppm, for complexes bearing the PCP-*i*Pr ligand, and a single resonance equal to 85.4 ppm, for complex **3**, bearing a PCP-Ph ligand, all of which significantly deshielded from the respective [(PCP-R)­H]­PF_6_ ligand precursors. The CH_2_, NCN, CO and *i*Pr carbon resonances are triplets due to carbon coupling to the two phosphorus nuclei of the PCP-R ligands. All the previous observations are in line with tridentate coordination modes of the PCP-R ligands. In addition, the ^1^H NMR spectra of the PCP hydride complexes **1** and **5** additionally present triplet resonances centered at −9.72 and −9.32 ppm, respectively, diagnostic of proton coupling to two phosphorus nuclei with ^2^ *J*_HP_ in the range of 42–48 Hz. This is indicative of the presence of a hydride moiety coordinated to a PCP-*i*Pr metal complex. Lastly, the ^1^H and ^13^C NMR resonances for the cyclooct-4-en-1-yl moiety are also present for complex **4**, where the coordinating carbon is a triplet, due to carbon–phosphorus coupling. Complexes **6** and **7** also reveal the spectral features of coordinated PC ligands, characterized by four different aromatic ^1^H and ^13^C NMR resonances and by ^31^P NMR chemical shifts in the range of 110.6–111.3 ppm. The CH_2_ resonances in complex **7** are diastereotopic. In addition, the hydride ligand in complex **7** is characterized by a doublet centered at −8.92 ppm, with a ^2^ *J*_HP_ equal to 48 Hz. The FTIR spectra of all CO-containing complexes also evidence the stretching bands of coordinated CO ligands in the range of 1943–2145 cm^–1^.

Complexes **1**, **2**, **4**, **6** and were analyzed by single-crystal X-ray diffraction and their molecular structures are presented in Figure 1.

**Figure 1:**
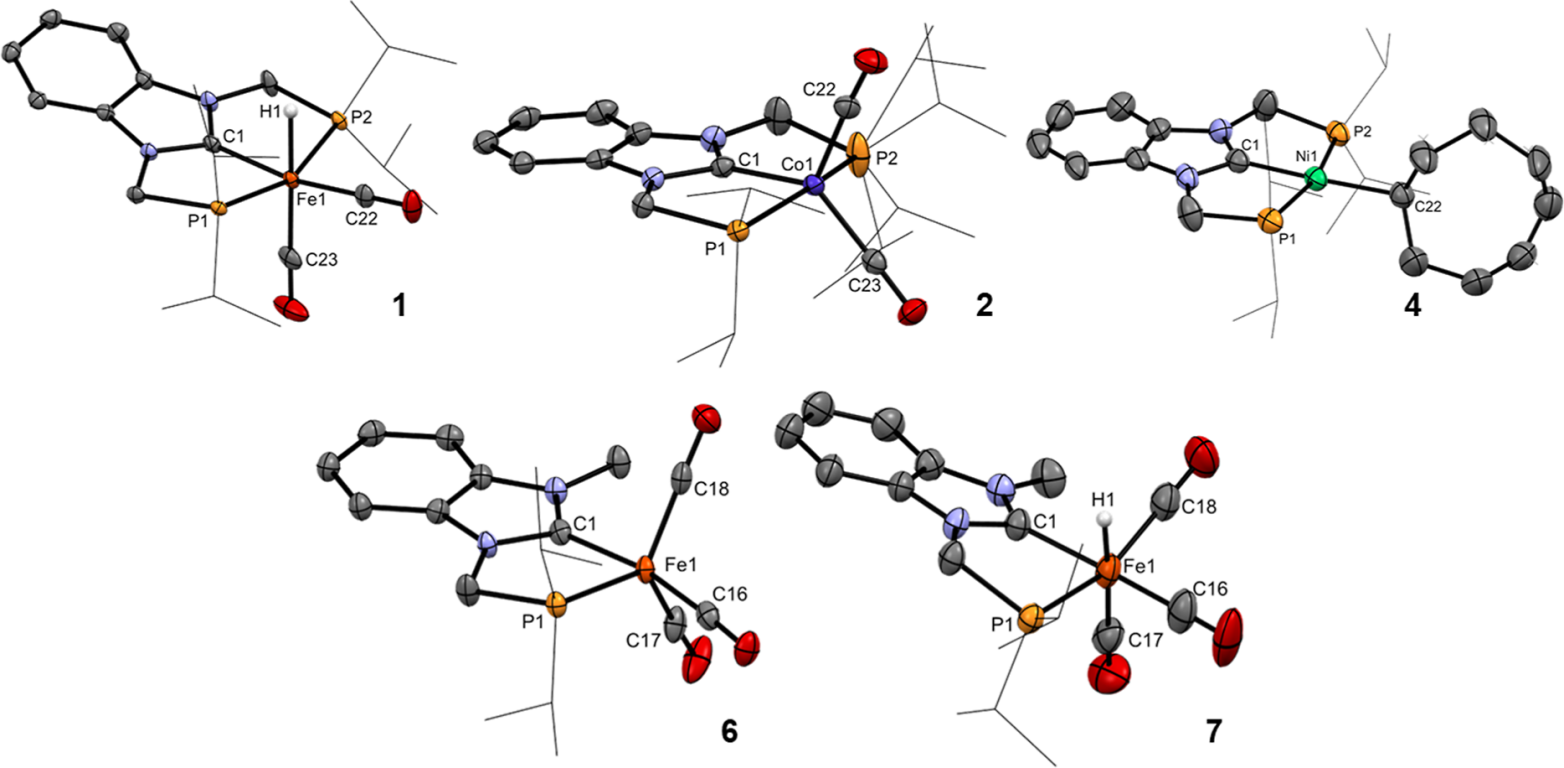
Single-crystal X-ray diffraction structures of complexes **1**, **2**, **4**, **6** and **7**, showing 50 or 30% probability ellipsoids. Most hydrogen atoms (except that of the hydride ligands when applicable) and the counteranions of the complexes (when applicable) were omitted and the *i*Pr groups were presented in wireframe mode for clarity.

The structures of complexes **1**, **2** and **4** present the PCP ligand respectively coordinated to iron, cobalt and nickel in a tridentate mode, in which two 5-membered chelation rings are evident. The M1-P and M1-C bond lengths and in the range of 2.201(2)–2.235(4) and 1.876(2)–1.923(2) Å, respectively. The 5-membered M1–P1–N1–C1 chelation planes in complexes **1**, **2** and **4** present slight deviations from planarity with respect to the aromatic benzimidazole moieties: the P*i*Pr_2_ synthons sit, on average, 0.2 Å above or below the respective imidazole rings. The molecular structure of the cation of the iron complex **1** further contains one hydride (Fe1–H bond length equal to 1.572(2) Å) and two carbonyl (Fe1–C bond lengths in the range of 1.779(2)–1.788(2) Å) ligands in a virtually octahedral coordination environment. Complex **1** is isostructural with the previously reported neutral PCP hydride manganese complex [Mn­(PCP-*i*Pr)­(CO)_2_H] and thus present very similar structural parameters.^21^ In addition, the structural features of complex **1** are in line with previously crystallographically characterized cationic hydride dicarbonyl diphosphine iron complexes.^24^ The cation of the cobalt complex **2** presents a pentacoordinate metal center with two further carbonyl (Co1–C bond lengths in the range of 1.763(2)–1.797(2) Å) ligands, in an intermediate geometry between square pyramidal and trigonal bipyramidal (τ_5_ = 0.39 ^25^). The structural parameters of complex **2** are in line with previously crystallographically characterized cationic dicarbonyl diphosphine cobalt­(I) complexes.^17,26^ Lastly, the PCP nickel complex **4** further presents a η^1^-coordinated cyclooct-4-en-1-yl ligand adopting a chair configuration and the tetracoordinate nickel center displays a square planar coordination geometry (τ_4_ = 0.14 ^27^). Complex **4** is only the second crystallographically characterized nickel cyclooct-4-en-1-yl complex, after that of Liang and co-workers.^28^

The iron complexes **6** and **7** present the PC-*i*Pr ligand in a bidentate coordination mode, in which 5-membered chelation rings are also evident, with Fe1–P and Fe1–C bond lengths in the range of 2.206(7)–2.244(2) and 1.960(2)–1.979(5) Å, respectively. Aside from the PC ligand, complexes **6** and **7** present three carbonyl ligands (Fe1–C bond lengths in the range of 1.759(3)–1.805(6) Å) and complex **7** a further hydride ligand (Fe1–H bond length equal to 1.42(4) Å). The iron center in the pentacoordinate complex **6** presents a nearly trigonal bipyramidal coordination geometry (τ_5_ = 0.86,^25^ which is the average value of the two molecules present in the asymmetric unit), while the hexacoordinate complex **7** displays a virtually octahedral coordination geometry.

With the newly synthesized complexes in hand, we envisioned their use as catalysts in the hydrosilylation or hydroboration of alkenes and alkynes. Of all the newly synthesized complexes, only the cobalt­(I) complexes **2** and **3** were active in the hydroboration of styrene with pinacolborane (HBPin), utilizing loadings of 1 mol %, in the presence of 2 mol % of KO*t*Bu and performing the reactions in THF at 70 °C for 18 h. By utilizing such reaction conditions, the respective *anti*-Markovnikov alkyl boronate ester was the only observed product in yields of 87 and 93%, for complexes **2** and **3**, respectively. The systems composed by 1 mol % of **2** or **3** (without KO*t*Bu) or 2 mol % of KO*t*Bu (without complexes **2** or **3**) or in the absence of both complexes **2** or **3** and KO*t*Bu did not catalyze the hydroboration of styrene under the stated reaction conditions. Performing the catalytic reaction with 1 mol % of complex **2** and 2 mol % of KO*t*Bu at room temperature also did not lead to any catalytic activity. Given the success of the cobalt complexes **2** and **3** in the hydroboration of styrene, we have explored the respective substrate scope using complex **2** as the precatalyst (Table 1).

**Table 1:**
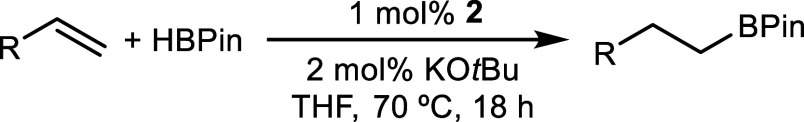

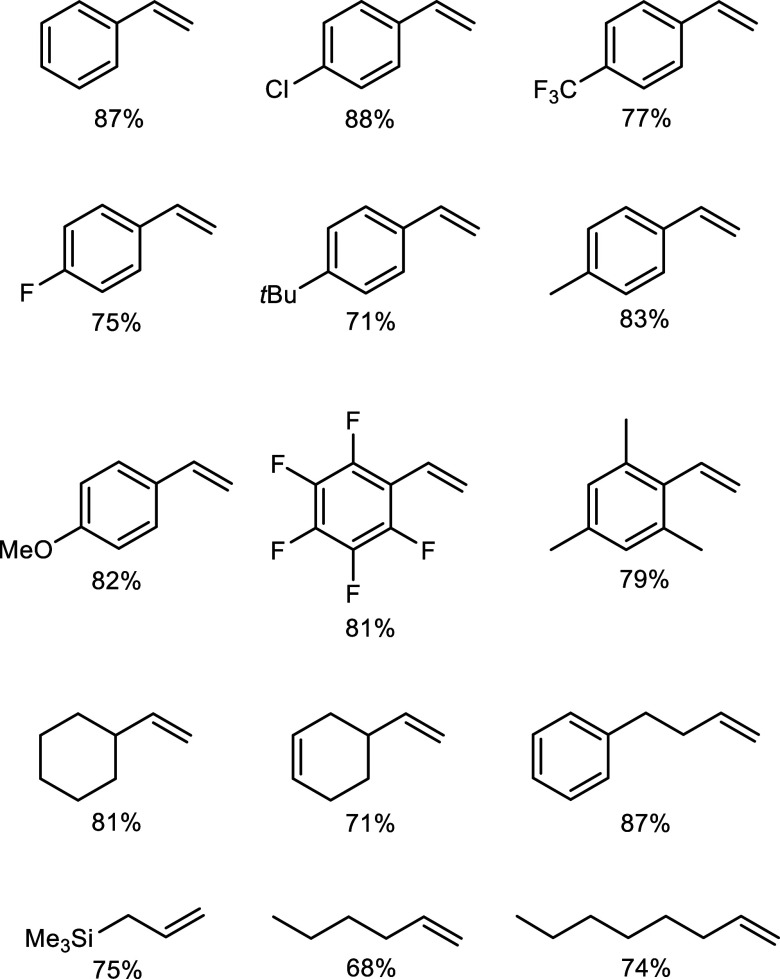
Hydroboration of Alkenes Catalyzed by Complex **2** ^a^

a Values denote isolated yields of the respective hydroboration products. Conditions: 1 mol % of complex **2**, 2 mol % of KO*t*Bu, Solvent: THF (0.5 mL), Temperature: 70 °C, Time: 18 h.

Table 1 shows that the system composed by 1 mol % of **2** and 2 mol % of KO*t*Bu catalyzed the hydroboration of several terminal alkenes in high yields. The NMR spectra of the isolated hydroboration products are presented in Figures S32–S64 of the SI and agree with those reported in the literature.^29^ A variety of electronically (*para*-Cl, *para*-CF_3_, *para*-F, *para*-*t*Bu and *para*-Me) and sterically (C_6_F_5_, mesityl) differentiated styrenes were successfully converted to the respective boronate esters in high yields (71–87%). The catalyst system using 1 mol % of **2** and 2 mol % of KO*t*Bu also catalyzed the conversion of vinylcyclohexane and of the terminal unsaturation of 4-vinylcyclohex-1-ene in yields in the range of 71–87%, as well as but-3-en-1-ylcyclohexane in 87% yield. The fact that the internal unsaturation in 4-vinylcyclohex-1-ene remained unreacted throughout the catalytic run indicated the selectivity of this catalyst system toward terminal alkenes. Allyltrimethylsilane and aliphatic terminal alkenes such as 1-hexene and 1-octene were also converted into the respective hydroboration products in high yields (68–75%) by the present catalyst system. No olefin isomerization was ever observed during all catalytic runs. Though the catalyst system was able to convert substrates such as allylbenzene and 6-chlorohex-1-ene to the respective hydroboration products, it did not do so with complete selectivity, other unidentified products being also obtained. The hydroboration of the studied substrates always gave rise to complete *anti*-Markovnikov selectivity. Cobalt complexes have been known to catalyze the hydroboration of terminal alkenes, many of which outperforming the present catalyst system, by exhibiting catalytic activity in the absence of a base and, albeit at equal catalyst loadings, performing such transformations at room temperature.^30^

The reaction of complex **2** with 5 equivs of HBPin and 2 equivs of KO*t*Bu in THF at 25 °C for 10 min gave rise to the neutral cobalt­(I) dicarbonyl hydride complex [Co­(κ^2^-(P,C)-PCP-*i*Pr)­H­(CO)_2_] (**8**), in which one of the phosphine arms dissociated from the metal center and the PCP ligand is coordinated to cobalt in a bidentate mode along with the formation of *t*BuOBPin (Scheme 3).

**Scheme 3:**
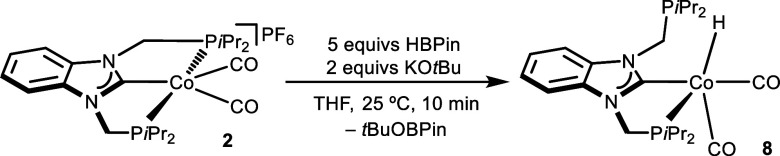
Reaction of Complex **2** With HBPin/KO*t*Bu: Detection of the Hydride Complex **8**

The identity of complex **8** was verified by NMR spectroscopy (see Figures S29–S31 of the SI). The ^1^H and ^13^C NMR spectra of complex **8** contained the expected resonances for an asymmetric benzimidazolidene moiety and two different methylenic linkers. In addition, the ^1^H NMR hydride resonance is a doublet centered at −12.29 ppm (^2^ *J*_HP_ = 54 Hz), indicating its coupling to a single phosphorus atom. The bidentate coordination of the PCP ligand in complex **8** is also apparent in its ^31^P NMR spectrum, where two resonances appear at 100.4 and −4.5 ppm, corresponding to the coordinated and free P*i*Pr_2_ moieties, respectively. The formation of *t*BuOBPin was confirmed by the presence of two singlets at 1.38 and 1.06 ppm in the ^1^H NMR spectrum and a resonance at 21.7 ppm in the ^11^B NMR spectrum of the crude reaction mixture. Reacting complex **3** with 5 equivs of HBPin and 2 equivs of KO*t*Bu in THF at 25 °C for 10 min also gave rise to the cobalt­(I) hydride complex [Co­(κ^2^-(P,C)-PCP-Ph)­H­(CO)_2_], along with other unidentified species. No reactions occurred when mixing iron complexes **1** and **7** or nickel complexes **4** or **5** with 5 equivs of HBPin and 2 equivs of KO*t*Bu in THF at 25 °C for 10 min.

Given the fact the hydride complex **8** was detected from the reaction of precatalyst **2** with HBPin/KO*t*Bu, the hydroboration reactions follow a hydride mechanism (Scheme 4).

**Scheme 4:**
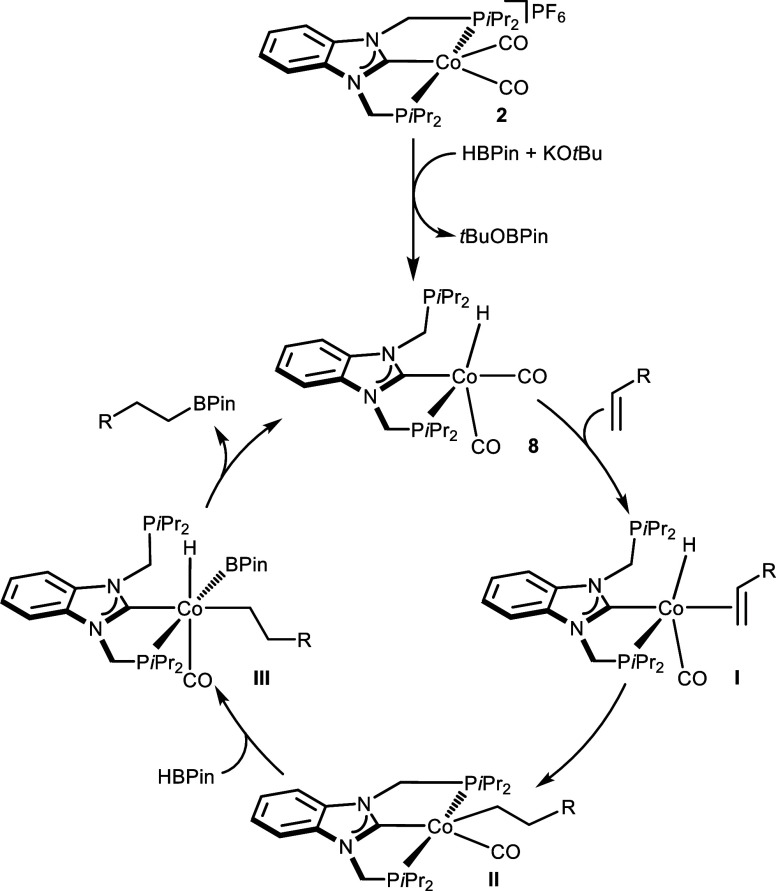
Mechanism for the Hydroboration of Alkenes Catalyzed by Complex **2**/KO*t*Bu

In this case, the 18-electron complex **8**, likely by dissociation of a CO ligand, coordinates the alkene substrate, giving rise to the hydride olefin intermediate [Co­(κ^2^-PCP-*i*Pr)­H­(CO)­(CH_2_=CHR)] (**I**), which readily undergoes a intramolecular migratory insertion reaction forming the alkyl intermediate [Co­(κ^3^-PCP-*i*Pr)­(CO)­(CH_2_CH_2_R)] (**II**), which is stabilized by the recoordination of a second phosphine ligand, i.e. the PCP ligand reassuming a tridentate coordination mode. The Co­(I) intermediate **II** may then react with HBPin yielding the Co­(III) intermediate [Co­(κ^2^-PCP-*i*Pr)­(CO)­(H)­(BPin)­(CH_2_CH_2_R)] (**III**) via oxidative addition across the BH bond of HBPin. Intermediate **III** then regenerates complex **8** via reductive elimination of the respective *anti*-Markovnikov alkyl boronate ester. A similar mechanism has been proposed by Karton and de Ruiter and co-workers when reporting cobalt-based catalyst systems supported by bipyridine-diimine tetradentate ligands.^31^ Alternatively, one cannot rule out the direct σ-bond metathesis reaction of intermediate **II** with HBPin, giving rise to the *anti*-Markovnikov alkyl boronate ester and regenerating the Co­(I) hydride complex **8**.

## Conclusion

We have described the synthesis, characterization and alkene hydroboration activity of a family of new iron, cobalt and nickel PCP tridentate or iron PC bidentate complexes of benzo­[*d*]­imidazolidene-phosphine ligands.

The new iron, cobalt and nickel PCP complexes have been synthesized by reactions of the precursors [(PCP-R)­H]­PF_6_ (R = *i*Pr or Ph) with appropriate zerovalent metal precursors, via oxidative addition reactions of the benzimidazolium CH bonds present in the former. The iron hydride complex **1** used precursor [Fe_3_(CO)_12_], the cobalt­(I) complexes **2** and **3** used precursor [Co_2_(CO)_8_], while the nickel cyclooct-4-en-1-yl complex **4** and the nickel hydride complex **5** respectively used precursors [Ni­(COD)_2_] and in situ prepared [Ni­(PPh_3_)_4_]. In addition, the deprotonated benzo­[*d*]­imidazolidene-phosphine bidentate PC ligand was used to prepare the iron(0) complex **6**, starting from [Fe_3_(CO)_12_], which was successfully protonated with HBF_4_·OEt_2_ to yield the cationic iron­(II) hydride complex **7**. Incidentally, complex **7** could not be synthesized via oxidative addition of the benzimidazolium CH bond in the reaction of [(PC-*i*Pr)­H]­I/KBF_4_ with [Fe_3_(CO)_12_]. All complexes were characterized by NMR and FTIR spectroscopies, mass spectrometry and selected cases by single-crystal X-ray diffraction.

The cobalt complexes **2** and **3**, respectively bearing PCP-*i*Pr and PCP-Ph ligands, were active in the hydroboration of styrene using 1 mol % loadings in the presence of 2 mol % of KO*t*Bu in THF at 70 °C for 18 h. Under these conditions, styrene was hydroborated to the respective alkyl boronate esters in yields of 87 and 93%, respectively, using complexes **2** and **3**. Complex **2** also catalyzed the hydroboration of 15 terminal alkenes in high yields (68–88%). Reaction of complex **2** with 5 equivs of HBPin and 2 equivs of KO*t*Bu in THF gave rise to the cobalt­(I) dicarbonyl hydride complex **8**. The latter result indicates that the catalytic reactions follow a hydride mechanism route.

## Experimental Section

### General Procedures

All operations were performed under an inert atmosphere of argon by using Schlenk techniques or in an argon-filled MBraun glovebox. The solvents were purified according to standard procedures. The deuterated solvents were purchased from Eurisotop and dried over 3 Å molecular sieves. Ligand precursors (PCP-*i*Pr)­HPF_6_,^11^ (PCP-Ph)­HPF_6_^11^ and (PC-*i*Pr)­HI^22^ were synthesized according to the literature.

^1^H, ^13^C­{^1^H}, ^11^B­{^1^H}, ^19^F­{^1^H}, ^31^P­{^1^H} NMR spectra were recorded on Bruker AVANCE 250 or AVANCE 600 spectrometers. ^1^H and ^13^C­{^1^H} NMR spectra were referenced internally to residual protiosolvent, and solvent resonances, respectively, and are reported relative to tetramethylsilane (δ = 0 ppm). ^19^F­{^1^H} NMR spectra were referenced externally to CFCl_3_ (δ = 0 ppm). ^11^B­{^1^H} NMR spectra were referenced externally to BF_3_·OEt_2_ in CDCl_3_ (δ = 0 ppm). ^31^P­{^1^H} NMR spectra were referenced externally to H_3_PO_4_ (85%) (δ = 0 ppm). All chemical shifts are quoted in δ (ppm) and coupling constants (J) in Hz with multiplicities abbreviated as br (broad), s (singlet), d (doublet), t (triplet), q (quartet), h (heptet) and m (multiplet). FTIR measurements were performed on a PerkinElmer ATR spectrometer.

High resolution-accurate mass spectra were recorded on an Agilent Technologies 6545 LC/QTOF hybrid tandem mass spectrometer (Agilent Technologies, Singapore) fitted with an AJS dual spray ESI-source. Measured accurate mass data of the [M]^+^ ions for confirming calculated elemental compositions were typically within ±3 ppm accuracy. Mass calibration was done with ESI-L LCMS Tuning Solution (Low Concentration Tuning Mix), a commercial mixture of betaine, hexamethoxy-phosphazene, *tris*(heptafluoropropyl)-1,3,5-triazine and partially fluorinated alkyloxyphosphazanes (United States Patent Number: 5,872,357, Agilent Technologies, Santa Clara, CA, USA).

### X-Ray Structure Determination

X-ray diffraction data of complexes **1**, **2**, **4**, **6**, and **7** (CSD 2434462–2434466) in a dry stream of nitrogen on a STOE STADIVARI diffractometer system equipped with a Dectris Eiger CdTe hybrid photon counting detector using Mo–Kα radiation (λ = 0.71073 Å, complex **1**) or Cu–Kα radiation (λ = 1.54186 Å, complexes **2**, **4**, **6** and **7**). Generally, data were collected at T = 100 K, except for **7** (*T* = 250 K) owing to a phase transition on further cooling. Data were reduced with X-Area or Integrate3d and a correction for absorption effects was applied using the multiscan approach implemented in LANA.^32^ The structures were solved by the dual-space approach implemented in SHELXT^33^ and refined against F2 with SHELXL.^33^ H atoms connected to C were placed at calculated positions and thereafter refined as riding on the parent atom. The hydride H in complex **1** was located in difference Fourier maps and its position refined freely. Molecular graphics were generated with the program MERCURY.^34^

### Synthesis

#### [Fe­(PCP-*i*Pr)­H­(CO)_2_]­PF_6_ (**1**)

Inside an argon-filled glovebox, a microwave vial was charged with [(PCP-*i*Pr)­H]­PF_6_ (0.050 g, 0.096 mmol), [Fe_3_(CO)_12_] (0.018 g, 0.035 mmol) and acetonitrile (4 mL). The vial was capped, and the mixture was heated to 120 °C for 48 h. The resulting pale green cloudy solution was filtered, the solution concentrated, and diethyl ether was added with vigorous stirring, precipitating a pale green solid. The supernatant was decanted off and the pale green solid was dried under vacuum. Crystals suitable for single-crystal X-ray diffraction were obtained by slow diffusion of diethyl ether onto a concentrated acetonitrile solution. Yield: 0.044 g (72%). ^1^H NMR (600 MHz, CD_3_CN): δ 7.47 (m, 2H, aromatic H), 7.39 (m, 2H, aromatic H), 4.60 (dd, *J* = 12, 6 Hz, 2H, N–C*H*_2_–P), 4.48 (d, *J* = 18 Hz, 2H, N–C*H*_2_–P), 2.63 (m, 4H, P–C*H*(CH_3_)_2_), 1.49–1.40 (m, 12H, P–CH­(C*H*_3_)_2_), 1.27–1.20 (m, 6H, P–CH­(C*H*_3_)_2_), 1.03–0.96 (m, 6H, P–CH­(C*H*_3_)_2_), −9.72 (t, 1H, *J* = 42 Hz, Fe–*H*). ^13^C­{^1^H} NMR (151 MHz, CD_3_CN): δ 214.4 (t, *J* = 15 Hz, Fe–*C*O), 210.8 (t, *J* = 15 Hz, Fe–*C*O), 207.9 (t, *J* = 18 Hz, N*C*N), 135.7 (t, *J* = 4.5, aromatic C), 124.8 (aromatic *C*H), 112.3 (aromatic *C*H), 47.5 (d, *J* = 12 Hz, N–*C*H_2_–P), 47.4 (d, *J* = 12 Hz, N–*C*H_2_–P), 30.3 (t, *J* = 11 Hz, P–*C*H­(CH_3_)_2_), 27.0 (t, *J* = 11 Hz, P–*C*H­(CH_3_)_2_), 19.2 (P–CH­(*C*H_3_)_2_), 18.8 (P–CH­(*C*H_3_)_2_), 18.8 (P–CH­(*C*H_3_)_2_), 18.3 (P–CH­(*C*H_3_)_2_). ^19^F­{^1^H} NMR (564 MHz, CD_3_CN): δ −72.9 (d, *J* = 700 Hz, P*F*_6_^–^). ^31^P­{^1^H} NMR (243 MHz, CD_3_CN): δ 116.5 (*P*–Fe–*P*), −144.6 (h, *J* = 700 Hz, *P*F_6_^–^). FTIR (cm^–1^): 1966 (CO), 2012 (CO). HR-MS: *m*/*z* calcd for C_23_H_37_FeN_2_O_2_P_2_ [M]^+^ 491.1674, found 491.1684.

#### [Co­(PCP-*i*Pr)­(CO)_2_]­PF_6_ (**2**)

Inside an argon-filled glovebox, a microwave vial was charged with [(PCP-*i*Pr)­H]­PF_6_ (0.100 g, 0.191 mmol), [Co_2_(CO)_8_] (0.029 g, 0.100 mmol) and acetonitrile (4 mL). The vial was capped, and the mixture was heated to 120 °C for 48 h. The resulting pale-yellow cloudy solution was filtered, and all volatile materials were removed under vacuum, giving rise to a yellow solid. Crystals suitable for single-crystal X-ray diffraction were obtained from a concentrated acetonitrile solution at −20 °C. Yield: 0.082 g (67%). ^1^H NMR (600 MHz, CD_3_CN): δ 7.45–7.41 (m, 2H, aromatic H), 7.39–7.35 (m, 2H, aromatic H), 4.54 (m, 4H, N–C*H*_2_–P), 2.66 (m, 4H, P–C*H*(CH_3_)_2_), 1.34–1.22 (m, 24H, P–CH­(C*H*_3_)_2_). ^13^C­{^1^H} NMR (151 MHz, CD_3_CN): δ 202.7 (br, Co–*C*O), 200.7 (t, *J* = 24 Hz, N*C*N), 135.1 (t, *J* = 5 Hz, aromatic C), 124.7 (aromatic CH), 112.2 (aromatic CH), 45.2 (d, *J* = 14 Hz, N–*C*H_2_–P), 45.1 (d, *J* = 15 Hz, N–*C*H_2_–P), 28.5 (t, *J* = 14 Hz, P–*C*H­(CH_3_)_2_), 18.3 (P–CH­(*C*H_3_)_2_), 18.1 (P–CH­(*C*H_3_)_2_). ^19^F­{^1^H} NMR (564 MHz, CD_3_CN): δ −72.9 (d, *J* = 705 Hz, P*F*_6_^–^). ^31^P­{^1^H} NMR (243 MHz, CD_3_CN): δ 113.1 (*P*–Co–*P*), −144.6 (h, *J* = 712 Hz, *P*F_6_^–^). FTIR (cm^–1^): 1943 (CO), 1994 (CO). HR-MS: *m*/*z* calcd for C_23_H_36_CoN_2_O_2_P_2_ [M]^+^ 493.1579, found 493.1589.

#### [Co­(PCP-Ph)­(CO)_2_]­PF_6_ (**3**)

Inside an argon-filled glovebox, a microwave vial was charged with [(PCP-*i*Pr)­H]­PF_6_ (0.100 g, 0.151 mmol), [Co_2_(CO)_8_] (0.028 g, 0.083 mmol) and acetonitrile (4 mL). The vial was capped, and the mixture was heated to 120 °C for 48 h. The resulting dark yellow-brown cloudy solution was filtered, and all volatile materials were removed under vacuum, giving rise to a dark yellow-brown solid. Yield: 0.087 g (74%). ^1^H NMR (600 MHz, CD_3_CN): δ 7.65–7.52 (m, 20H, P–C_6_ *H*_5_), 7.52–7.50 (m, 2H, aromatic H), 7.44–7.41 (m, 2H, aromatic H), 5.31 (m, 4H, N–C*H*_2_–P). ^13^C­{^1^H} NMR (151 MHz, CD_3_CN): δ 200.6 (br, Fe–CO), 199.9 (br, N*C*N), 135.5 (t, *J* = 5 Hz, aromatic C), 133.1 (br, P–*C*_6_H_5_), 132.9 (t, *J* = 6 Hz, P–*C*_6_H_5_), 130.4 (t, *J* = 6 Hz, P–*C*_6_H_5_), 125.2 (aromatic CH), 112.5 (aromatic CH), 53.4 (t, *J* = 20 Hz, N–*C*H_2_–P). ^19^F­{^1^H} NMR (564 MHz, CD_3_CN): δ −72.9 (d, *J* = 705 Hz, P*F*_6_^–^). ^31^P­{^1^H} NMR (243 MHz, CD_3_CN): δ 85.4 (*P*–Co–*P*), −144.6 (h, *J* = 697 Hz, *P*F_6_^–^). FTIR (cm-1): 1965 (CO), 2034 (CO). HR-MS: *m*/*z* calcd for C_35_H_28_CoN_2_O_2_P_2_ [M]^+^ 629.0953, found 629.0958.

#### [Ni­(PCP-*i*Pr)­(cyclooct-4-en-1-yl)]­PF_6_ (**4**)

Inside an argon-filled glovebox, a vial was charged with [(PCP-*i*Pr)­H]­PF_6_ (0.100 g, 0.191 mmol), [Ni­(COD)_2_] (0.052 g, 0.191 mmol) and THF (4 mL). The vial was capped, and the mixture stirred at room temperature for 16 h. The resulting pale-yellow cloudy solution was filtered, and all volatile materials were removed under vacuum, giving rise to a yellow solid. Crystals suitable for single-crystal X-ray diffraction were obtained by slow diffusion of diethyl ether onto a concentrated THF solution. Yield: 0.091 g (69%). 1H NMR (600 MHz, THF-*d*_8_): δ 7.70–7.66 (m, 2H, aromatic H), 7.39–7.35 (m, 2H, aromatic H), 5.67 (td, *J* = 12 and 6 Hz, 1H, olefinic cyclooct-4-en-1-yl H), 5.58 (td, *J* = 12 and 6 Hz, 1H, olefinic cyclooct-4-en-1-yl H), 4.67 (m, 2H, N–C*H*_2_–P), 4.57 (m, 2H, N–C*H*_2_–P), 2.76 (m, 2H, P–C*H*(CH_3_)_2_), 2.65 (m, 2H, P–C*H*(CH_3_)_2_), 2.42 (m, 1H, methylenic cyclooct-4-en-1-yl H), 2.33 (m, 1H, methylenic cyclooct-4-en-1-yl H), 2.22 (m, 1H, methylenic cyclooct-4-en-1-yl H), 2.10–1.90 (m, 4H, Ni–C*H* + methylenic cyclooct-4-en-1-yl H), 1.62 (m, 2H, methylenic cyclooct-4-en-1-yl H), 1.52–1.24 (m, 26H, P–CH­(C*H*_3_)_2_ + methylenic cyclooct-4-en-1-yl H). ^13^C­{^1^H} NMR (151 MHz, THF-*d*_8_): δ 199.2 (t, *J* = 15 Hz, NCN), 135.8 (t, *J* = 5 Hz, aromatic C), 131.4 (olefinic cyclooct-4-en-1-yl C), 130.5 (olefinic cyclooct-4-en-1-yl C), 125.4 (aromatic CH), 113.5 (aromatic CH), 45.2 (d, *J* = 11 Hz, N–*C*H_2_–P), 41.9 (t, *J* = 5 Hz, methylenic cyclooct-4-en-1-yl C), 35.8 (methylenic cyclooct-4-en-1-yl C), 32.1 (methylenic cyclooct-4-en-1-yl C), 30.5 (methylenic cyclooct-4-en-1-yl C), 29.0 (methylenic cyclooct-4-en-1-yl C), 26.7 (d, *J* = 20 Hz, P–*C*H­(CH_3_)_2_), 20.0 (t, *J* = 14 Hz, Ni–*C*H), 19.5 (P–CH­(*C*H_3_)_2_), 19.0 (P–CH­(*C*H_3_)_2_), 18.7 (P–CH­(*C*H_3_)_2_), 17.8 (P–CH­(*C*H_3_)_2_). ^19^F­{^1^H} NMR (564 MHz, THF-*d*_8_): δ −72.7 (d, *J* = 710 Hz, P*F*_6_^–^). ^31^P­{^1^H} NMR (243 MHz, THF-*d*_8_): δ 72.4 (*P*–Ni–*P*), −144.1 (h, *J* = 710 Hz, *P*F_6_^–^). HR-MS: *m*/*z* calcd for C_29_H_49_N_2_NiP_2_ [M]^+^ 545.2719 found 545.2724.

#### [Ni­(PCP-*i*Pr)­H]­PF_6_ (**5**)

Inside an argon-filled glovebox, a vial was charged with [Ni­(COD)_2_] (0.026 g, 0.096 mmol), triphenylphosphine (0.101 g, 0.382 mmol) and toluene (3 mL). The vial was capped, and the mixture stirred at room temperature for 1 h, whereupon a dark red-brown solution formed. All volatile materials were evaporated to dryness and the golden brown solid dried under vacuum. To this solid, [(PCP-*i*Pr)­H]­PF_6_ (0.050 g, 0.096 mmol) was added and the mixture was dissolved in THF (4 mL) and stirred at room temperature for 16 h. The resulting pale-yellow cloudy solution was filtered, concentrated and 5 mL of diethyl ether were added with vigorous stirring, giving rise to a yellow solid. The supernatant was decanted off and the solid washed with a further 5 mL of diethyl ether and the yellow solid was dried under vacuum. Yield: 0.039 g (69%). ^1^H NMR (600 MHz, THF-*d*_8_): δ 7.75–7.70 (m, 2H, aromatic H), 7.43–7.39 (m, 2H, aromatic H), 4.79 (m, 4H, N–C*H*_2_–P), 2.58 (m, 4H, P–C*H*(CH_3_)_2_), 1.33–1.27 (m, 12H, P–CH­(C*H*_3_)_2_), 1.27–1.21 (m, 12H, P–CH­(C*H*_3_)_2_), −9.32 (t, 1H, *J* = 48 Hz, Ni–*H*). ^13^C­{^1^H} NMR (151 MHz, THF-*d*_8_): δ 199.7 (t, *J* = 18 Hz, N*C*N), 135.8 (t, *J* = 5 Hz, aromatic C), 125.6 (aromatic CH), 114.0 (aromatic CH), 46.1 (t, *J* = 14 Hz, N–*C*H_2_–P), 25.8 (t, *J* = 14 Hz, P–*C*H­(CH_3_)_2_), 19.3 (P–CH­(*C*H_3_)_2_), 18.8 (P–CH­(*C*H_3_)_2_). ^19^F­{^1^H} NMR (564 MHz, THF-*d*_8_): δ −72.7 (d, *J* = 710 Hz, P*F*_6_^–^). ^31^P­{^1^H} NMR (243 MHz, THF-*d*_8_): δ 91.1 (*P*–Ni–*P*), −144.1 (h, *J* = 710 Hz, *P*F_6_^–^). HR-MS: *m*/*z* calcd for C_21_H_37_N_2_NiP_2_ [M]+ 437.1780, found 437.1762.

#### [Fe­(PC-*i*Pr)­(CO)_3_] (**6**)

Inside an argon-filled glovebox, a microwave vial was charged with [(PCP-*i*Pr)­H]­PF_6_ (0.100 g, 0.256 mmol), K­[N­(SiMe_3_)_2_] (0.056 g, 0.282 mmol), [Fe_3_(CO)_12_] (0.047 g, 0.094 mmol) and THF (4 mL). The vial was capped, and the mixture was heated to 80 °C for 16 h. The resulting red-brown suspension was filtered, and all volatile materials were removed under vacuum. The residue was extracted with diethyl ether, and the volatiles of the combined extracts were removed under reduced pressure. The residue was dissolved in a minimal amount of toluene and double layered with *n*-pentane, from which a tan solid precipitated. Crystals suitable for single-crystal X-ray diffraction were obtained by slow diffusion of *n*-pentane onto a concentrated toluene solution. Yield: 0.055 g (53%). ^1^H NMR (600 MHz, C_6_D_6_): δ 6.94 (t, 1H, *J* = 6 Hz, aromatic H), 6.84 (t, 1H, *J* = 6 Hz, aromatic H), 6.63 (d, 1H, *J* = 6 Hz aromatic H), 6.48 (d, 1H, *J* = 6 Hz aromatic H), 3.69 (s, 3H, N–C*H*_3_), 3.23 (d, *J* = 6 Hz, 1H, N–C*H*_2_–P), 1.85 (m, 2H, P–C*H*(CH_3_)_2_), 1.04–0.98 (m, 6H, P–CH­(C*H*_3_)_2_), 0.74–0.68 (m, 6H, P–CH­(C*H*_3_)_2_). ^13^C­{^1^H} NMR (151 MHz, C_6_D_6_): δ 214.9 (d, *J* = 23 Hz, N*C*N), 214.5 (d, *J* = 18 Hz, Fe–*C*O), 214.2 (d, *J* = 17 Hz, Fe–*C*O), 213.5 (d, *J* = 18 Hz, Fe–*C*O), 137.3 (aromatic C), 133.8 (d, *J* = 11 Hz, aromatic C), 122.3 (aromatic CH), 122.0 (aromatic CH), 109.4 (aromatic CH), 109.1 (aromatic CH), 41.7 (d, *J* = 18 Hz, N–CH_2_–P), 34.55 (N–*C*H_3_), 26.0 (d, *J* = 23 Hz, P–*C*H­(CH_3_)_2_), 18.3 (P–CH­(*C*H_3_)_2_), 18.2 (P–CH­(*C*H_3_)_2_), 17.6 (P–CH­(*C*H_3_)_2_). ^31^P­{^1^H} NMR (243 MHz, C_6_D_6_): δ 110.6 (Fe–*P*). FTIR (cm^–1^): 2042 (CO), 2135 (CO), 2145 (CO).

#### [Fe­(PC-*i*Pr)­(CO)_3_H]­BF_4_ (**7**)

Inside an argon-filled glovebox, a microwave vial was charged with [(PCP-*i*Pr)­H]­PF_6_ (0.100 g, 0.256 mmol), K­[N­(SiMe_3_)_2_] (0.056 g, 0.282 mmol), [Fe_3_(CO)_12_] (0.047 g, 0.094 mmol) and THF (4 mL). The vial was capped, and the mixture was heated to 80 °C for 16 h. The resulting red-brown suspension was filtered, and all volatile materials were removed under vacuum. The residue was dissolved in diethyl ether (10 mL), HBF_4_·OEt_2_ (0.046 g, 0.282 mmol, 40 μL) was added and the mixture was stirred for 1 h, upon which a tan solid gradually precipitated. The supernatant was decanted off, the tan solid washed with diethyl ether and the solid dried under vacuum. Crystals suitable for single-crystal X-ray diffraction were obtained by slow diffusion of diethyl ether onto a concentrated acetonitrile solution. Yield: 0.083 g (66%). ^1^H NMR (600 MHz, CD_3_CN): δ 7.63–7.60 (m, 1H, aromatic H), 7.57–7.54 (m, 1H, aromatic H), 7.48–7.44 (m, 2H, aromatic H), 4.73 (dd, *J* = 18 and 12 Hz, 1H, N–C*H*_2_–P), 4.40 (dd, *J* = 12 and 2 Hz, 1H, N–C*H*_2_–P), 4.06 (s, 3H, N–C*H*_3_), 2.74–2.63 (m, 2H, P–C*H*(CH_3_)_2_), 1.49–1.39 (m, 6H, P–CH­(C*H*_3_)_2_), 1.19–1.13 (m, 3H, P–CH­(C*H*_3_)_2_), 0.81–0.76 (m, 3H, P–CH­(C*H*_3_)_2_), −8.92 (d, 1H, *J* = 48 Hz, Fe–*H*). ^13^C­{^1^H} NMR (151 MHz, CD_3_CN): δ 214.9 (d, *J* = 18 Hz, N*C*N), 207.6 (d, *J* = 24 Hz, Fe–*C*O), 206.8 (d, *J* = 32 Hz, Fe–*C*O), 205.9 (d, *J* = 9 Hz, Fe–*C*O), 190.6 (d, *J* = 20 Hz, aromatic C), 134.0 (d, *J* = 8 Hz, aromatic C), 125.6 (aromatic CH), 125.2 (aromatic CH), 112.3 (aromatic CH), 112.1 (aromatic CH), 43.2 (d, *J* = 36 Hz, N–*C*H_2_–P), 36.2 (N–*C*H_3_), 26.9 (d, *J* = 18 Hz, P–*C*H­(CH_3_)_2_), 25.3 (d, *J* = 32 Hz, N–*C*H_2_–P), 19.7 (P–CH­(*C*H_3_)_2_), 18.3 (P–CH­(*C*H_3_)_2_), 17.0 (P–CH­(*C*H_3_)_2_), 16.7 (P–CH­(*C*H_3_)_2_). ^11^B­{^1^H} NMR (193 MHz, CD_3_CN): δ −1.1 (*B*F_4_^–^). ^19^F­{^1^H} NMR (564 MHz, CD_3_CN): δ −151.2 (B*F*_4_^–^). ^31^P­{^1^H} NMR (243 MHz, CD_3_CN): δ 111.3 (Fe–*P*). FTIR (cm^–1^): 2015 (CO), 2071 (CO). HR-MS: *m*/*z* calcd for C_18_H_24_FeN_2_O_3_P [M + CO]^+^ 431.0818, found 431.0822.

#### [Co­(κ^2^-(P,C)-PCP-*i*Pr)­H­(CO)_2_] (**8**)

Inside an argon-filled glovebox, a screw cap vial was charged with complex **2** (0.050 g, 0.079 mmol), KO*t*Bu (0.018 g, 0.16 mmol), HBPin (0.050 g, 0.39 mmol) and THF (4 mL) and the mixture was stirred for 10 min. All volatile materials were evaporated to dryness under reduced pressure and the residue was extracted with diethyl ether, the extracts combined, concentrated and stored at −20 °C. Yield: 0.027 g (69%). ^1^H NMR (600 MHz, C_6_D_6_): δ 7.86 (m, 1H, aromatic H), 7.01 (m, 2H, aromatic H), 6.77 (m, 1H, aromatic H), 5.04 (d, 2H, *J* = 6 Hz, coordinated N–C*H*_2_–P), 3.37 (d, 2H, *J* = 6 Hz, free N–C*H*_2_–P), 2.03 (m, 2H, P–C*H*(CH_3_)_2_), 1.75 (m, 2H, P–C*H*(CH_3_)_2_), 1.10–0.98 (m, 26H, P–CH­(C*H*_3_)_2_), 0.79 (m, 6H, P–CH­(C*H*_3_)_2_), −12.29 (d, 1H, *J* = 54 Hz, Co–*H*). ^13^C­{^1^H} NMR (151 MHz, C_6_D_6_): δ 213.6 (br, N*C*N), 208.9 (br, Fe–*C*O), 136.9 (aromatic C), 134.6 (d, *J* = 9 Hz, aromatic C), 122.3 (aromatic CH), 121.5 (aromatic CH) 113.4 (d, *J* = 15 Hz, aromatic CH), 109.4 (aromatic CH), 47.1 (t, *J* = 21 Hz, coordinated N–*C*H_2_–P), 42.9 (t, *J* = 14 Hz, free P–*C*H­(CH_3_)_2_), 26.1 (d, *J* = 15 Hz, P–CH­(*C*H_3_)_2_), 23.3 (d, *J* = 14 Hz, P–CH­(*C*H_3_)_2_), 19.6 (m, P–CH­(*C*H_3_)_2_), 18.3 (d, *J* = 6 Hz, P–CH­(*C*H_3_)_2_), 17.9 (P–CH­(*C*H_3_)_2_). ^31^P­{^1^H} NMR (243 MHz, C_6_D_6_): δ 100.4 (coordinated P), −4.5 (free P).

#### General Procedure for the Hydroboration of Alkenes

Inside an argon-filled glovebox, a screw cap vial (8 mL) was charged with a magnetic stirring-bar, desired alkene (0.5 mmol, 1.0 equiv) and pinacolborane (0.55 mmol, 1.1 equiv). To this mixture, 0.5 mL of a stock solution of the desired complex (1 mol %) and KO*t*Bu (2 mol %) in THF was added. The vial was closed, transferred out of the glovebox and stirred for 16 h at 70 °C. Afterward, the reaction mixture was cooled to room temperature and exposed to air. The mixture was filtered through a thin pad of silica eluting with diethyl ether, all volatile materials carefully removed and the resulting product dried in vacuum.

## Supplementary Material



## Notes

NMR spectra of all compounds (PDF)
