# Supplementary material for: Synthesis, Characterization and Catalytic Activity of Iron, Cobalt and Nickel Complexes Bearing an N‑Heterocyclic Carbene-Based PCP Pincer Ligand
Source: Organometallics. 2025 May 28;44(11):1217–26. doi: 10.1021/acs.organomet.5c00172 (PMC12152947; doi:10.1021/acs.organomet.5c00172)
Supplement: Supplementary file 1 [file om5c00172_si_001.pdf]

Supporting Information for

Synthesis, Characterization and Catalytic Activity of Iron, Cobalt and Nickel Complexes Bearing an N-Heterocyclic Carbene-Based PCP Pincer Ligand

Tiago F. C. Cruz,[a,b] Daniel P. Zobernig,[a] Berthold Stöger,[c] Ernst Pittenauer[d] and Karl Kirchner[a]*

[a] Institute of Applied Synthetic Chemistry, TU Wien, Getreidemarkt 9/163-AC, A-1060 Wien, Austria.

[b] Centro de Química Estrutural, Institute of Molecular Sciences, Departamento de Engenharia Química, Instituto Superior Técnico, Universidade de Lisboa, Av. Rovisco Pais, 1049 001 Lisboa, Portugal

[c] X-Ray Center, TU Wien, Getreidemarkt 9/163, A-1060 Wien, Austria.

[d] Institute of Chemical Technologies and Analytics, TU Wien, Getreidemarkt 9, A-1060 Vienna, Austria

*Corresponding Author; e-mail: karl.kirchner@tuwien.ac.at

Table of contents

Table of contents S1

Characterization data for all complexes S2

Characterization data for the products isolated from the catalytic runs S18

Characterization data for all complexes

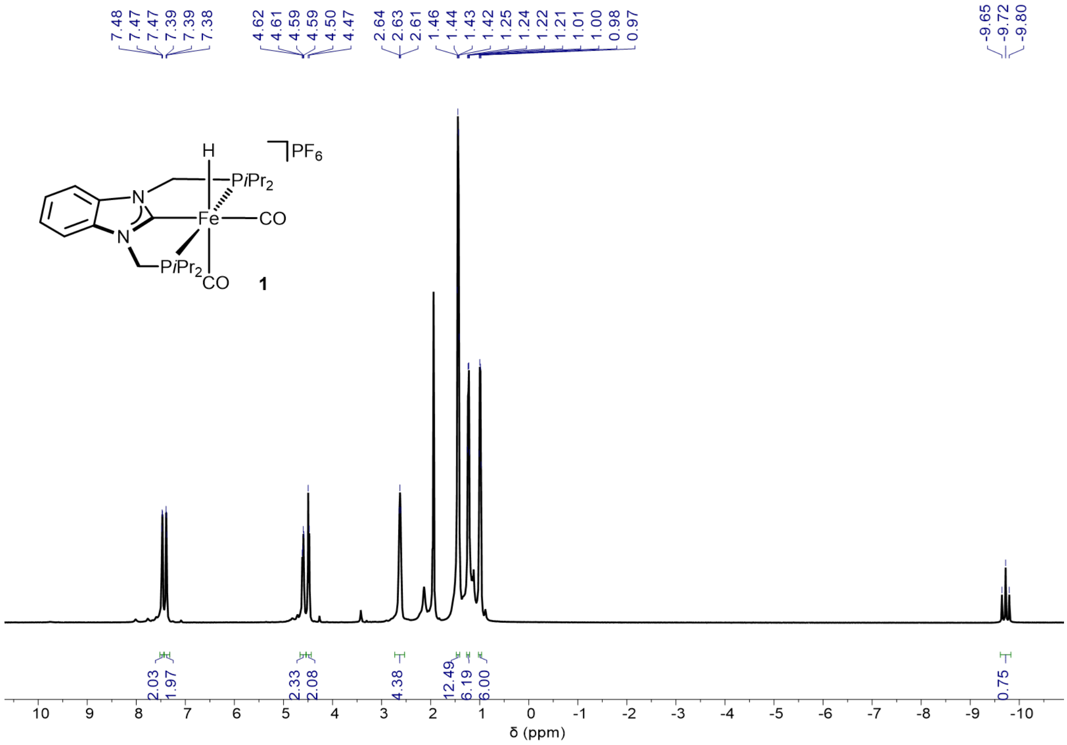

Figure S1 ^{1}H NMR spectrum (600 MHz, CD_3CN) of complex **[Fe(PCP-*i*Pr)H(CO)$_2$]PF$_6$** (**1**).

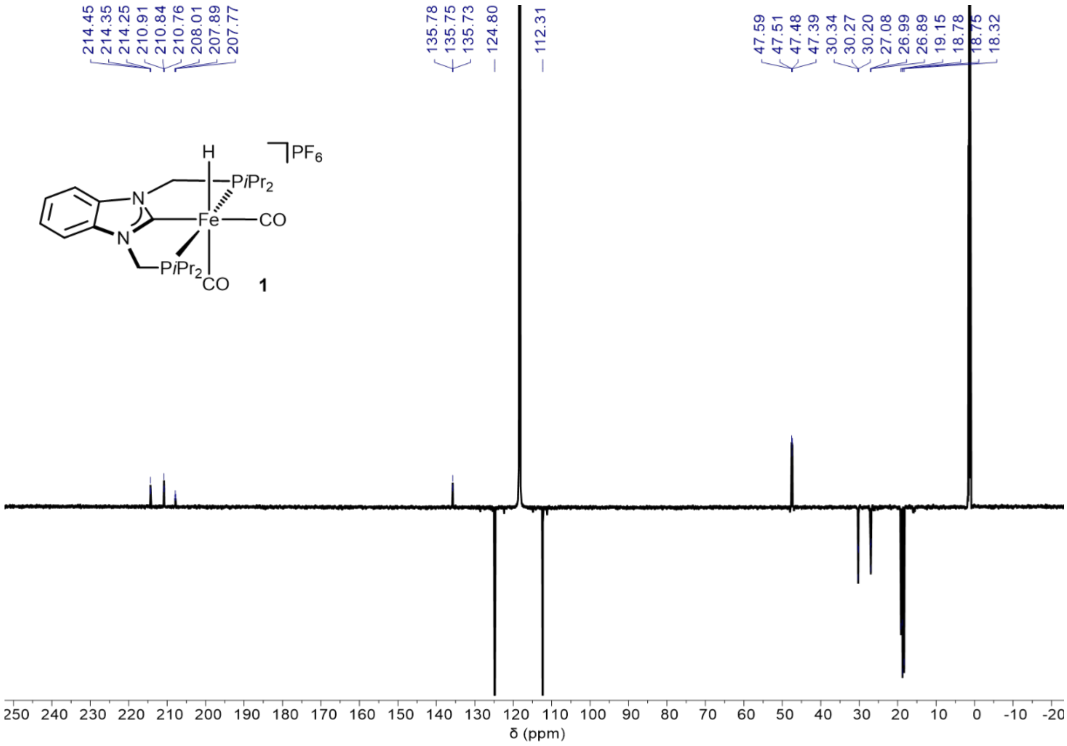

Figure S2 ^{13}C (APT) NMR spectrum (151 MHz, CD_3CN) of complex **[Fe(PCP-*i*Pr)H(CO)$_2$]PF$_6$** (**1**).

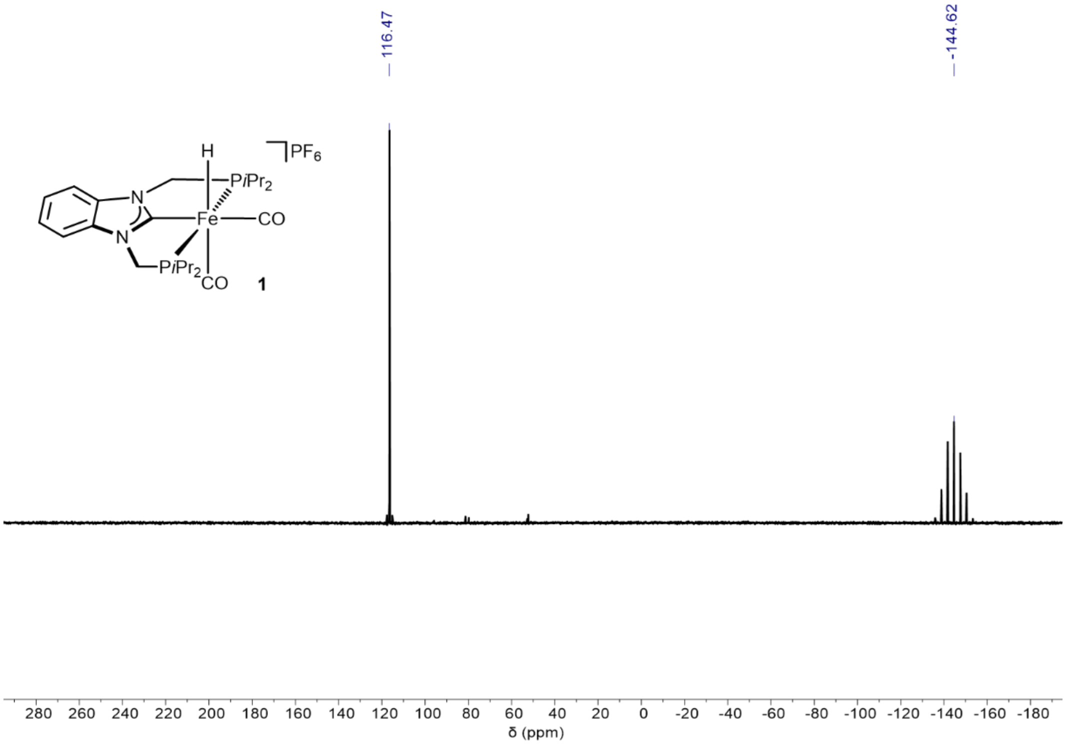

Figure S3 $^{31}P\{^{1}H\}$ NMR spectrum (243 MHz, CD_3CN) of complex **[Fe(PCP-*i*Pr)H(CO)$_2$]PF$_6$** (**1**).

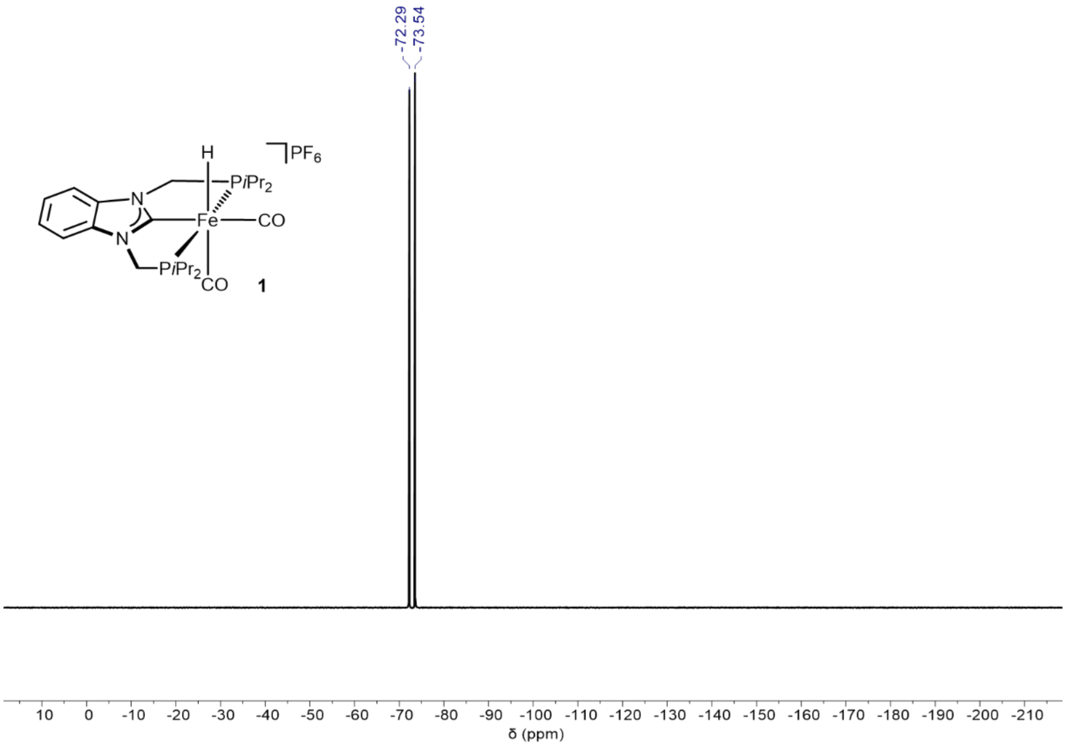

Figure S4 $^{19}F\{^{1}H\}$ NMR spectrum (564 MHz, CD_3CN) of complex **[Fe(PCP-*i*Pr)H(CO)$_2$]PF$_6$** (**1**).

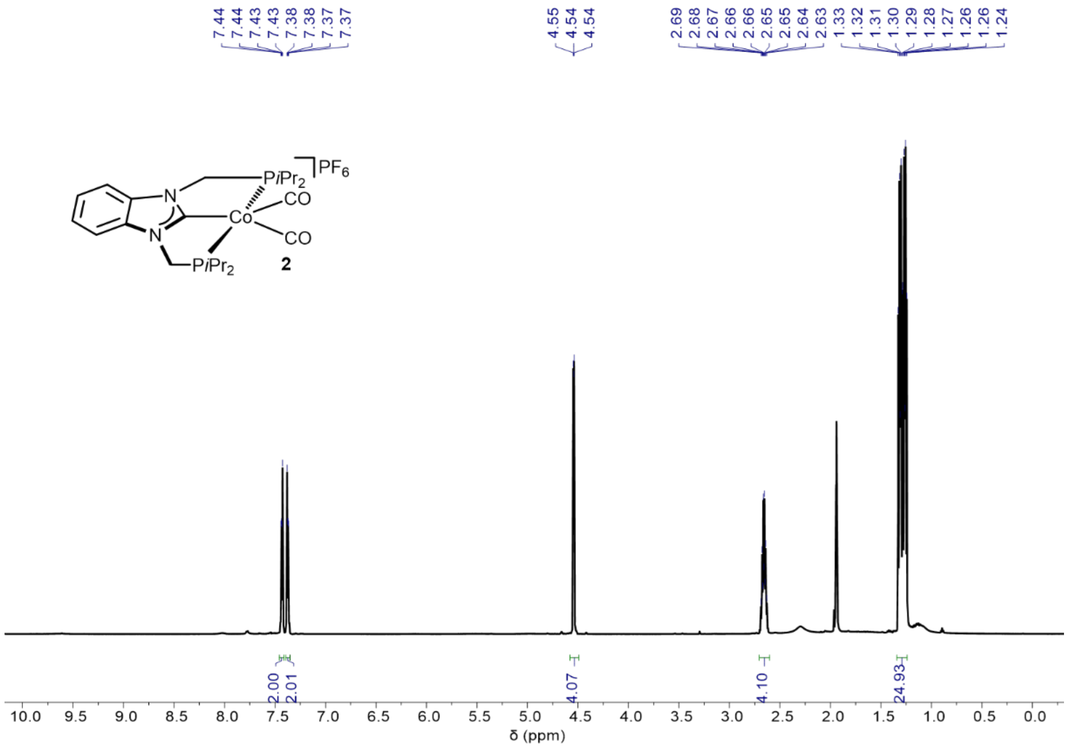

Figure S5 ^{1}H NMR spectrum (600 MHz, CD_3CN) of complex **[Co(PCP-*i*Pr)(CO)$_2$]PF$_6$** (**2**).

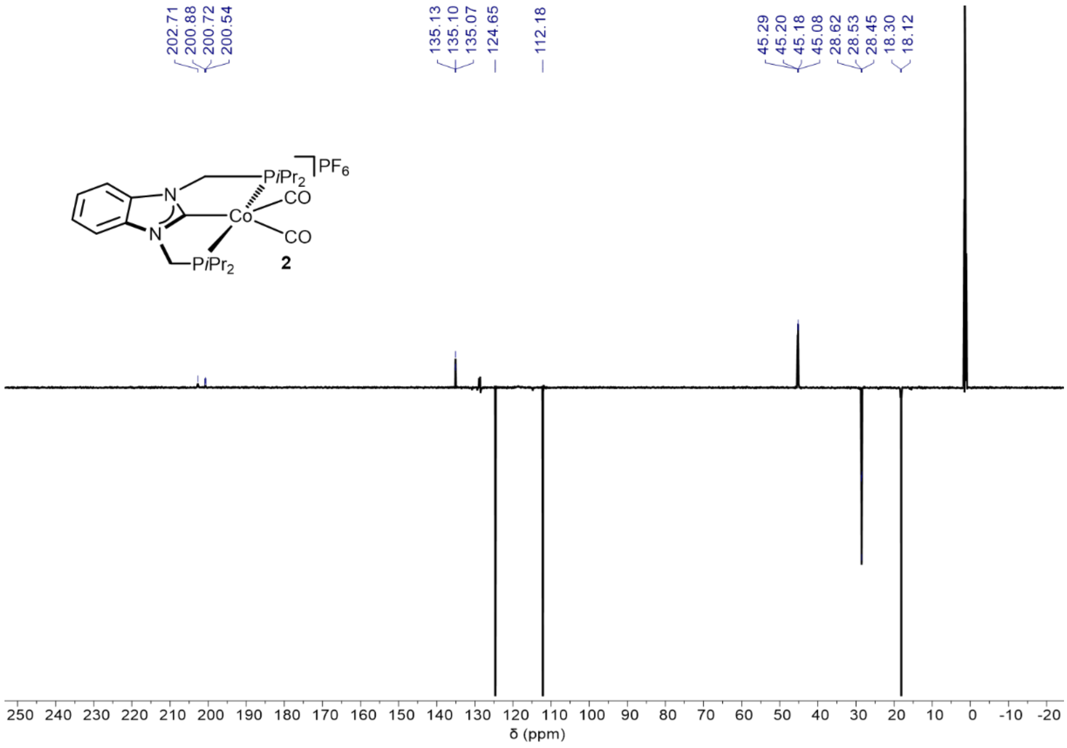

Figure S6 ^{13}C (APT) NMR spectrum (151 MHz, CD_3CN) of complex **[Co(PCP-*i*Pr)(CO)$_2$]PF$_6$** (**2**).

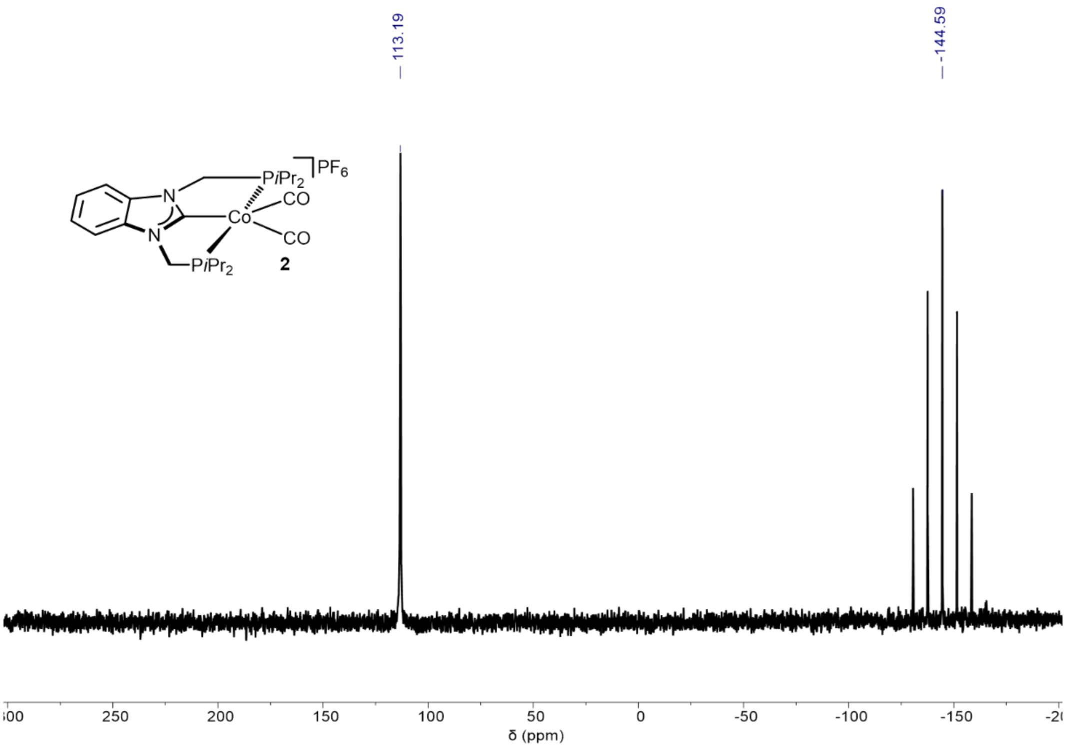

Figure S7 $^{31}P\{^1H\}$ NMR spectrum (243 MHz, CD_3CN) of complex **[Co(PCP-*i*Pr)(CO)$_2$]PF$_6$** (**2**).

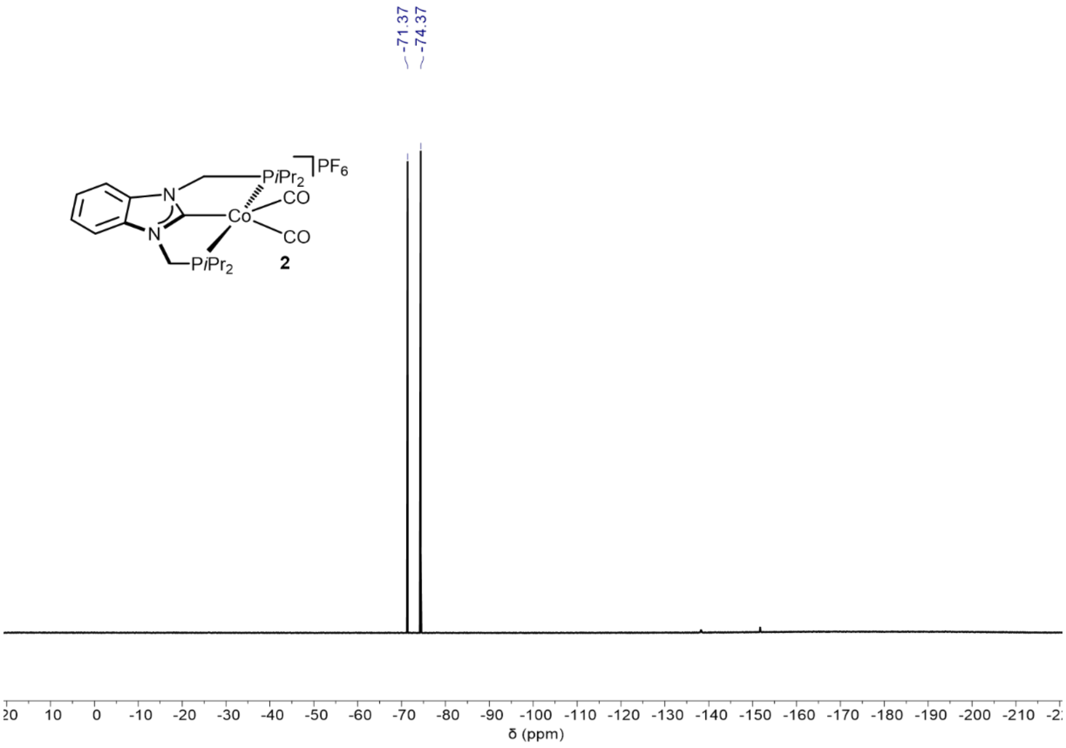

Figure S8 $^{19}F\{^1H\}$ NMR spectrum (564 MHz, CD_3CN) of complex **[Co(PCP-*i*Pr)(CO)$_2$]PF$_6$** (**2**).

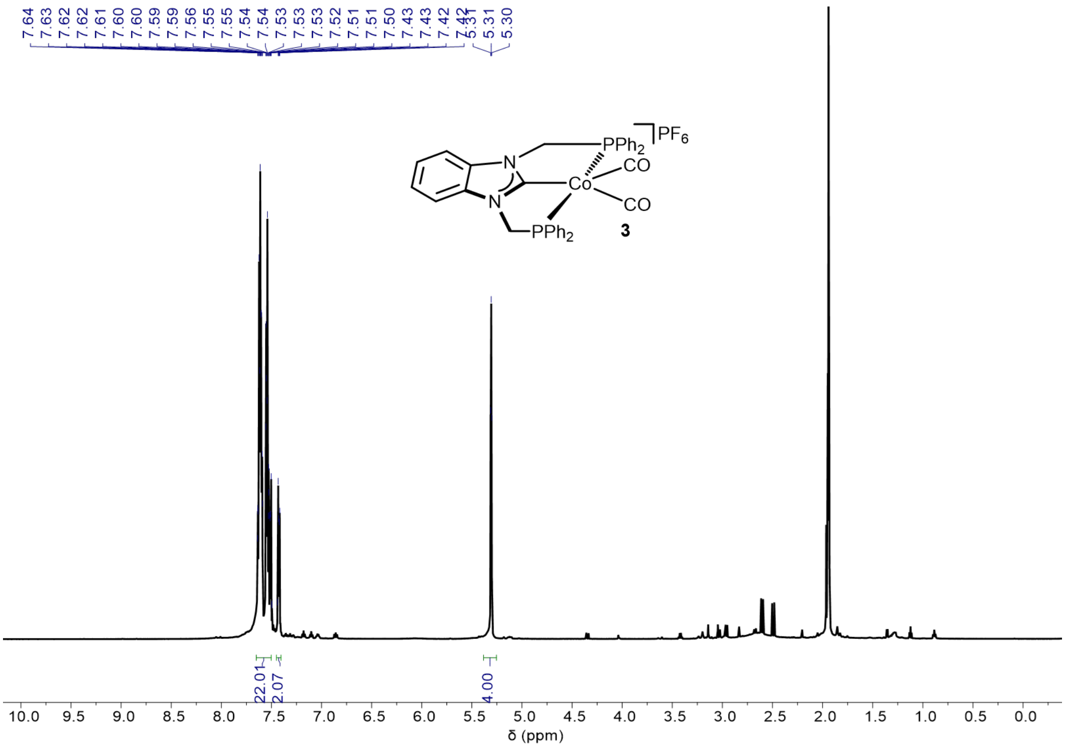

Figure S9 ^{1}H NMR spectrum (600 MHz, CD_3CN) of complex **[Co(PCP-Ph)(CO)$_2$]PF$_6$ (3)**.

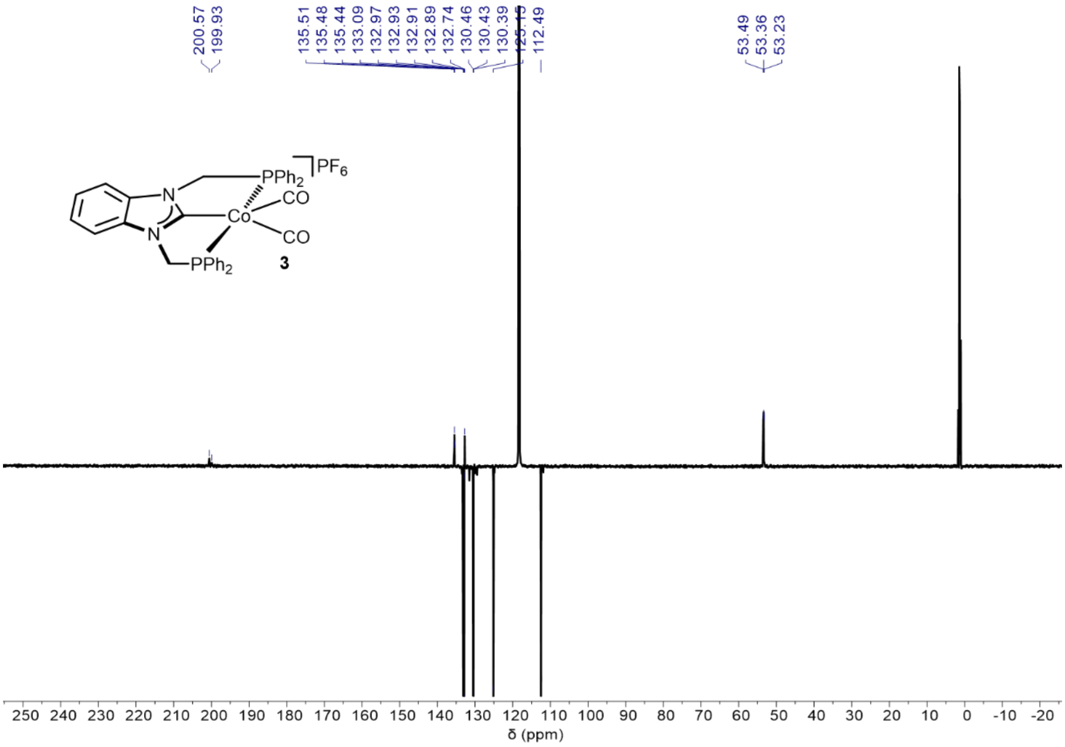

Figure S10 ^{13}C (APT) NMR spectrum (151 MHz, CD_3CN) of complex **[Co(PCP-Ph)(CO)$_2$]PF$_6$ (3)**.

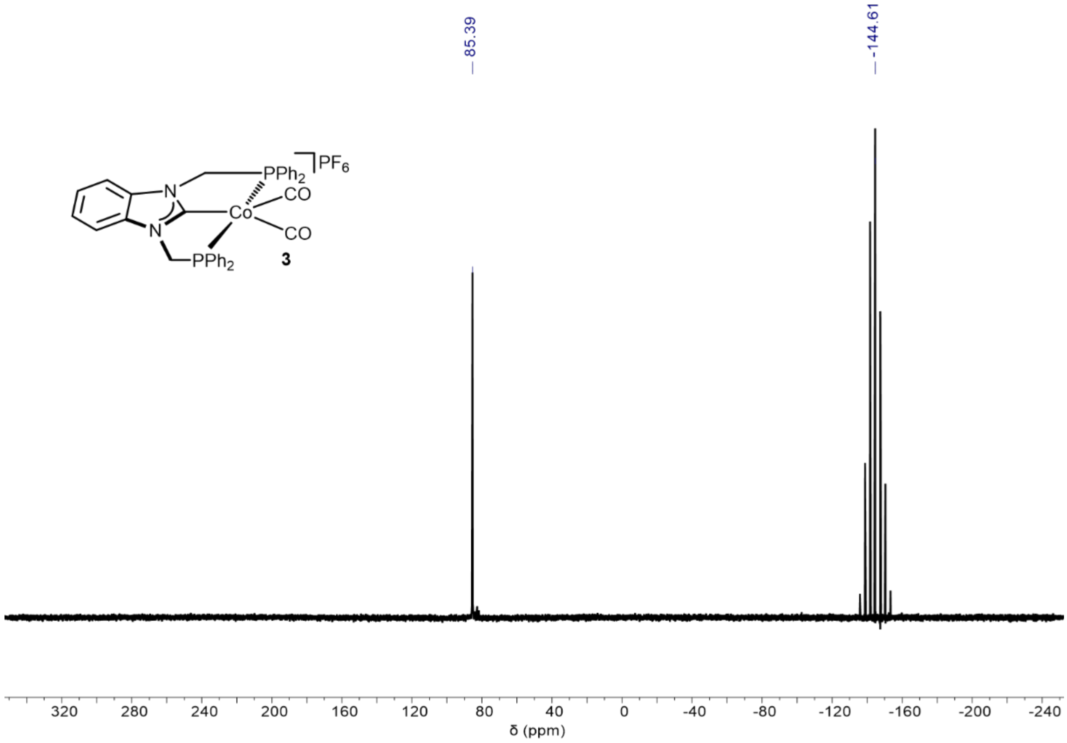

Figure S11$^{31}P\{^{1}H\}$ NMR spectrum (243 MHz, CD_3CN) of complex **$[Co(PCP\text{-}Ph)(CO)_2]PF_6$** (**3**).

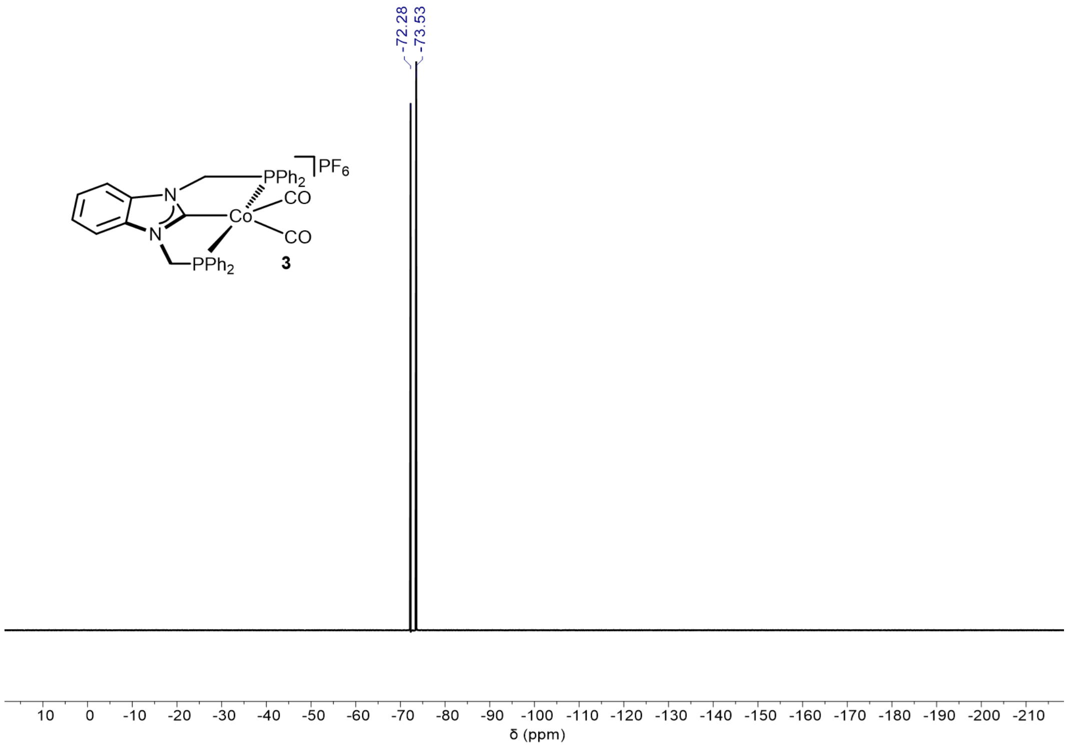

Figure S12 $^{19}F\{^{1}H\}$ NMR spectrum (564 MHz, CD_3CN) of complex **$[Co(PCP\text{-}Ph)(CO)_2]PF_6$** (**4**).

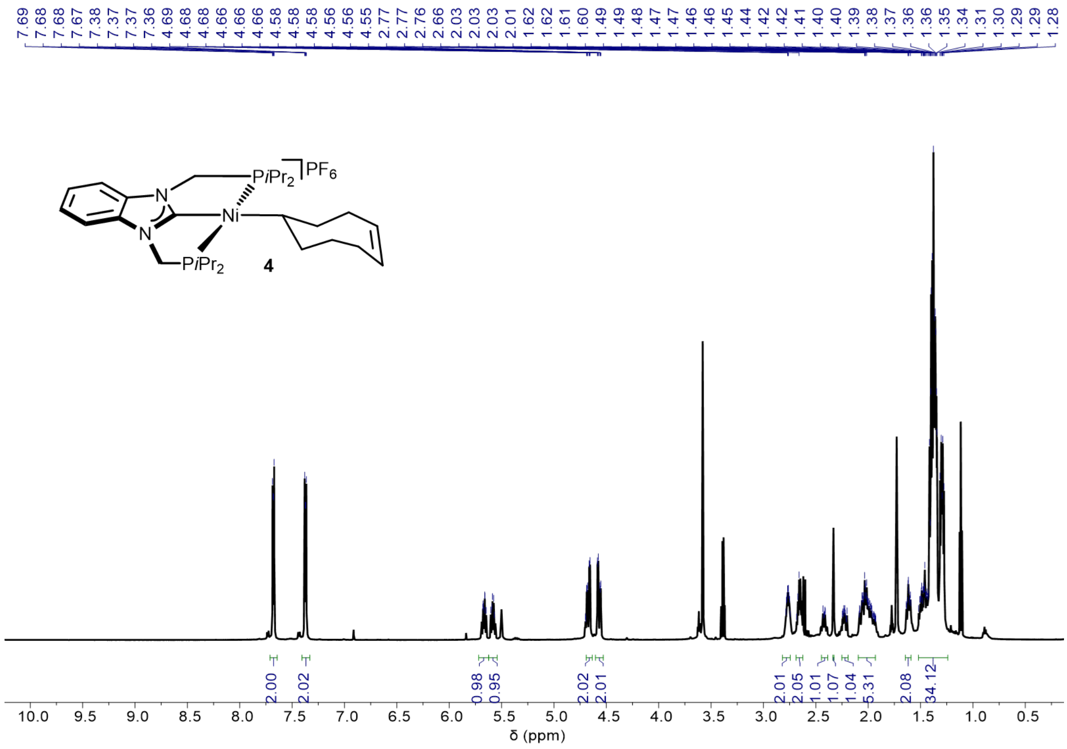

Figure S13 ^{1}H NMR spectrum (600 MHz, THF-d_8) of complex **[Ni(PCP-*i*Pr)(cycloot-4-en-1-yl)]PF$_6$** (**4**).

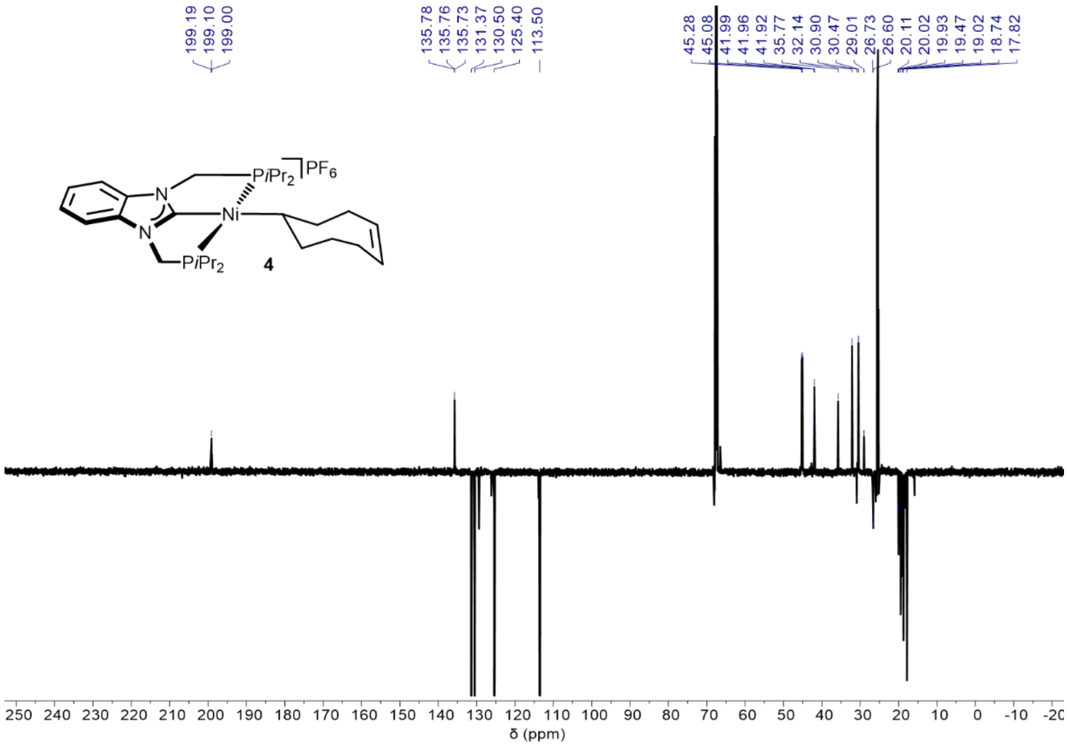

Figure S14 ^{13}C (APT) NMR spectrum (151 MHz, THF-d_8) of complex **[Ni(PCP-*i*Pr)(cycloot-4-en-1-yl)]PF$_6$** (**4**).

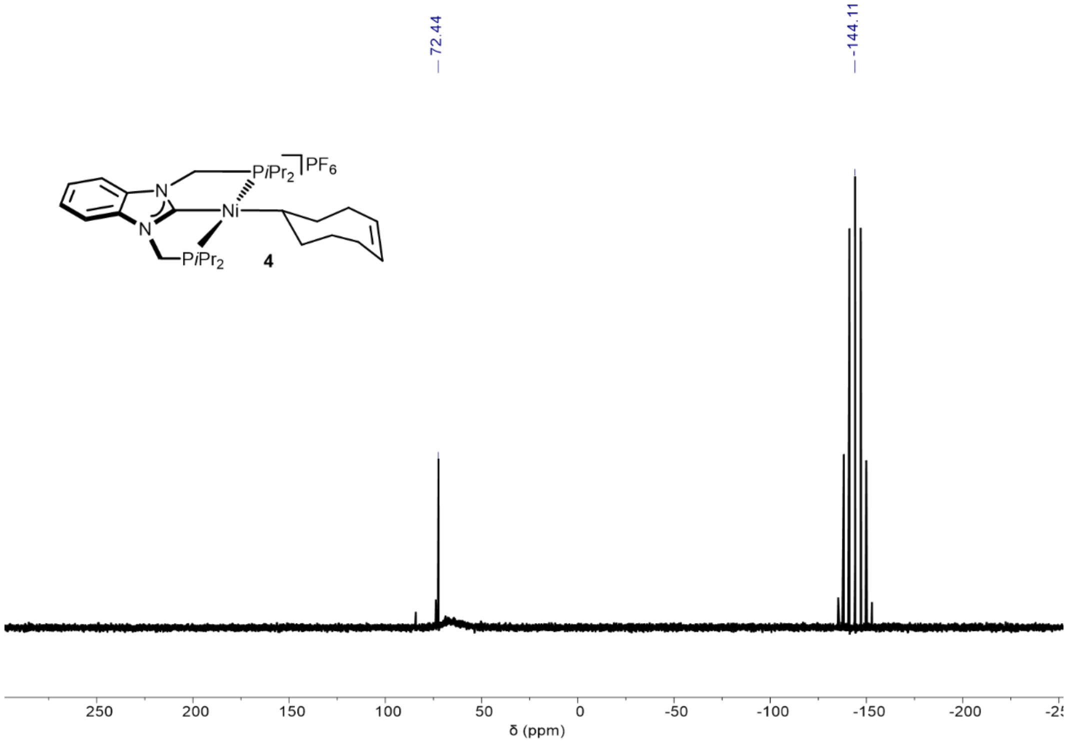

Figure S15 $^{31}P\{^{1}H\}$ NMR spectrum (243 MHz, THF-d_8) of complex **[Ni(PCP-*i*Pr)(cycloot-4-en-1-yl)]PF$_6$** (**4**).

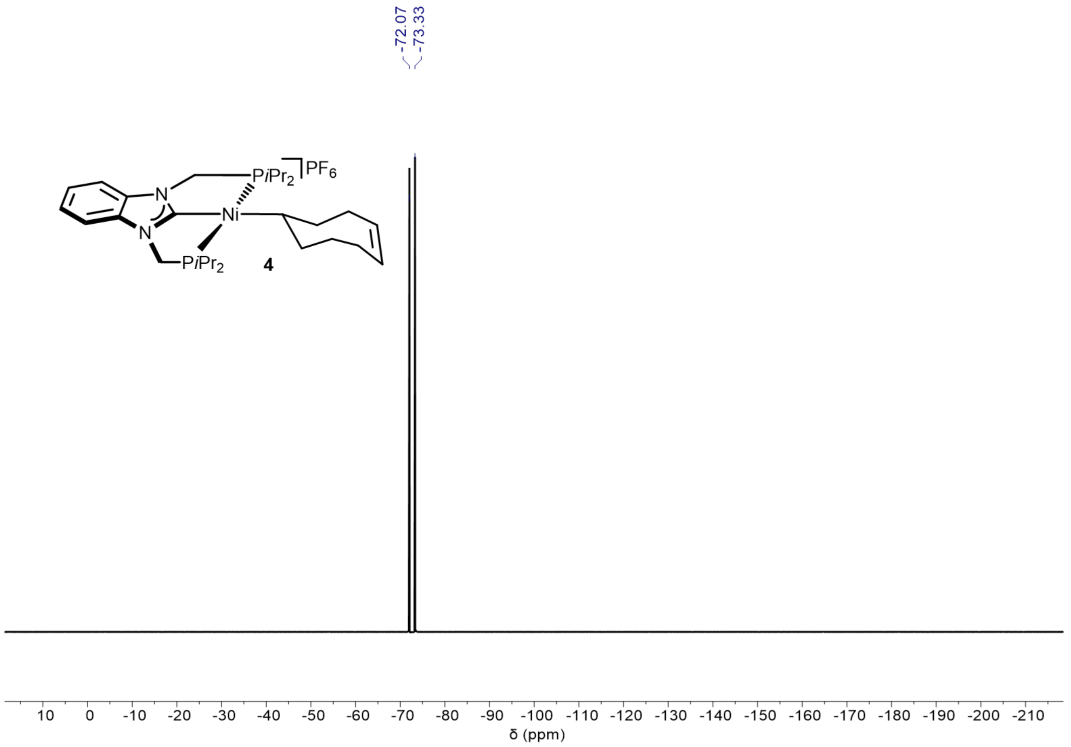

Figure S16 $^{19}F\{^{1}H\}$ NMR spectrum (564 MHz, THF-d_8) of complex **[Ni(PCP-*i*Pr)(cycloot-4-en-1-yl)]PF$_6$** (**4**).

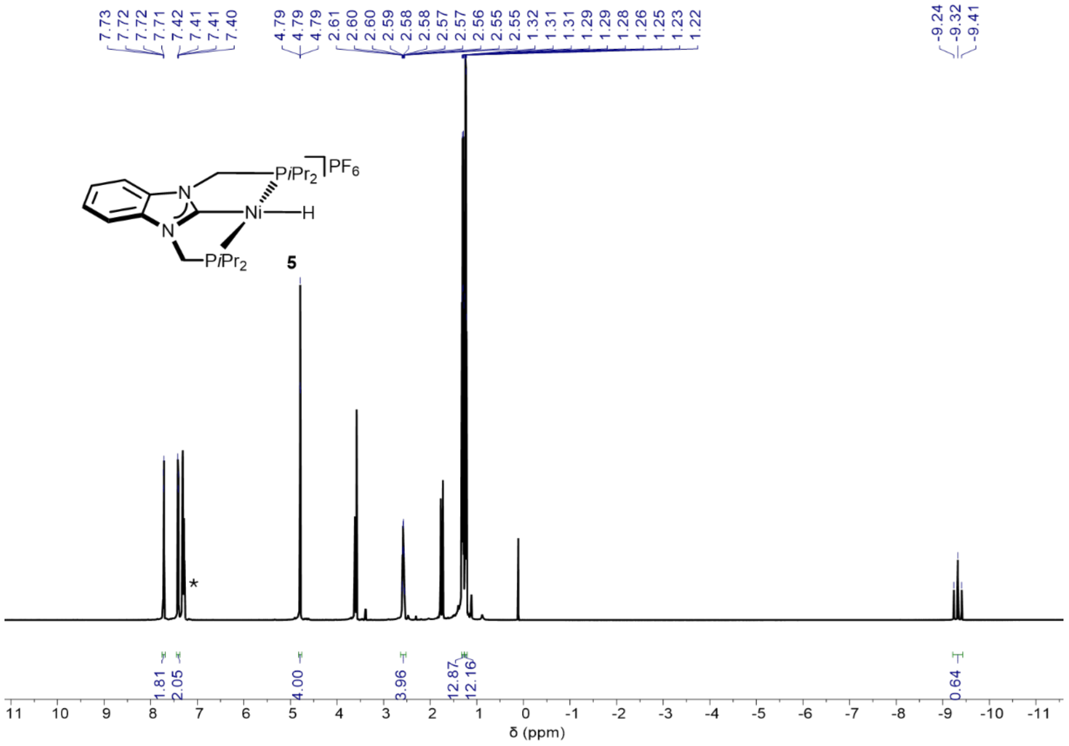

Figure S17 ^{1}H NMR spectrum (600 MHz, THF-d_8) of complex **[Ni(PCP-*i*Pr)H]PF$_6$ 5**. The asterisk denotes free PPh_3.

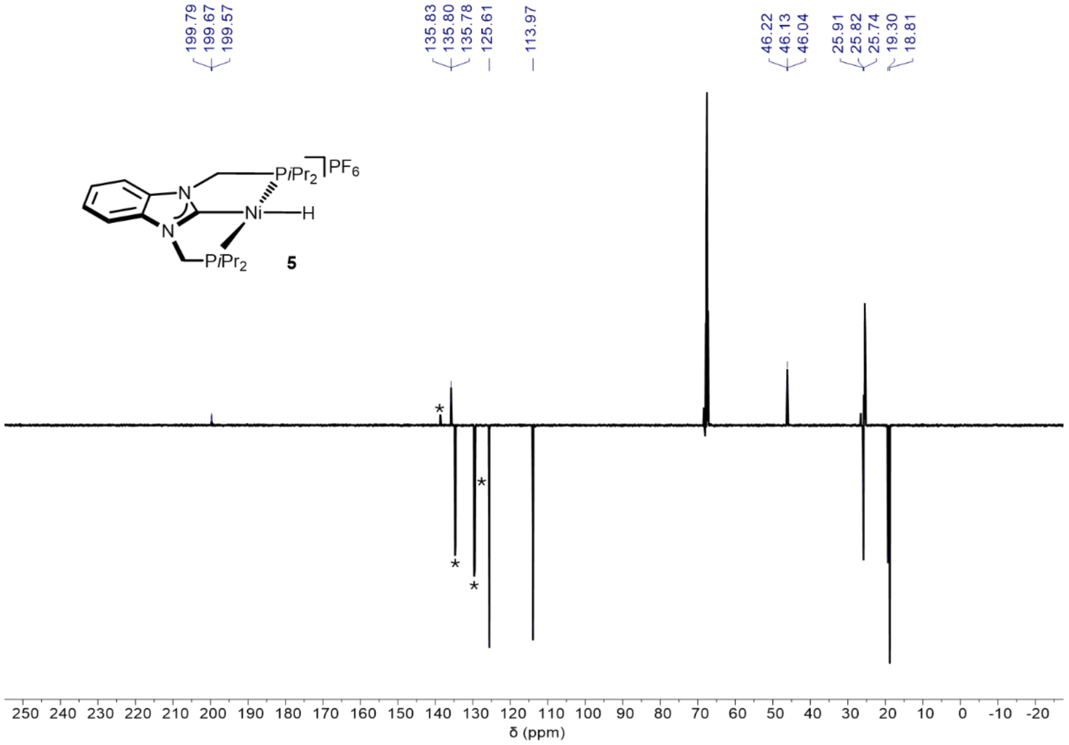

Figure S18 ^{13}C (APT) NMR spectrum (151 MHz, THF-d_8) of complex **[Ni(PCP-*i*Pr)H]PF$_6$** (**5**). The asterisks denote free PPh_3.

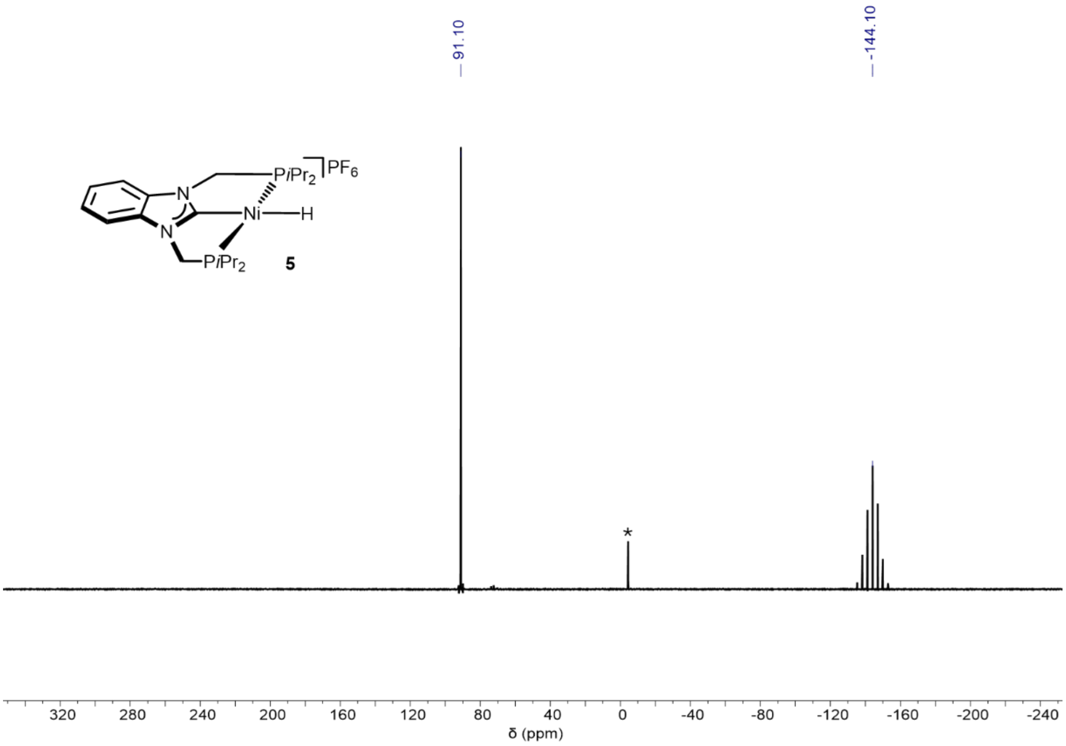

Figure S19 $^{31}P\{^{1}H\}$ NMR spectrum (243 MHz, THF-*d*8) of complex **[Ni(PCP-*i*Pr)H]PF6** (**5**). The asterisk denotes free PPh_3.

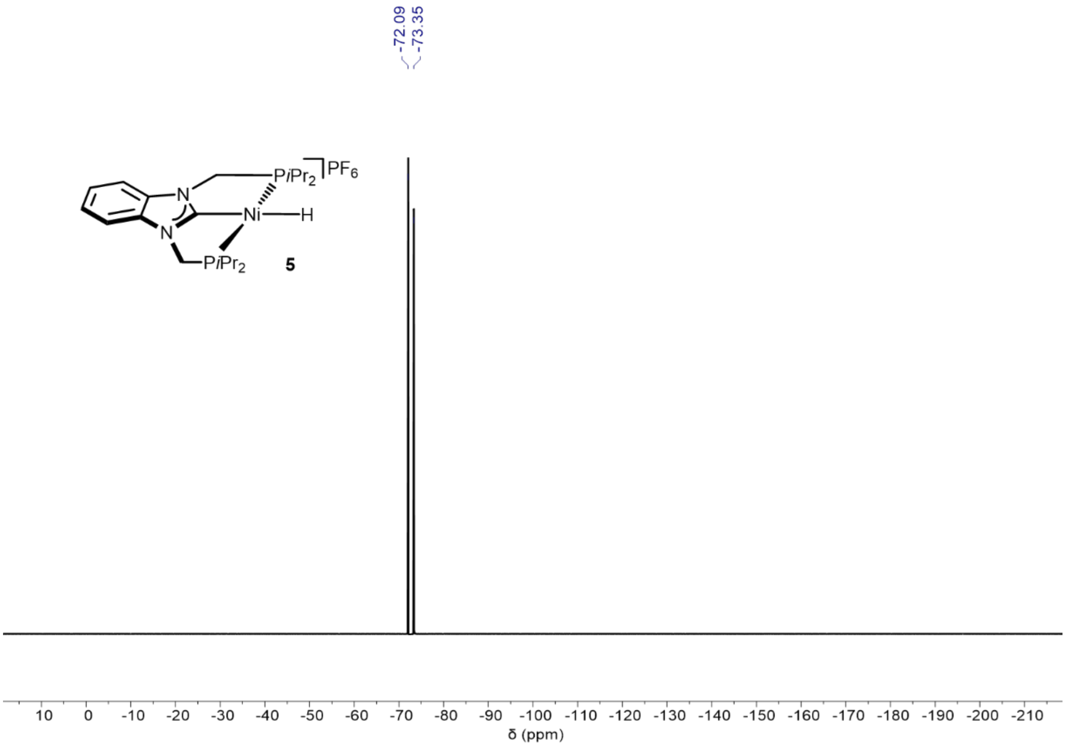

Figure S20 $^{19}F\{^{1}H\}$ NMR spectrum (564 MHz, THF-*d*8) of complex **[Ni(PCP-*i*Pr)H]PF6** (**5**).

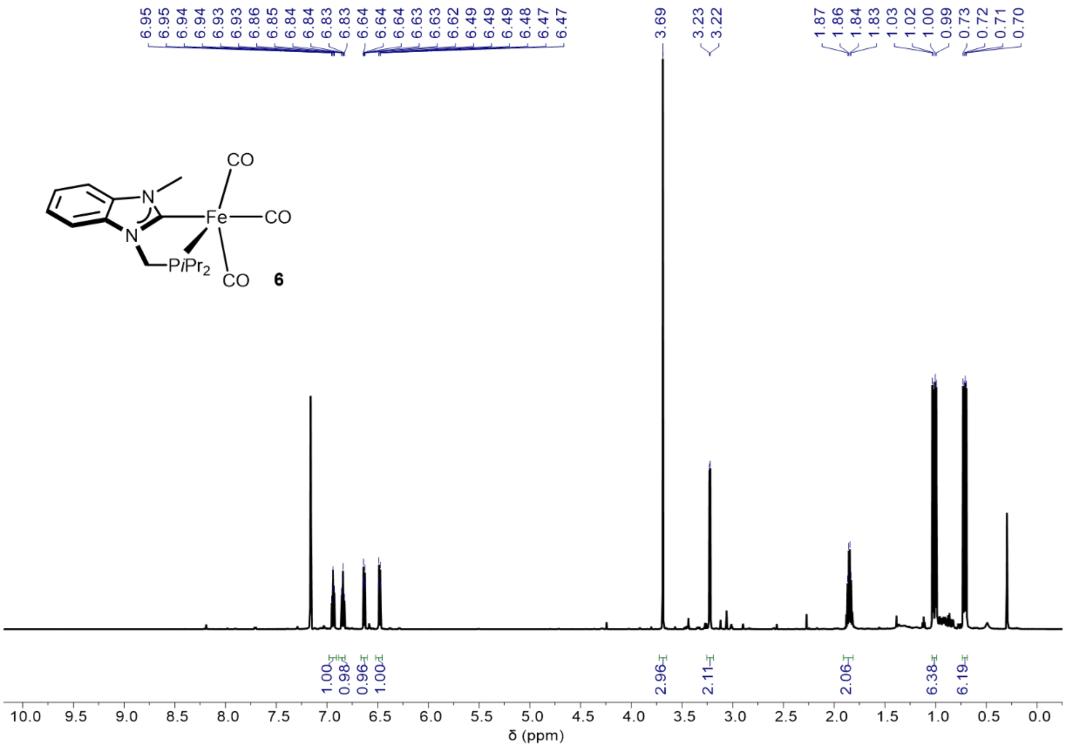

Figure S21 ^{1}H NMR spectrum (600 MHz, C_6D_6) of complex **[Fe(PC-*i*Pr)(CO)$_3$]PF$_6$** (**6**).

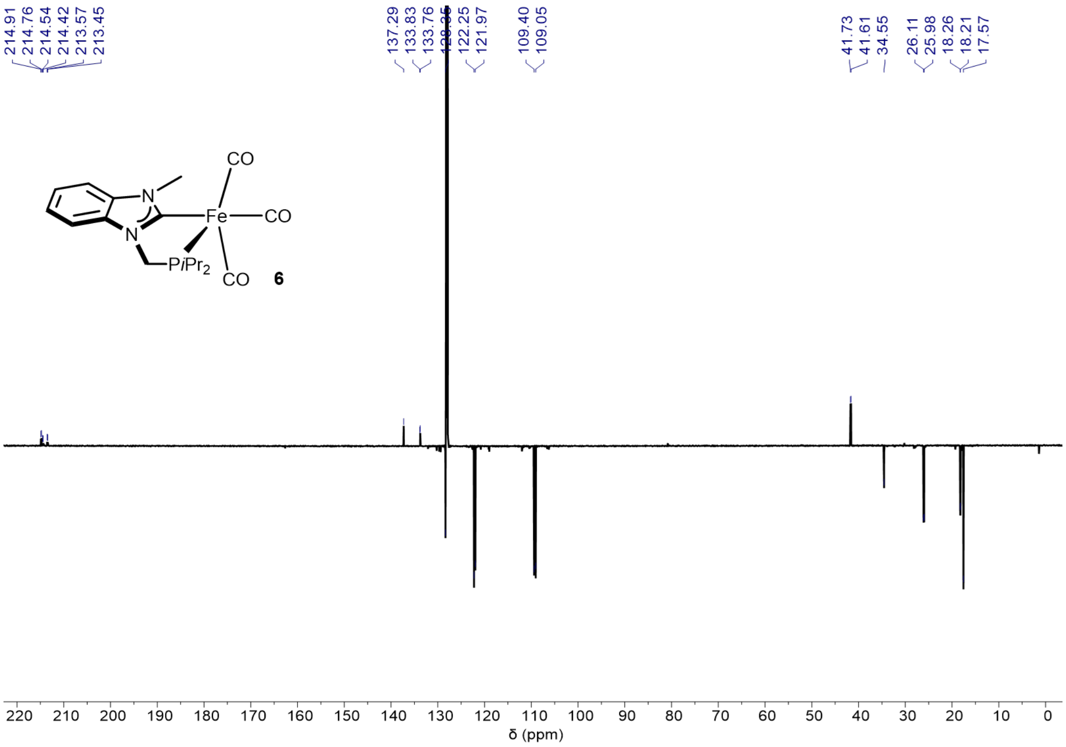

Figure S22 ^{13}C (APT) NMR spectrum (151 MHz, C_6D_6) of complex **[Fe(PC-*i*Pr)(CO)$_3$]PF$_6$** (**6**).

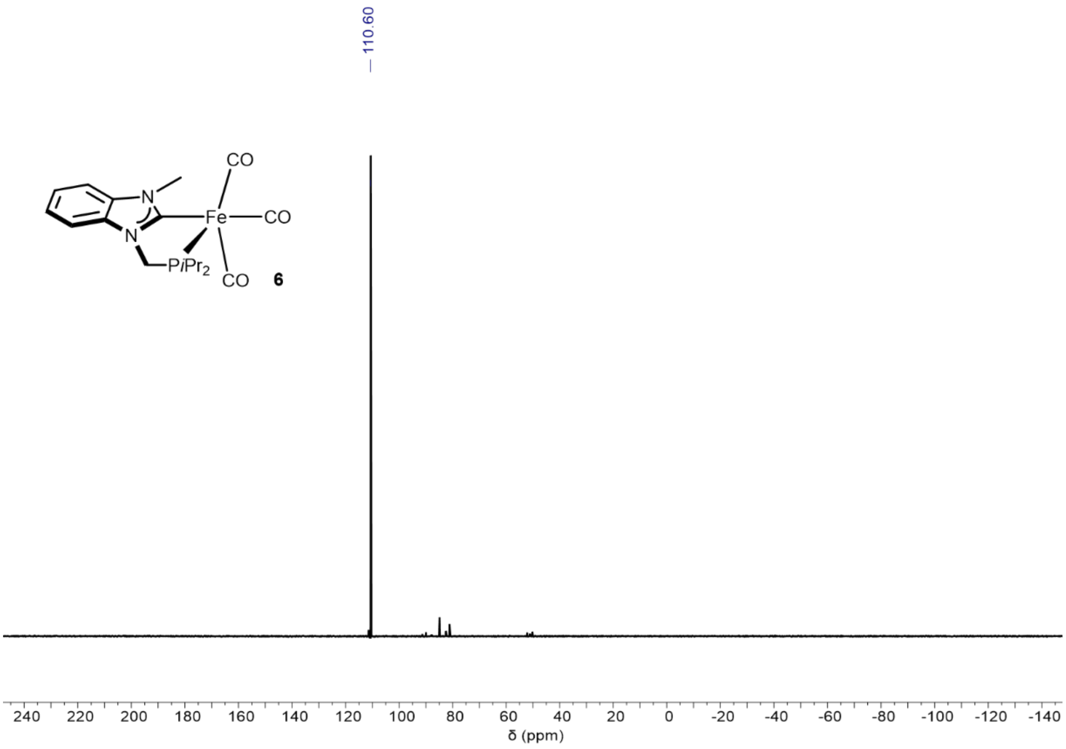

Figure S23^{31}P{^{1}H} NMR spectrum (243 MHz, C_6D_6) of complex **[Fe(PC-*i*Pr)(CO)$_3$]PF$_6$ (6)**.

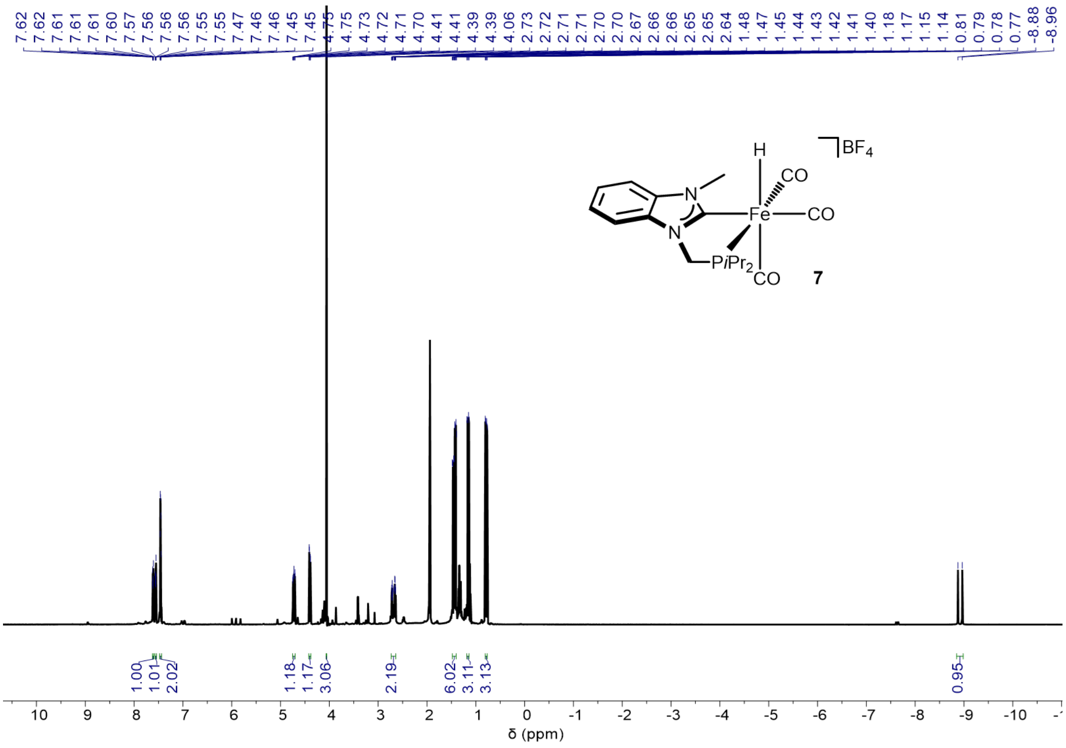

Figure S24 ^{1}H NMR spectrum (600 MHz, CD_3CN) of complex **[Fe(PC-*i*Pr)H(CO)$_3$]BF$_4$ (7)**.

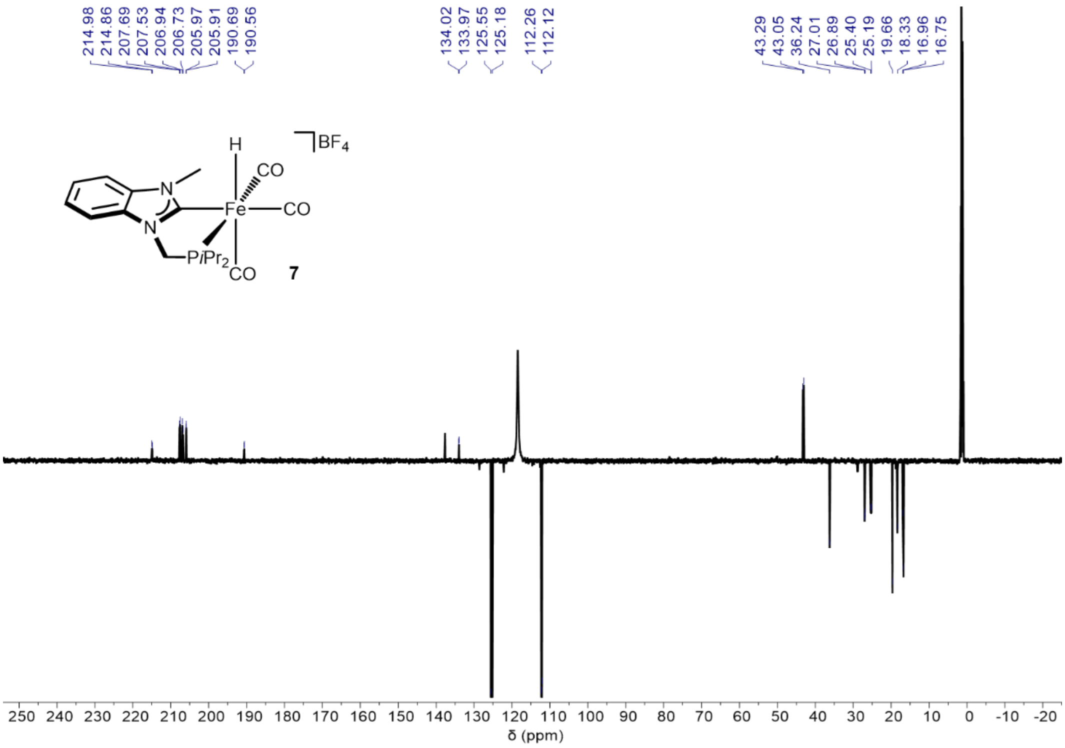

Figure S25 ^{13}C (APT) NMR spectrum (151 MHz, CD_3CN) of complex **[Fe(PC-*i*Pr)H(CO)$_3$]BF$_4$** (**7**).

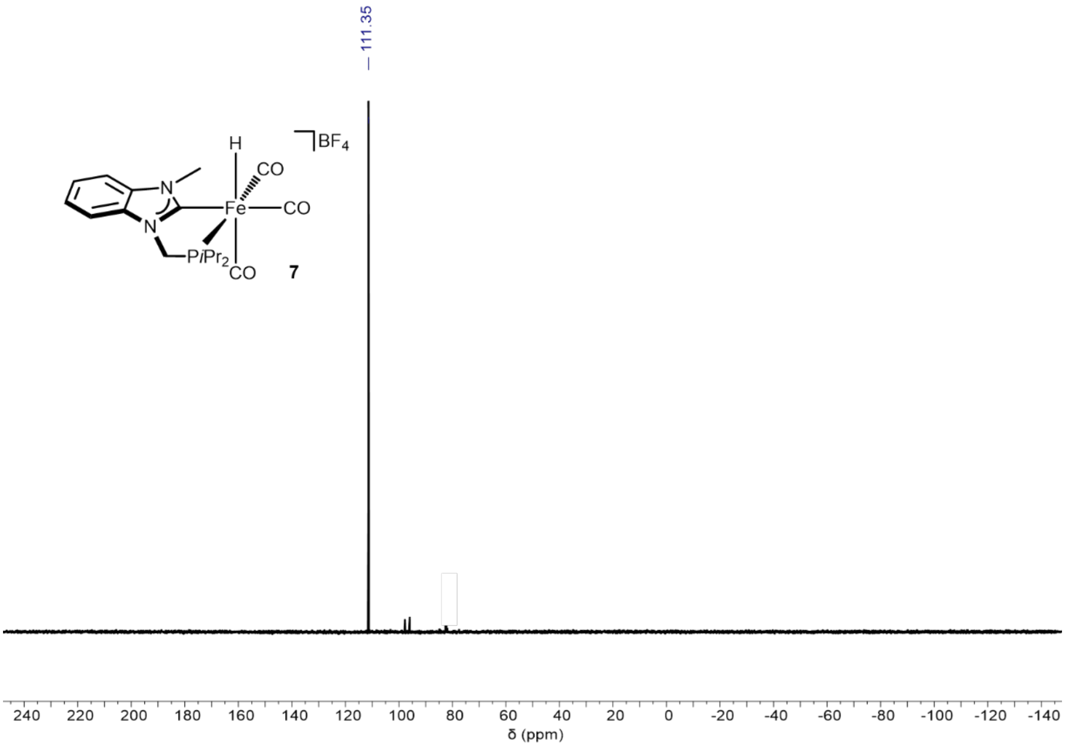

Figure S26 ^{31}P{^{1}H} NMR spectrum (193 MHz, CD_3CN) of complex **[Fe(PC-*i*Pr)H(CO)$_3$]BF$_4$** (**7**).

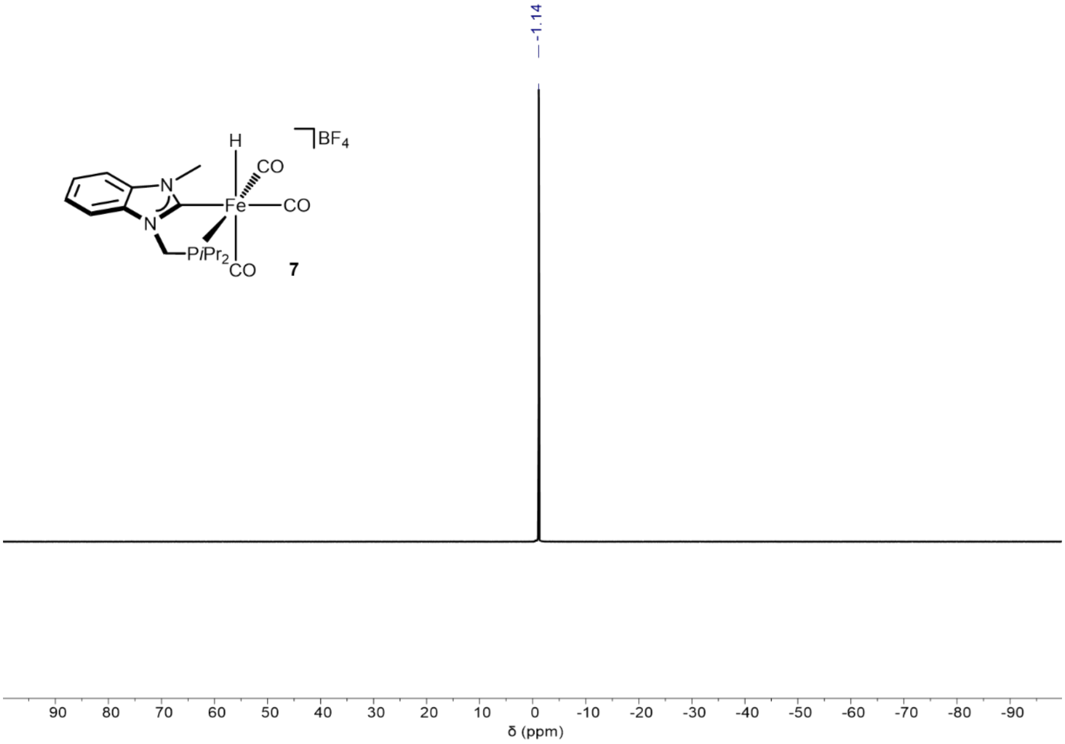

Figure S27^{11}B{^{1}H} NMR spectrum (243 MHz, CD_3CN) of complex **[Fe(PC-*i*Pr)H(CO)$_3$]BF$_4$** (**7**).

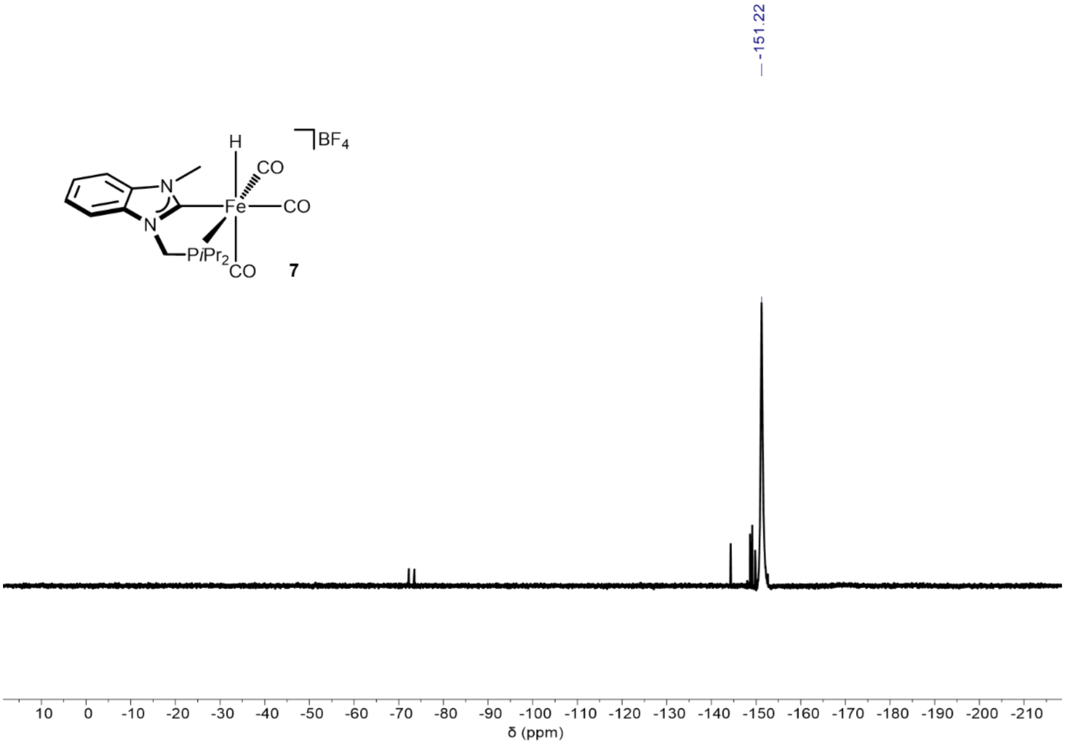

Figure S28 ^{19}F{^{1}H} NMR spectrum (564 MHz, CD_3CN) of complex **[Fe(PC-*i*Pr)H(CO)$_3$]BF$_4$** (**7**).

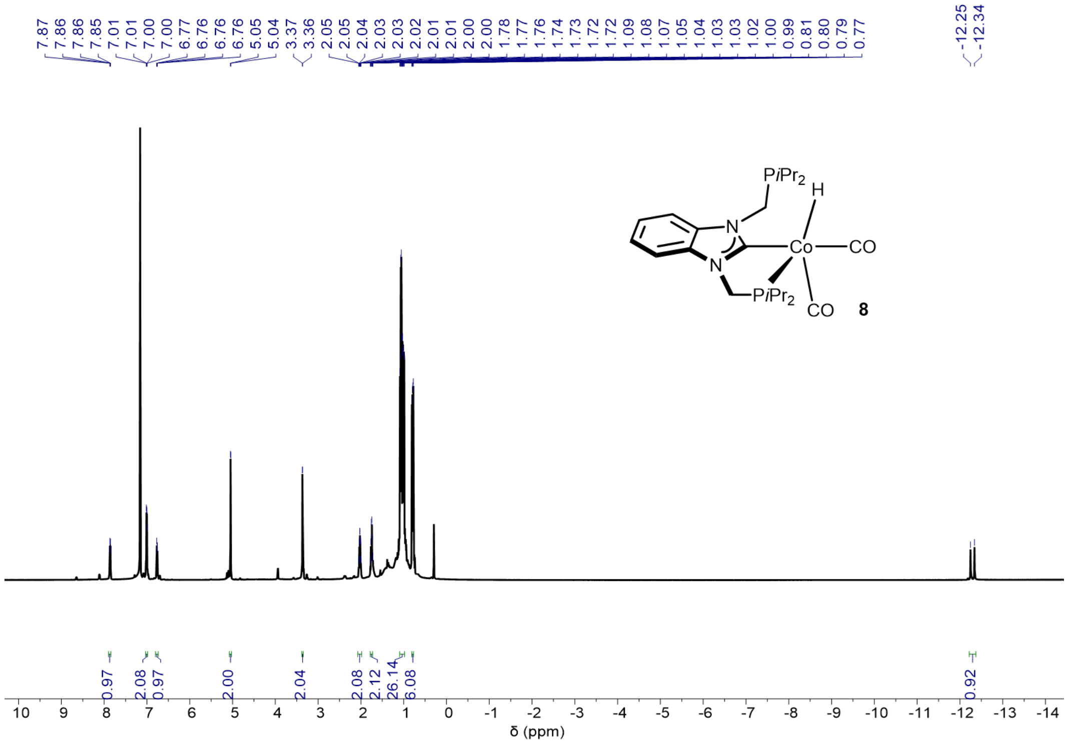

Figure S29 ^{1}H NMR spectrum (600 MHz, C_6D_6) of complex **[Co(PCP-*i*Pr)H(CO)$_2$]** (**8**).

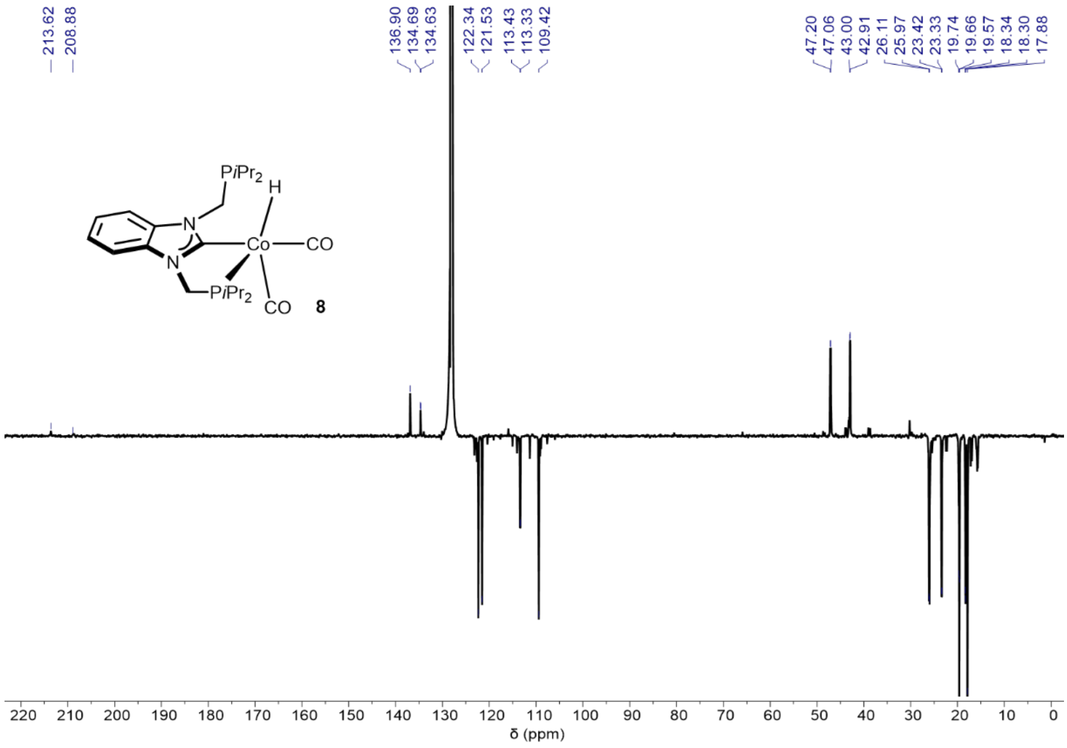

Figure S30 ^{13}C (APT) NMR spectrum (151 MHz, C_6D_6) of complex **[Co(PCP-*i*Pr)H(CO)$_2$]** (**8**).

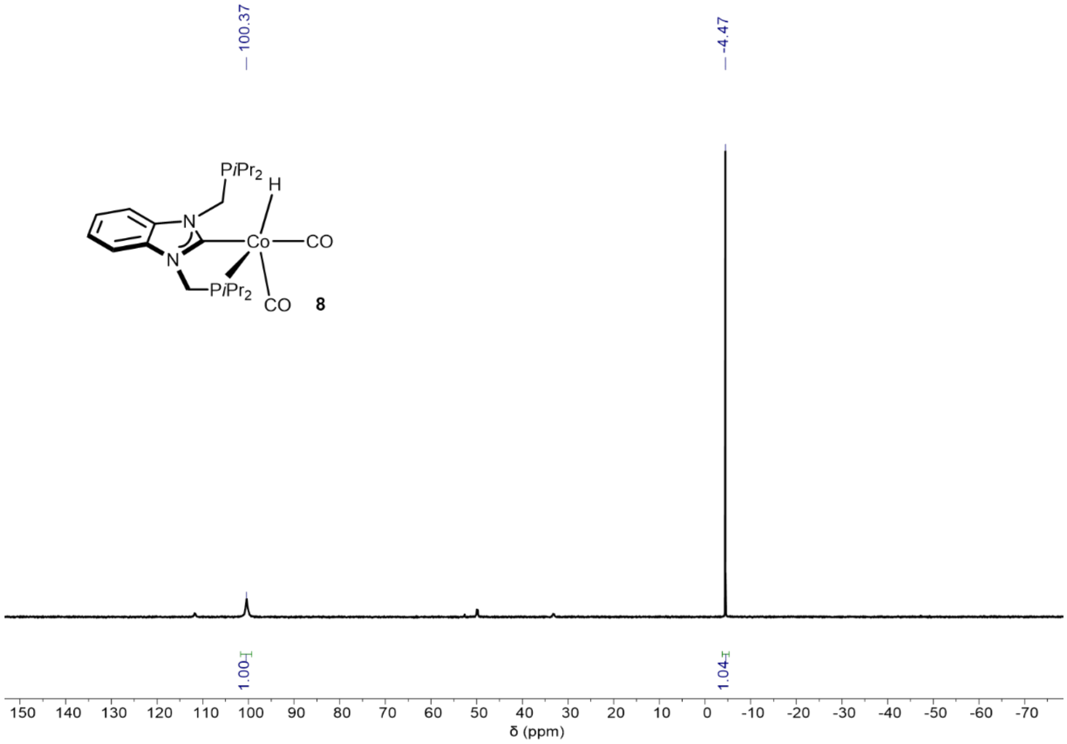

Figure S31 $^{31}P\{^{1}H\}$ NMR spectrum (193 MHz, C_6D_6) of complex **[Co(PCP-*i*Pr)H(CO)$_2$]** (**8**).

Characterization data for the products isolated from the catalytic runs

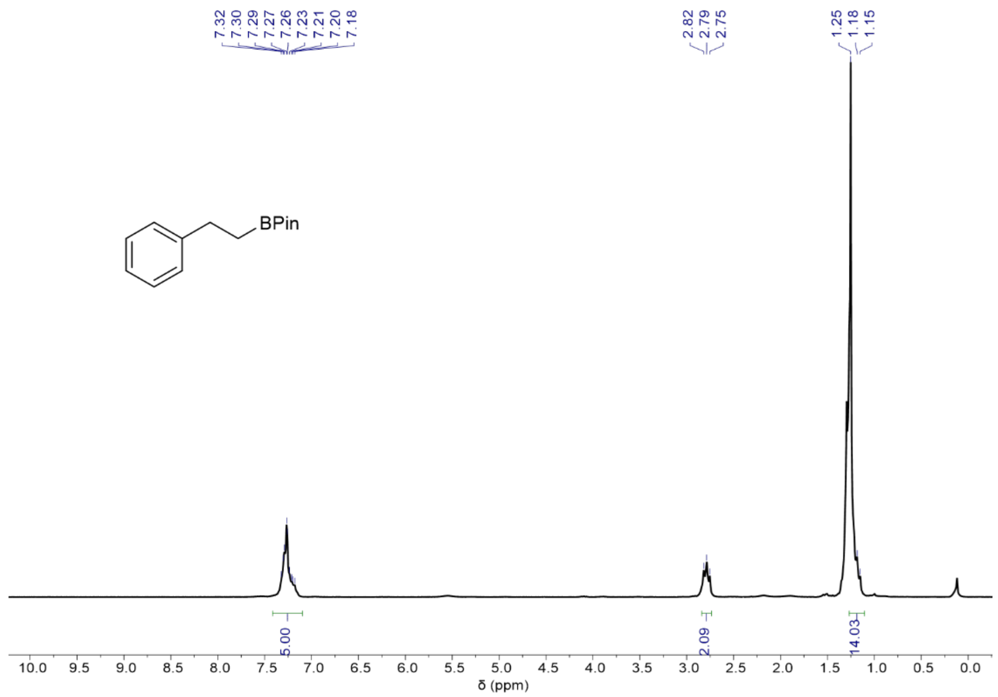

Figure S32 ^{1}H NMR spectrum (250 MHz, $CDCl_3$) of the hydroboration product of styrene.

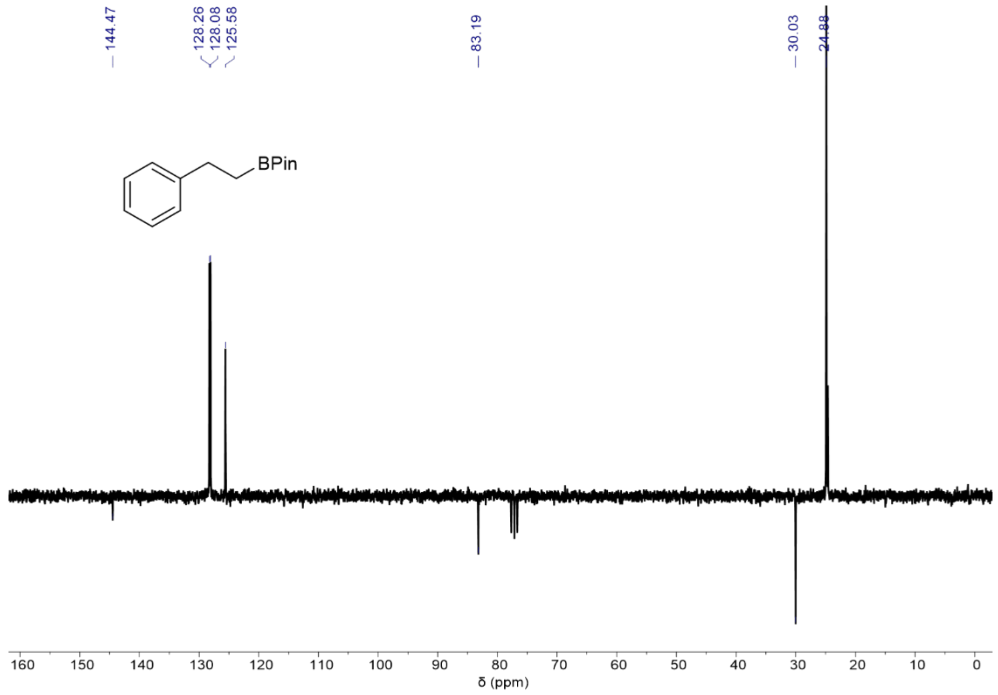

Figure S33 ^{13}C (APT) NMR spectrum (63 MHz, $CDCl_3$) of the hydroboration product of styrene.

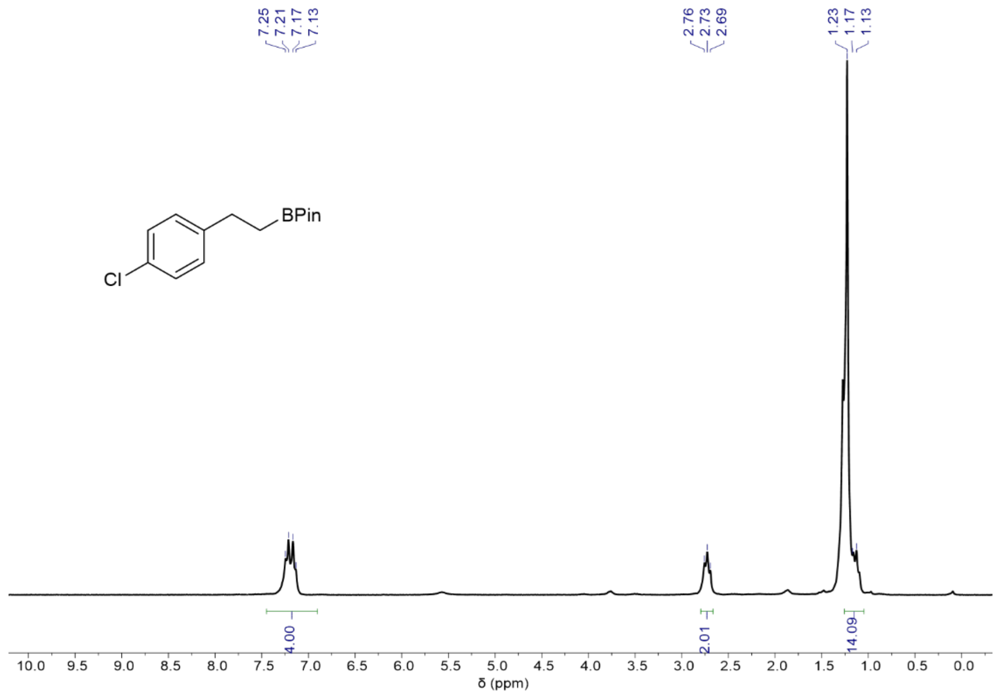

Figure S34 ^{1}H NMR spectrum (250 MHz, $CDCl_3$) of the hydroboration product of 4-chlorostyrene.

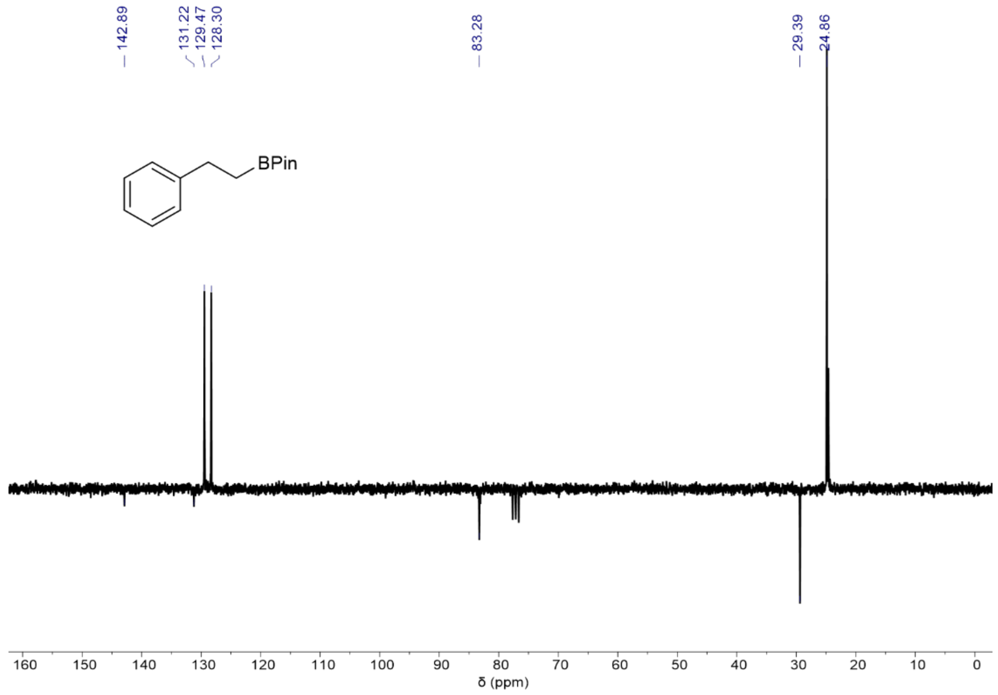

Figure S35 ^{13}C (APT) NMR spectrum (63 MHz, $CDCl_3$) of the hydroboration product of 4-chlorostyrene.

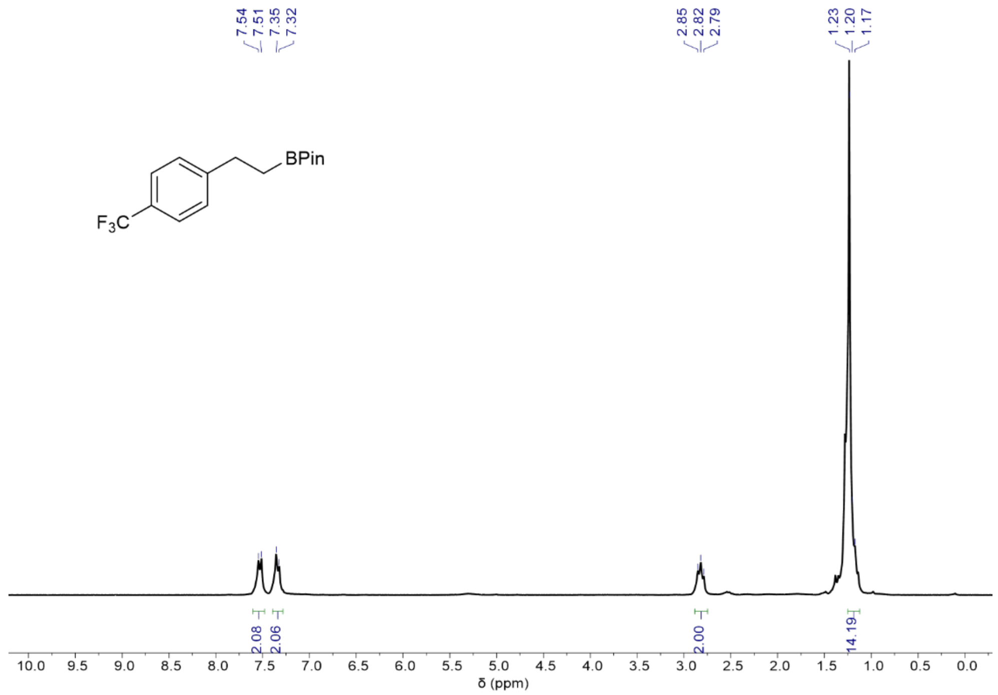

Figure S36 ^{1}H NMR spectrum (250 MHz, $CDCl_3$) of the hydroboration product of 4-(trifluoromethyl)styrene.

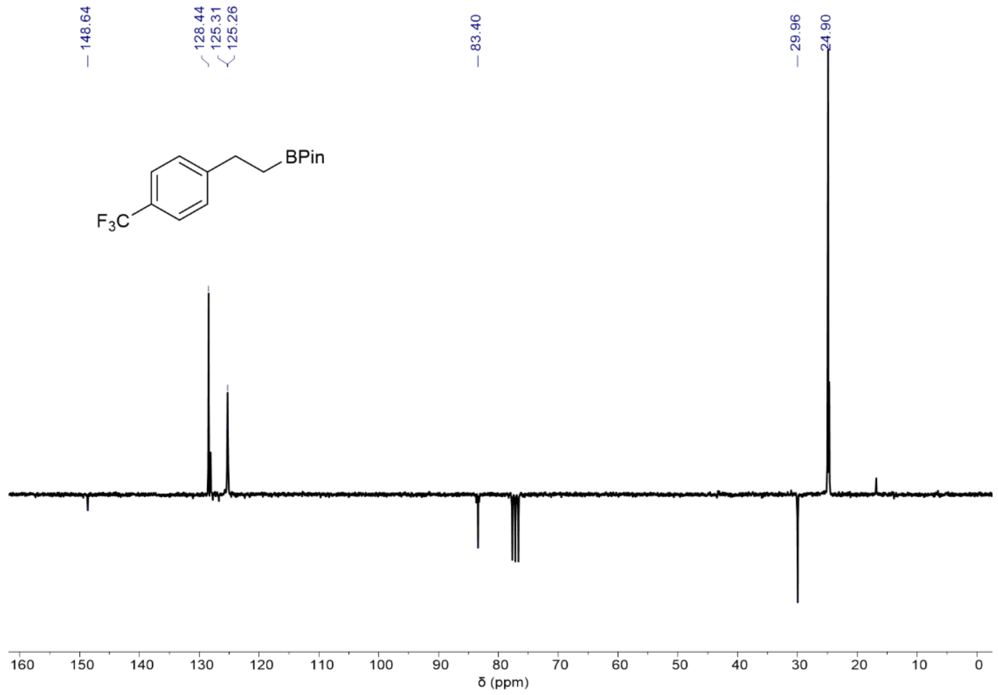

Figure S37 ^{13}C (APT) NMR spectrum (63 MHz, $CDCl_3$) of the hydroboration product of 4-(trifluoromethyl)styrene.

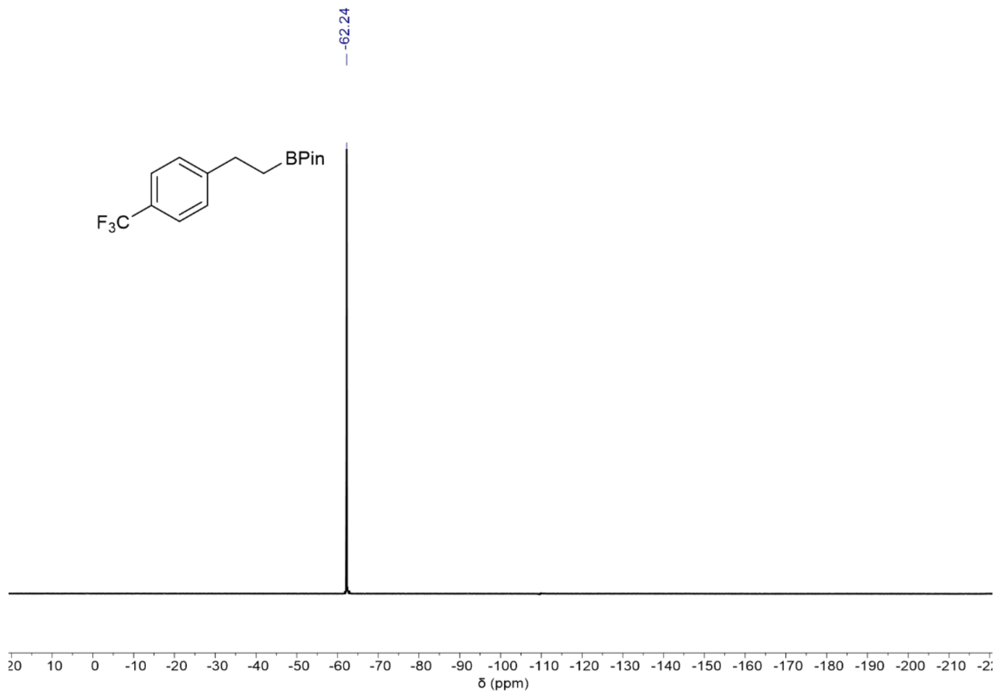

Figure S38 $^{19}F\{^{1}H\}$ NMR spectrum (235 MHz, $CDCl_3$) of the hydroboration product of 4-(trifluoromethyl)styrene.

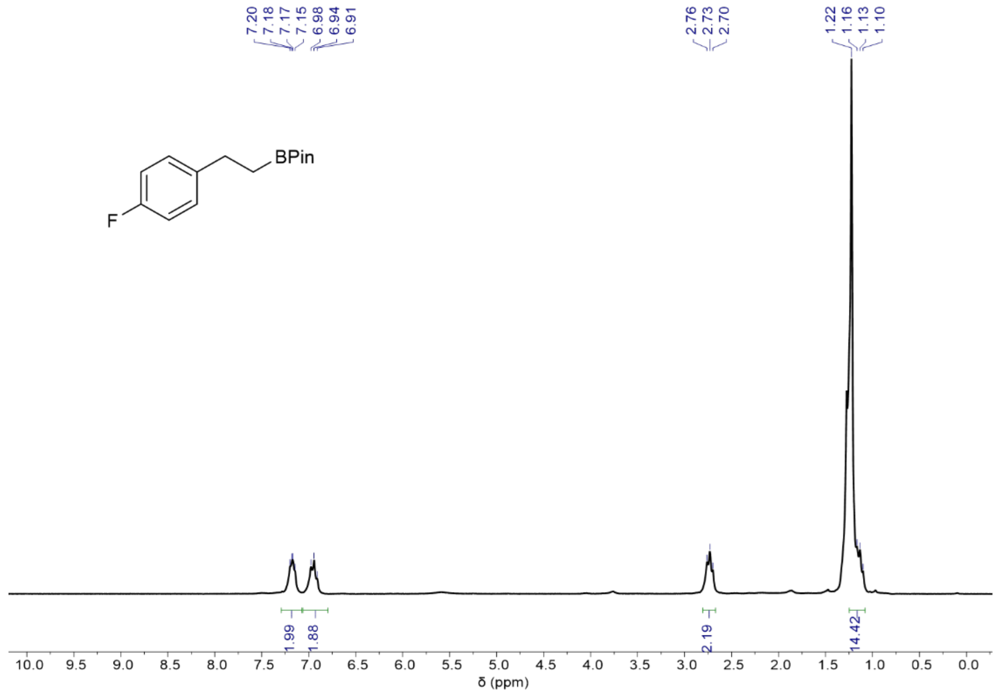

Figure S39 ^{1}H NMR spectrum (250 MHz, $CDCl_3$) of the hydroboration product of 4-fluorostyrene.

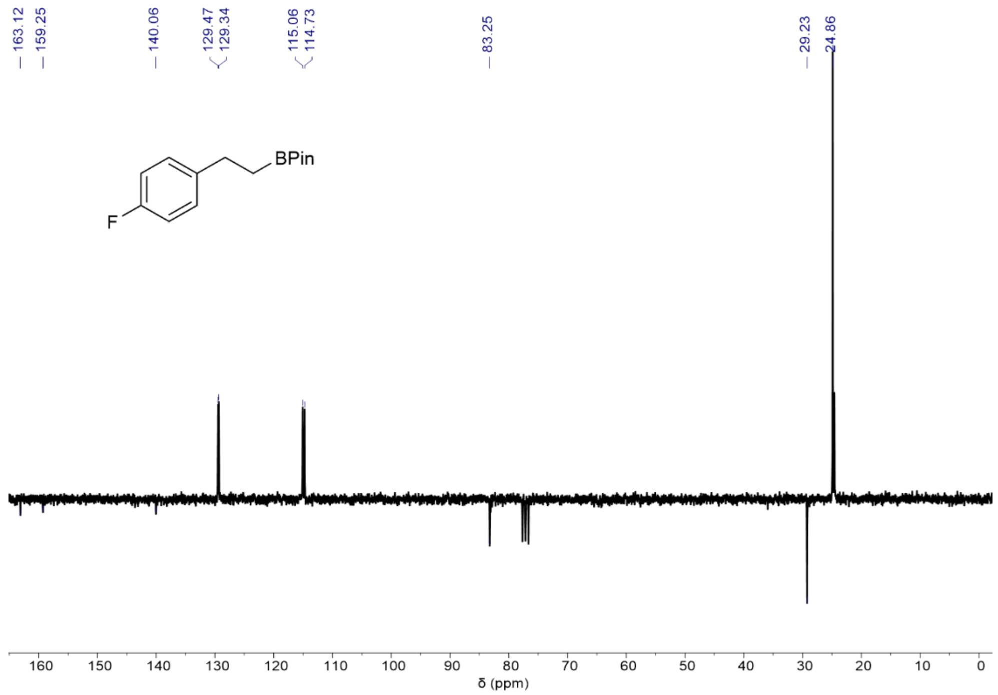

Figure S40 ^{13}C (APT) NMR spectrum (63 MHz, $CDCl_3$) of the hydroboration product of 4-fluorostyrene.

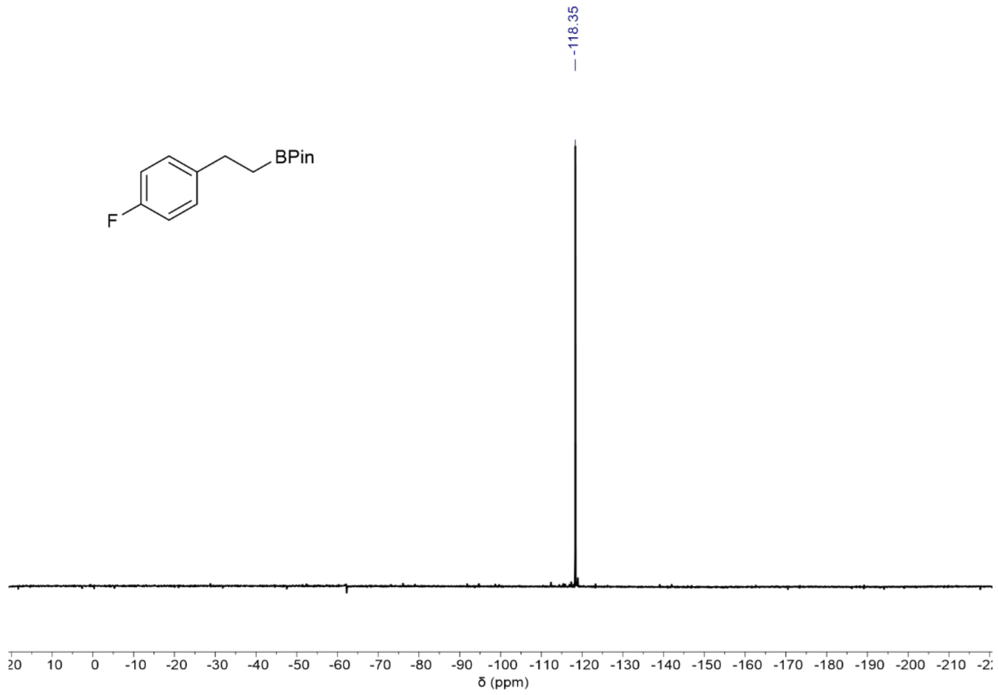

Figure S41 $^{19}F\{^1H\}$ NMR spectrum (235 MHz, $CDCl_3$) of the hydroboration product of 4-fluorostyrene.

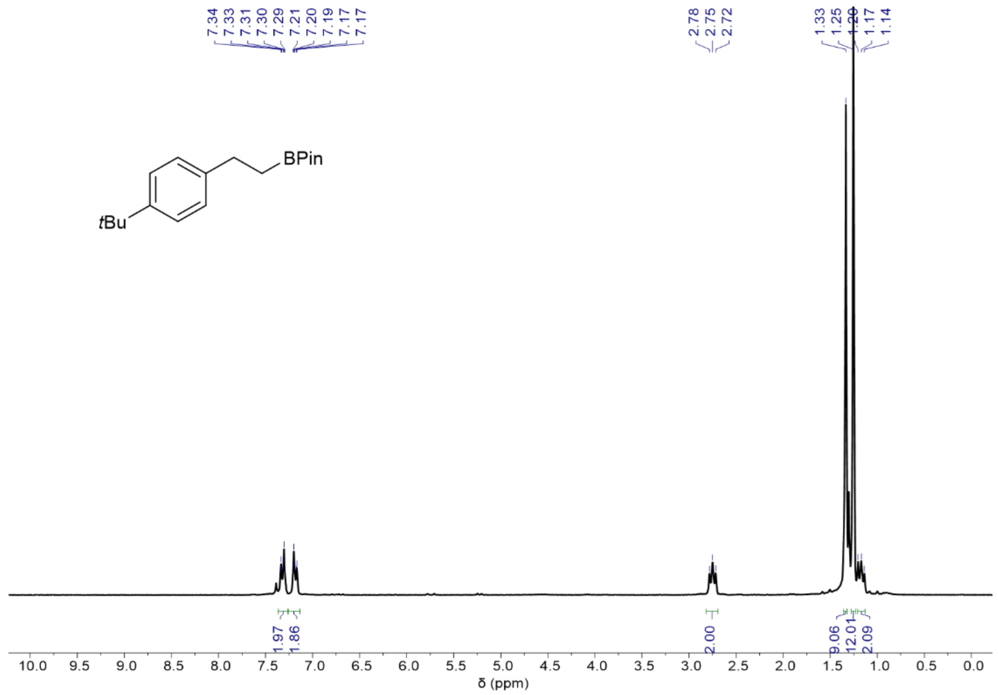

Figure S42 ^{1}H NMR spectrum (250 MHz, CDCl$_3$) of the hydroboration product of 4-*tert*-butylstyrene.

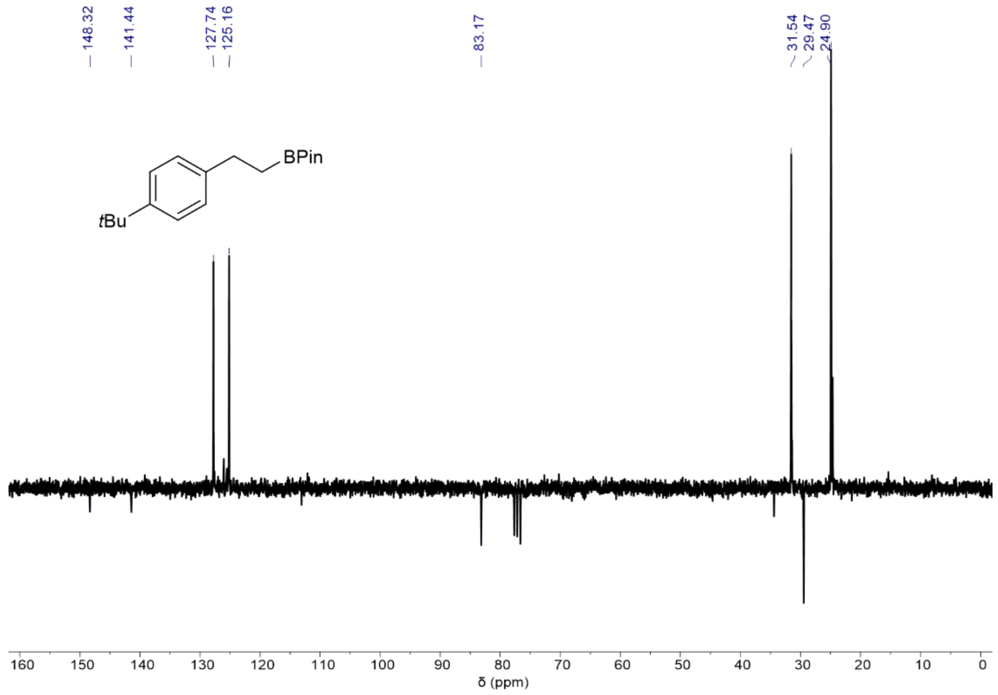

Figure S43 ^{13}C (APT) NMR spectrum (63 MHz, CDCl$_3$) of the hydroboration product of 4-*tert*-butylstyrene.

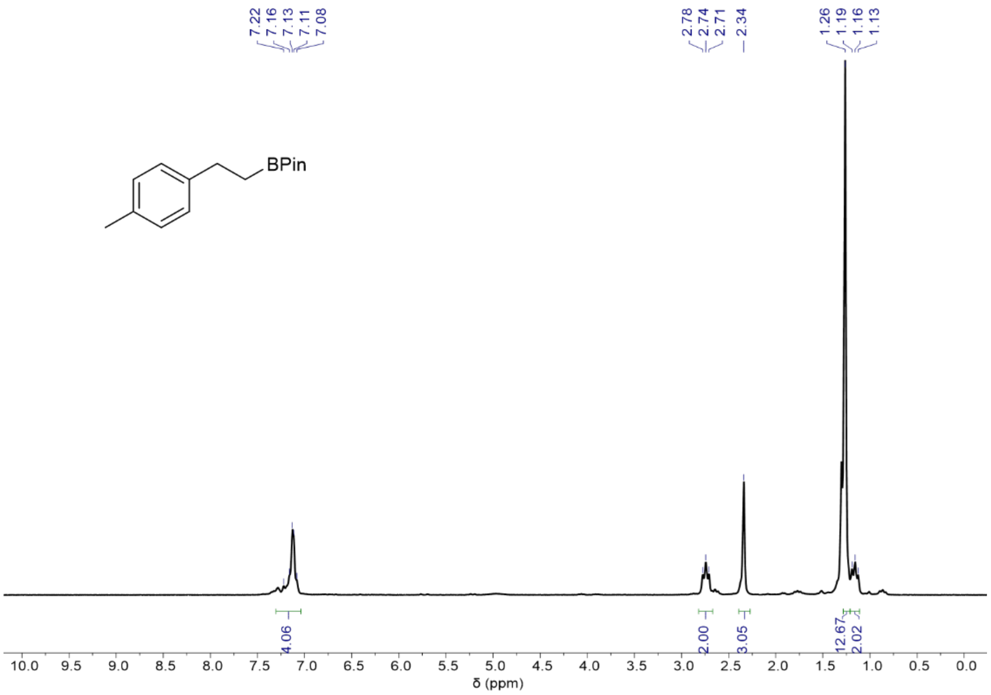

Figure S44 ^{1}H NMR spectrum (250 MHz, $CDCl_3$) of the hydroboration product of 4-methylstyrene.

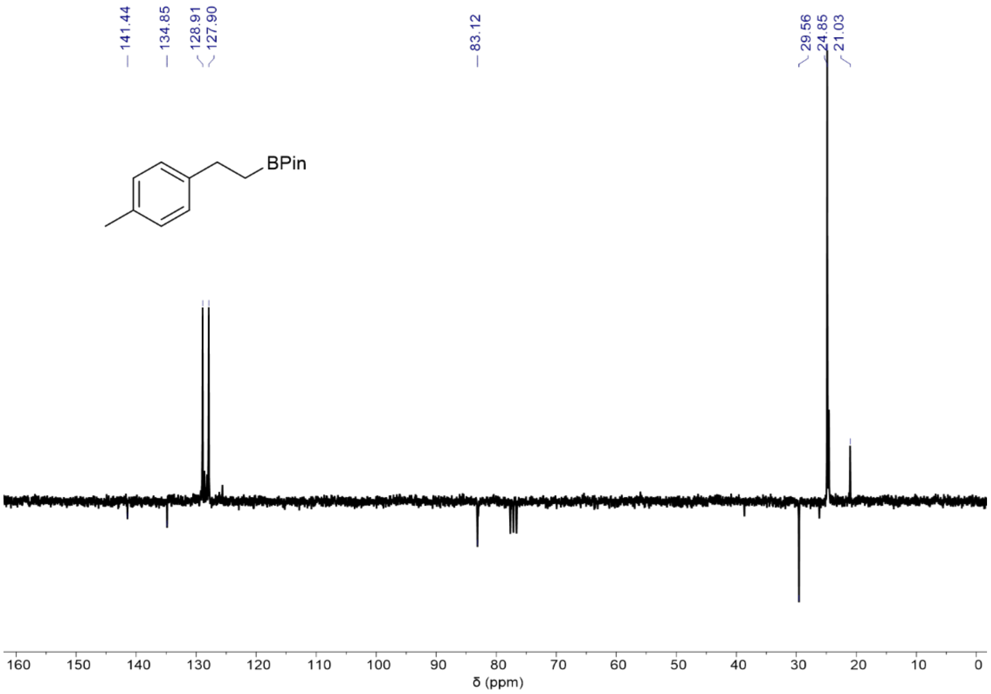

Figure S45 ^{13}C (APT) NMR spectrum (63 MHz, $CDCl_3$) of the hydroboration product of 4-methylstyrene.

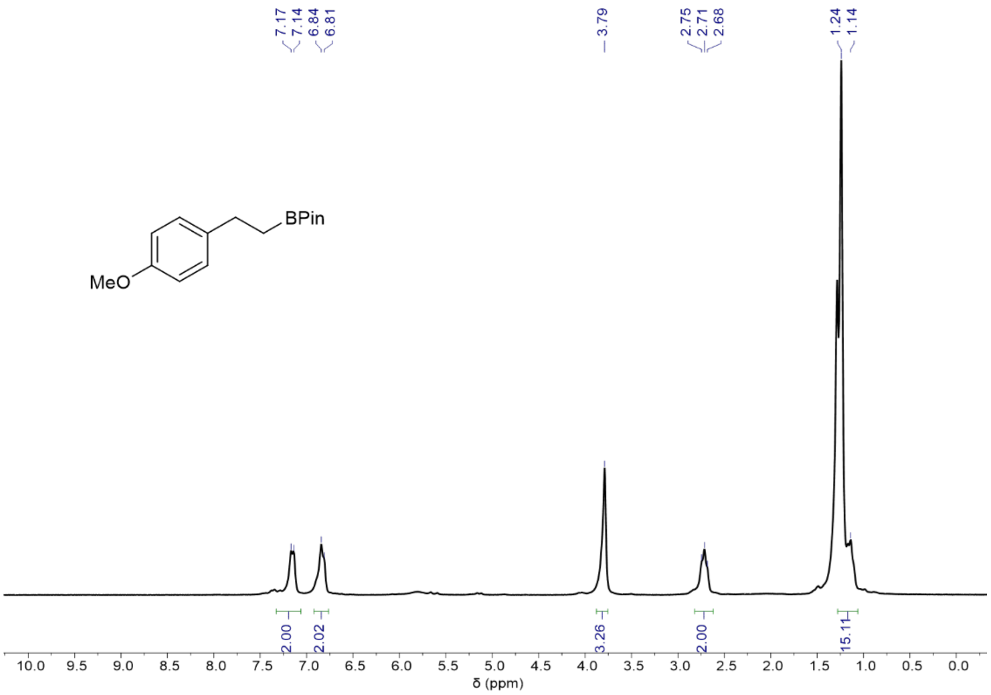

Figure S46 ^{1}H NMR spectrum (250 MHz, $CDCl_3$) of the hydroboration product of 4-methoxystyrene.

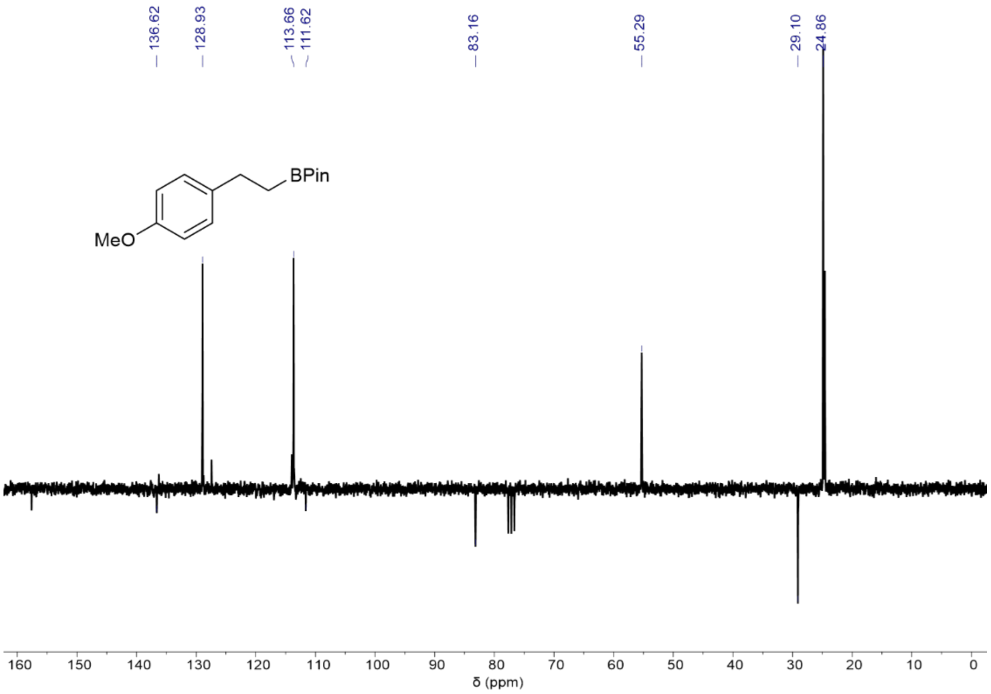

Figure S47 ^{13}C (APT) NMR spectrum (63 MHz, $CDCl_3$) of the hydroboration product of 4-methoxystyrene.

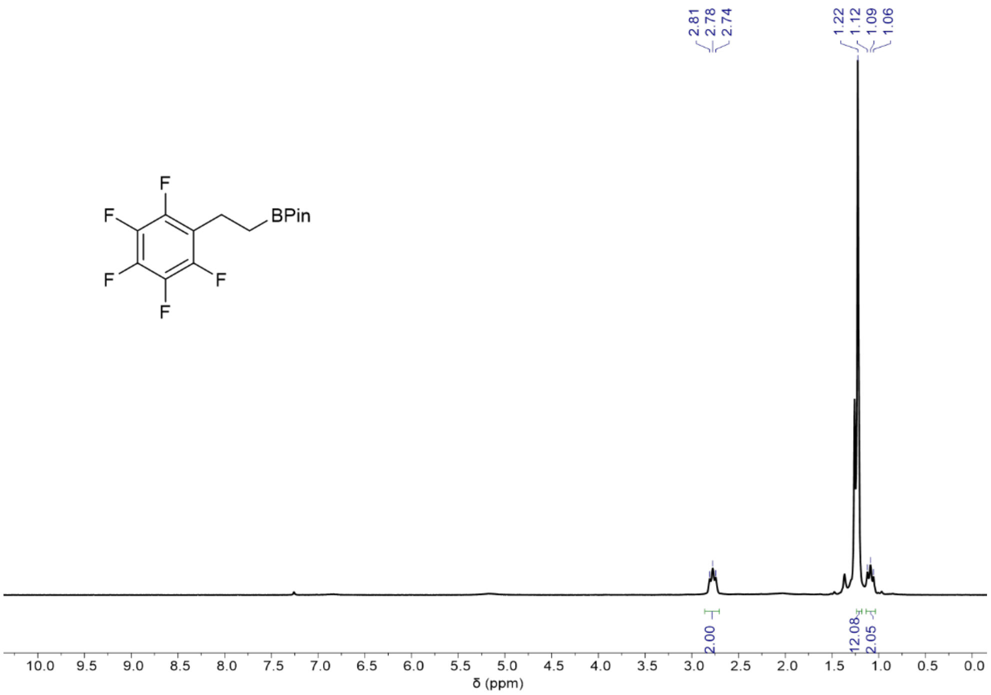

Figure S48 ^{1}H NMR spectrum (250 MHz, $CDCl_3$) of the hydroboration product of pentafluorophenylstyrene.

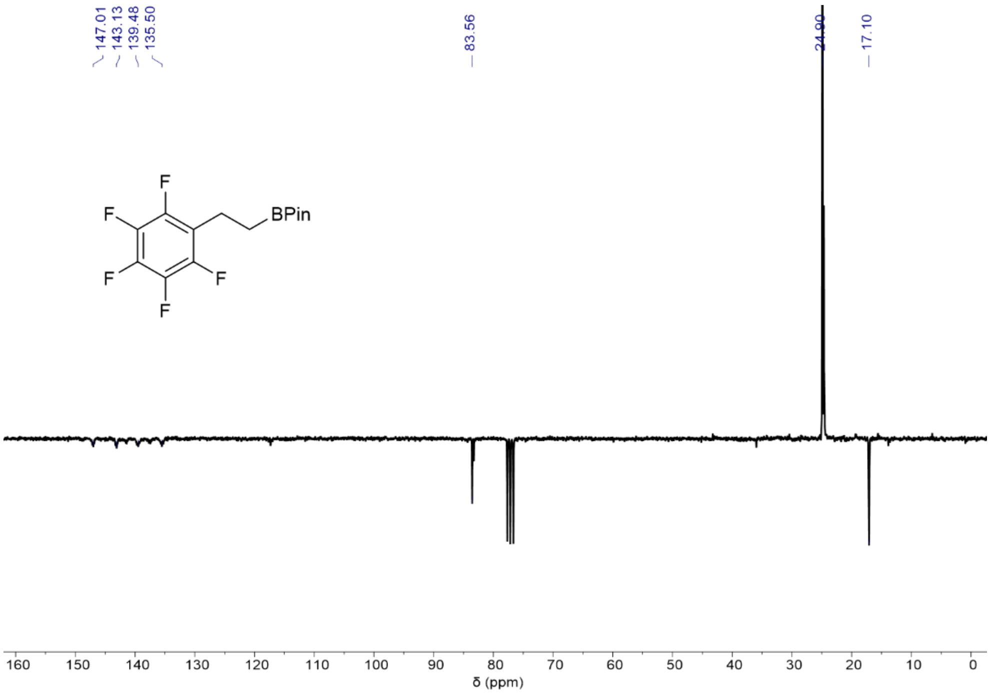

Figure S49 ^{13}C (APT) NMR spectrum (63 MHz, $CDCl_3$) of the hydroboration product of pentafluorophenylstyrene.

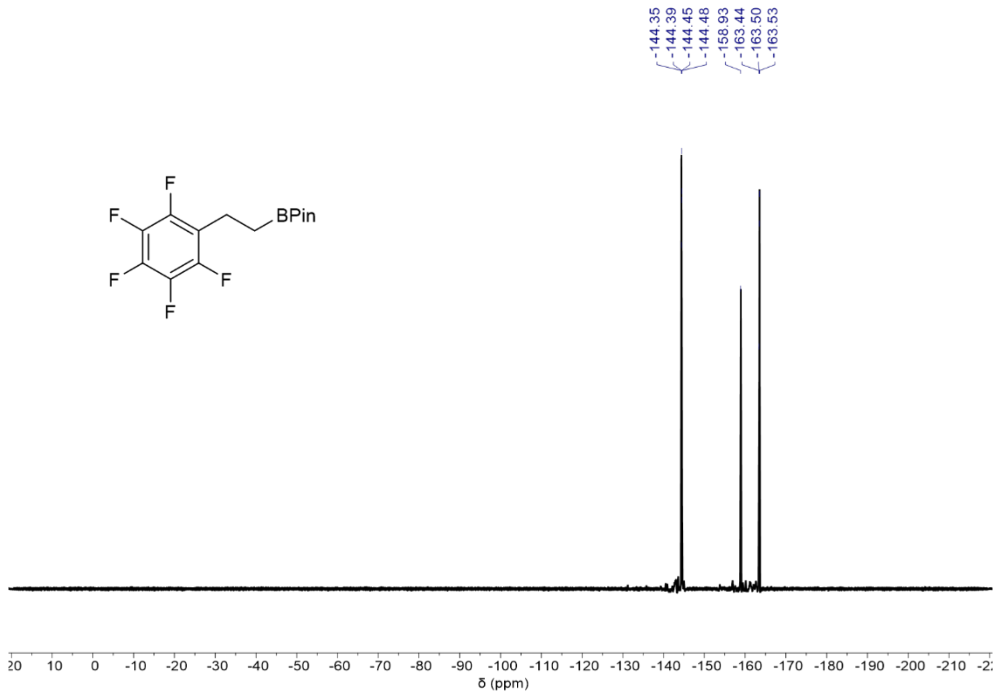

Figure S50 $^{19}F\{^{1}H\}$ NMR spectrum (235 MHz, $CDCl_3$) of the hydroboration product of 4-(trifluoromethyl)styrene.

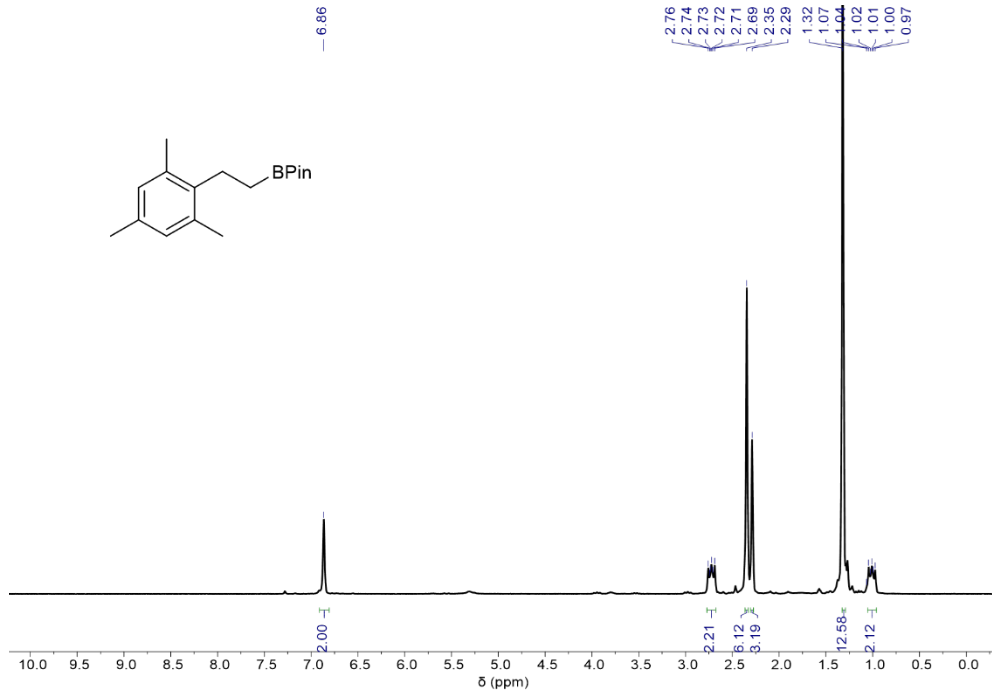

Figure S51 ^{1}H NMR spectrum (250 MHz, $CDCl_3$) of the hydroboration product of mesitylstyrene.

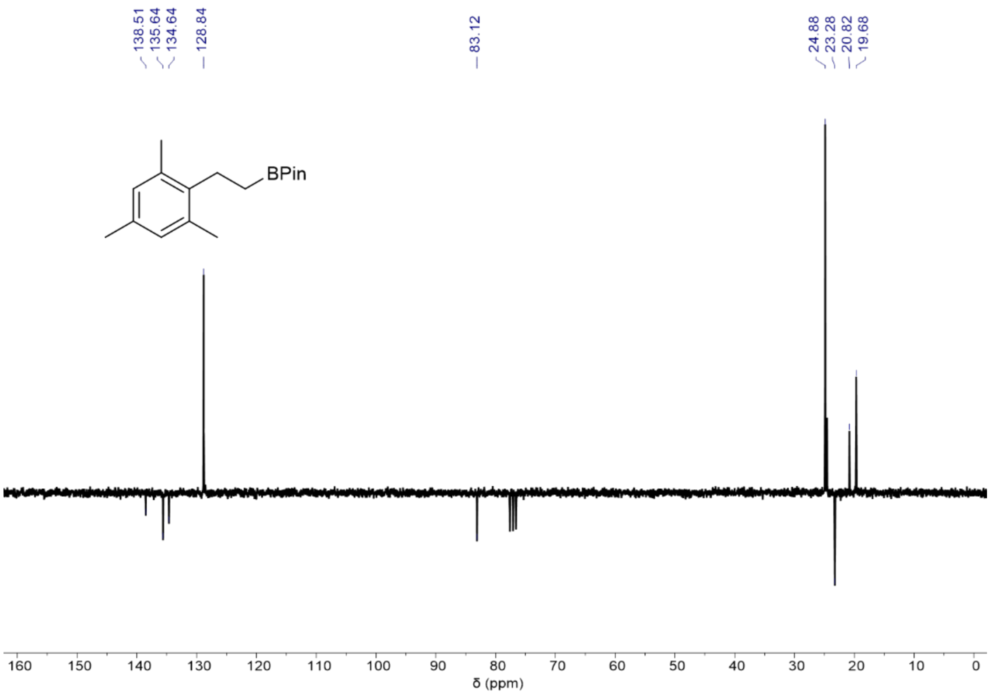

Figure S52 ^{13}C (APT) NMR spectrum (63 MHz, $CDCl_3$) of the hydroboration product of mesitylstyrene.

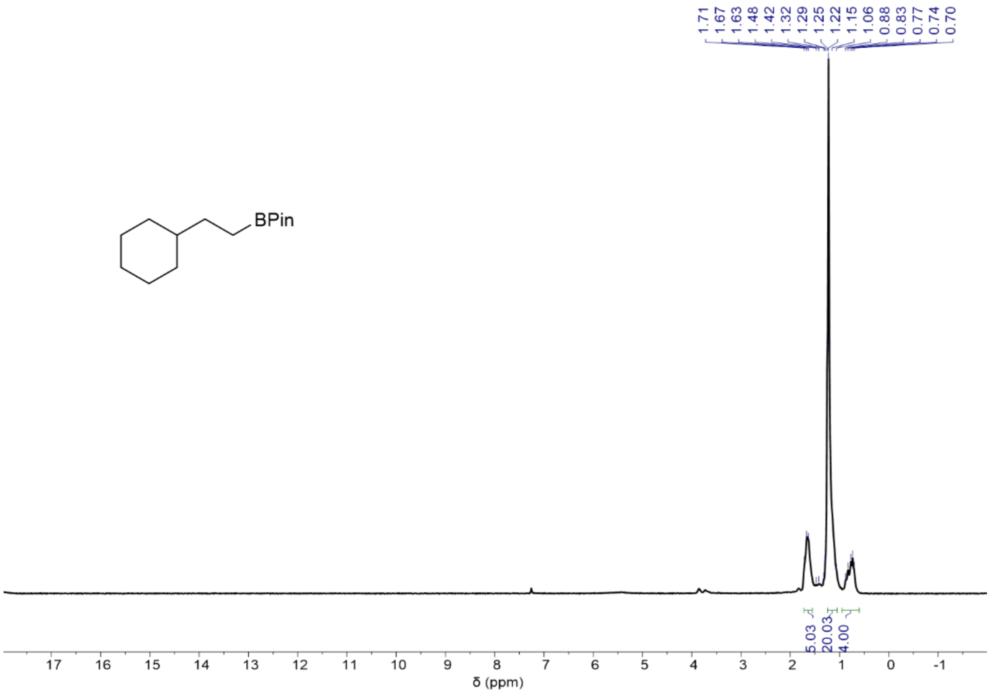

Figure S53 ^{1}H NMR spectrum (250 MHz, $CDCl_3$) of the hydroboration product of vinylcyclohexane.

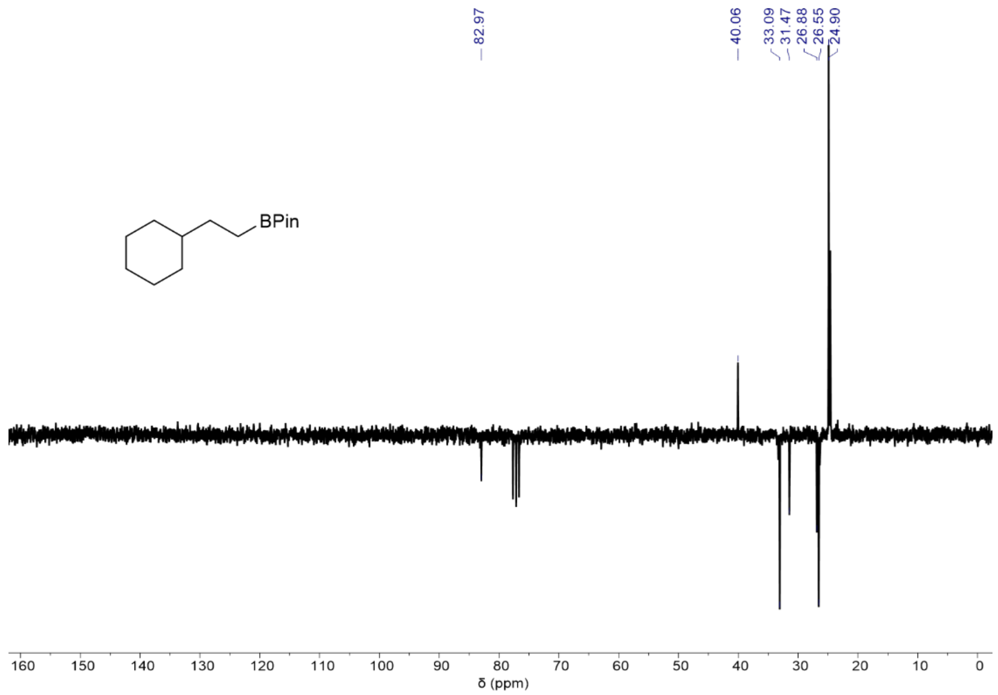

Figure S54 ^{13}C (APT) NMR spectrum (63 MHz, $CDCl_3$) of the hydroboration product of vinylcyclohexane.

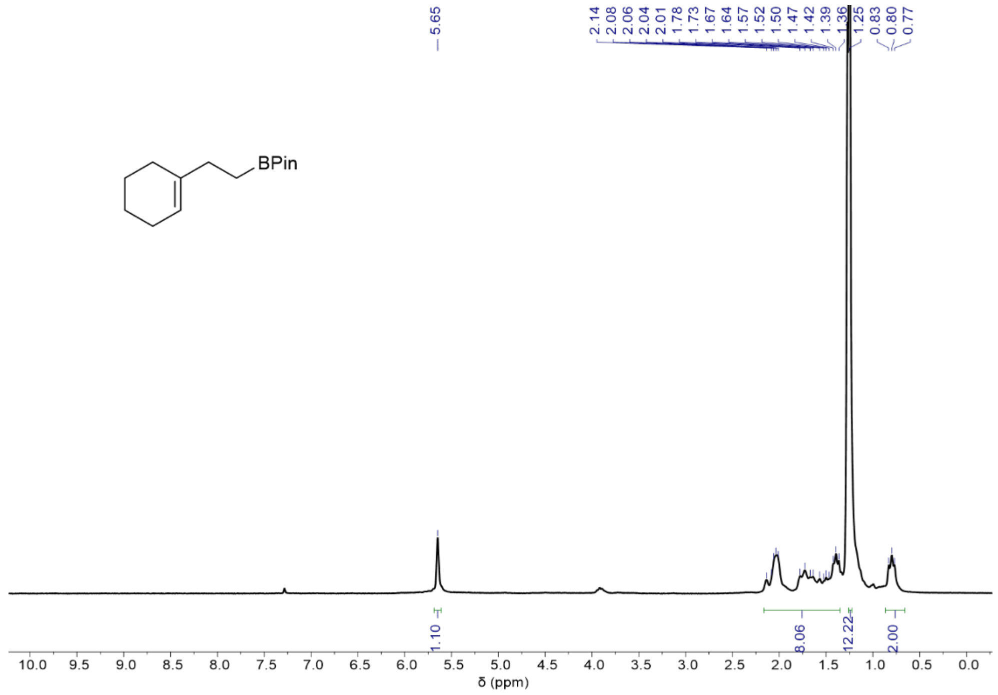

Figure S55 ^{1}H NMR spectrum (250 MHz, $CDCl_3$) of the hydroboration product of 4-vinylcyclohex-1-ene.

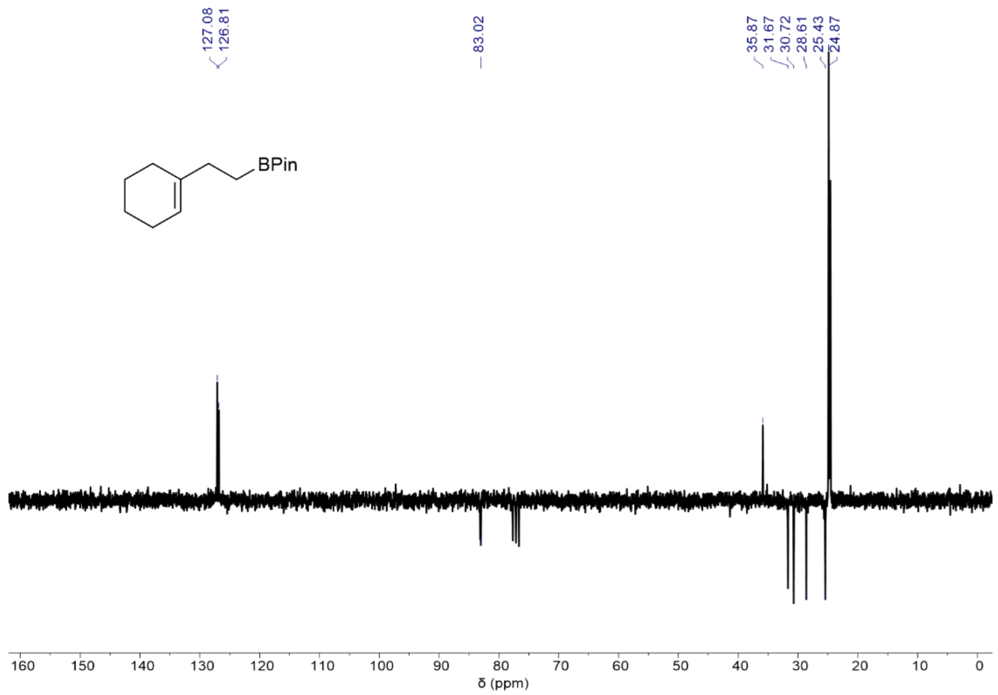

Figure S56 ^{13}C (APT) NMR spectrum (63 MHz, $CDCl_3$) of the hydroboration product of 4-vinylcyclohex-1-ene.

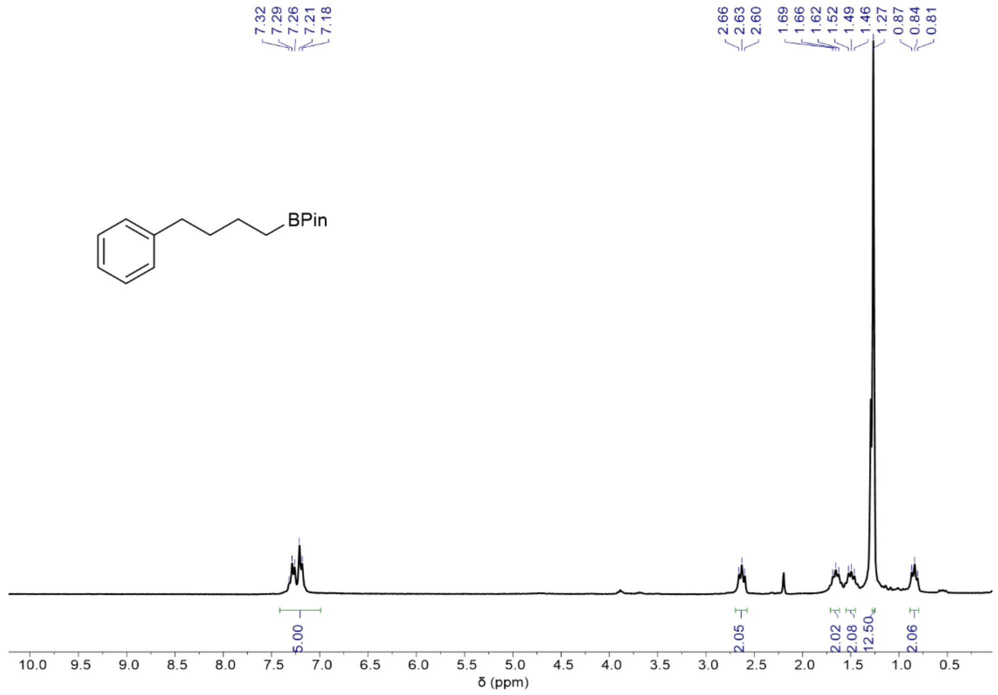

Figure S57 ^{1}H NMR spectrum (250 MHz, $CDCl_3$) of the hydroboration product of but-3-en-1-ylbenzene.

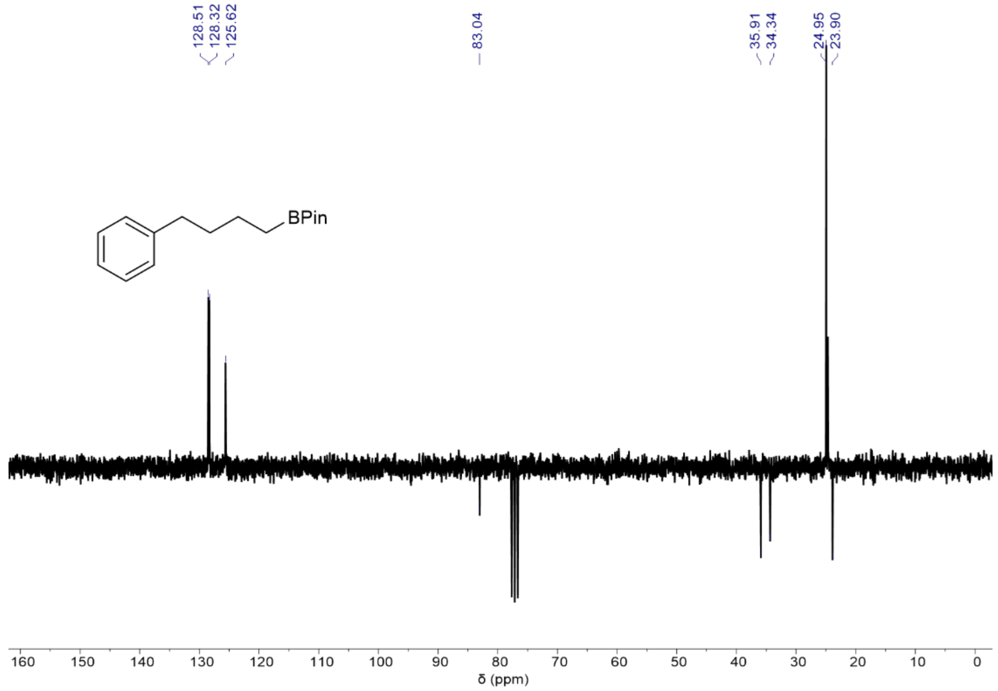

Figure S58 ^{13}C (APT) NMR spectrum (63 MHz, $CDCl_3$) of the hydroboration product of but-3-en-1-ylbenzene.

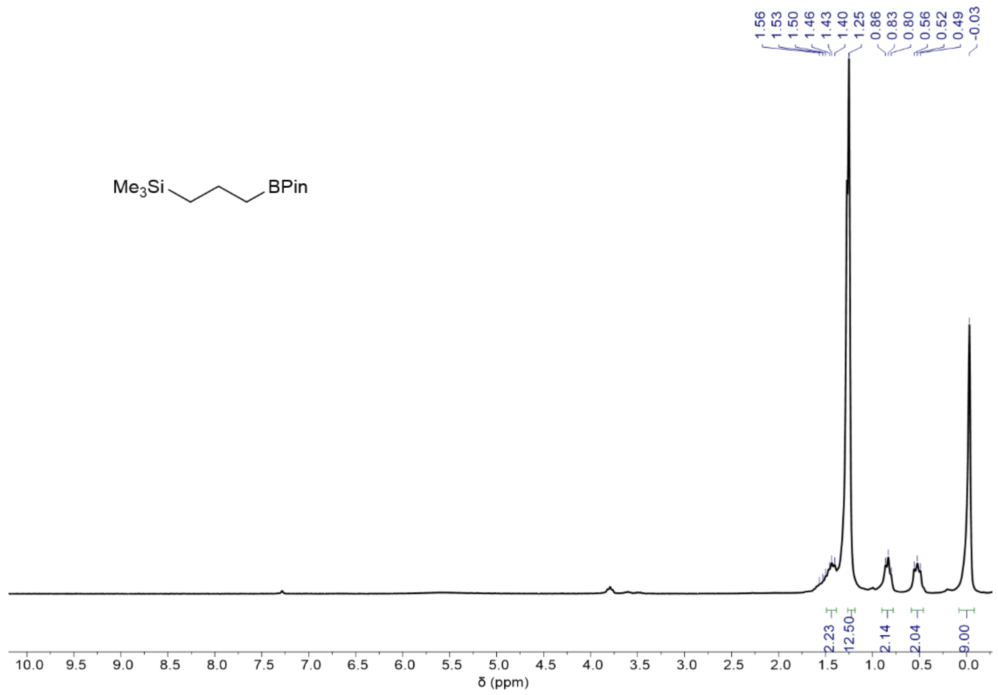

Figure S59 ^{1}H NMR spectrum (250 MHz, $CDCl_3$) of the hydroboration product of allyltrimethylsilane.

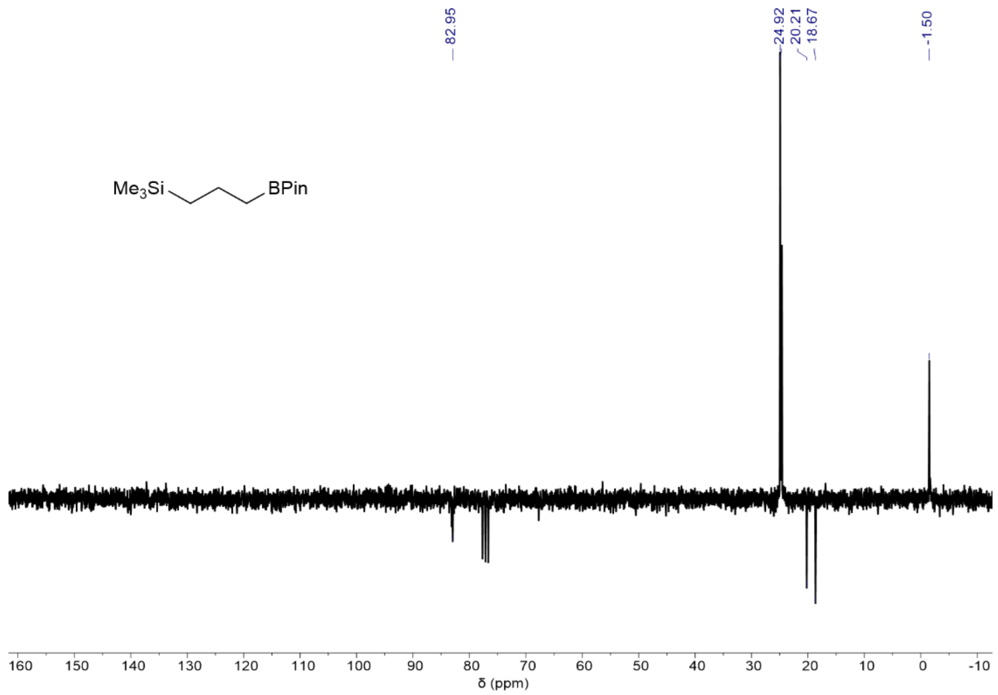

Figure S60 ^{13}C (APT) NMR spectrum (63 MHz, $CDCl_3$) of the hydroboration product of allyltrimethylsilane.

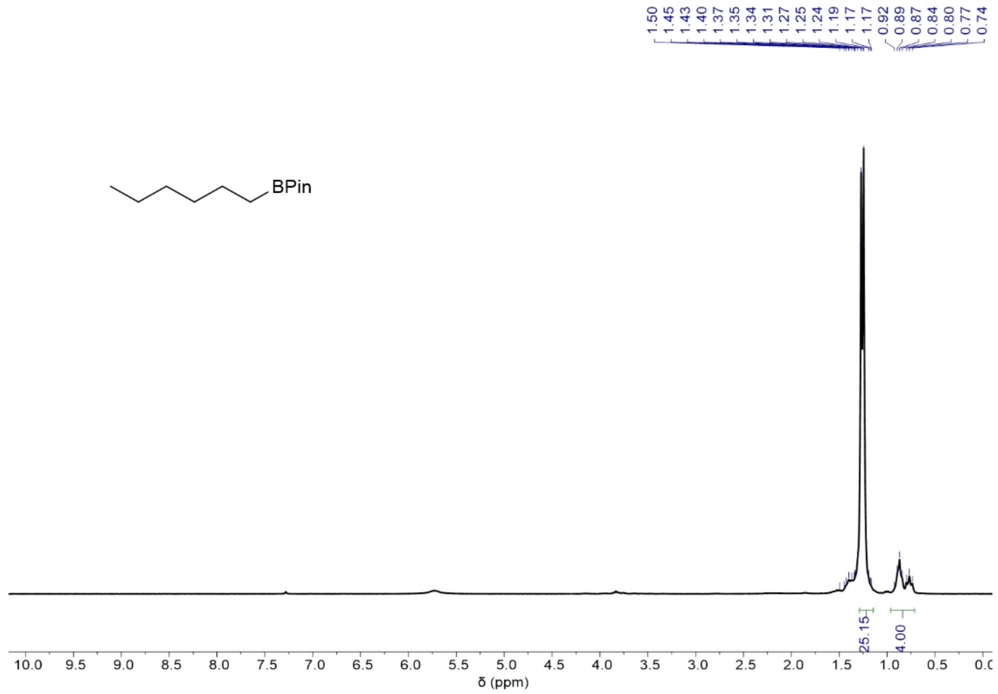

Figure S61 ^{1}H NMR spectrum (250 MHz, $CDCl_3$) of the hydroboration product of 1-hexene.

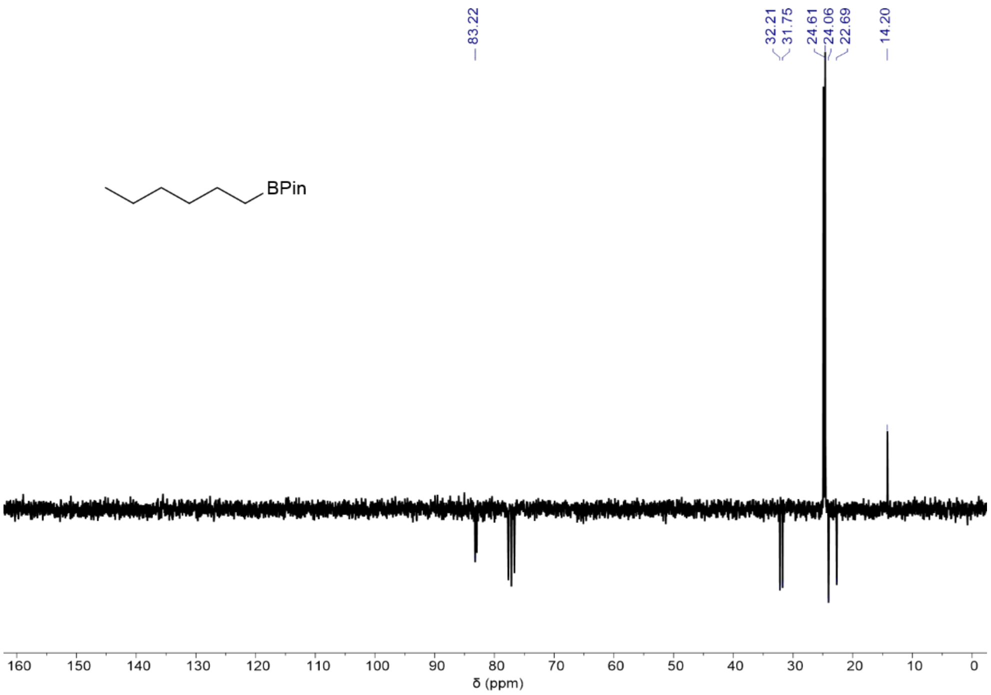

Figure S62 ^{13}C (APT) NMR spectrum (63 MHz, $CDCl_3$) of the hydroboration product of 1-hexene.

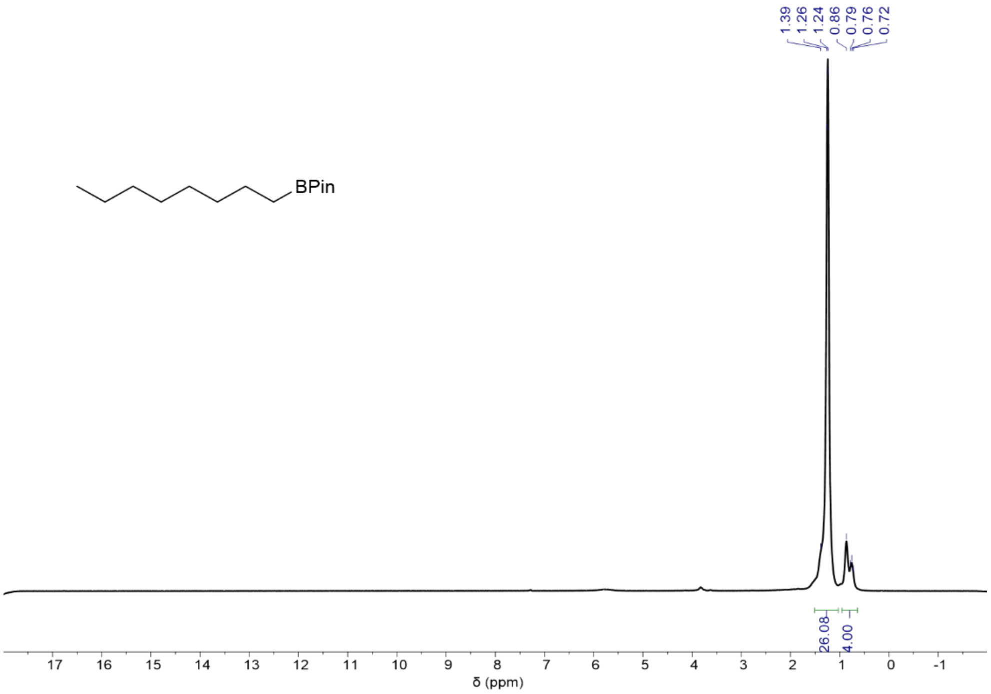

Figure S63 ^{1}H NMR spectrum (250 MHz, $CDCl_3$) of the hydroboration product of 1-octene.

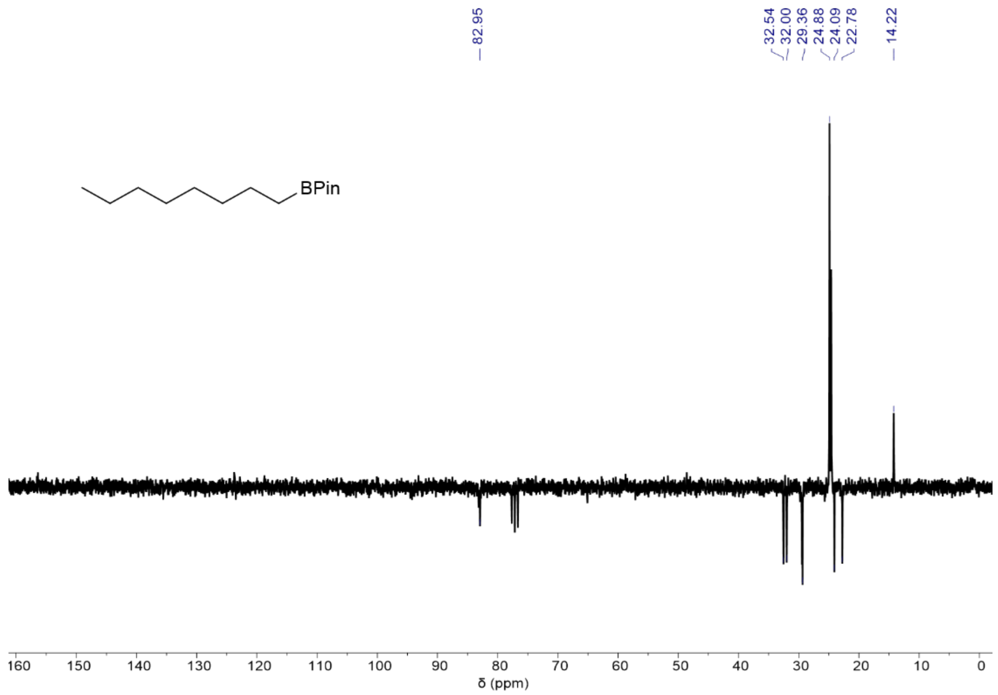

Figure S64 ^{13}C (APT) NMR spectrum (63 MHz, $CDCl_3$) of the hydroboration product of 1-octene.
